# Supplementary material for: Piltunines A–F from the Marine-Derived Fungus Penicillium piltunense KMM 4668
Source: Mar Drugs. 2019 Nov 18;17(11):647. doi: 10.3390/md17110647 (PMC6891813; doi:10.3390/md17110647)
Supplement: Supplementary file 1 [file marinedrugs-17-00647-s001.pdf]

SUPPLEMENTARY MATERIAL

Piltunines A–F from the marine-derived fungus *Penicillium piltunense* KMM 4668

Shamil Sh. Afiyatullov[1,*], Olesya I. Zhuravleva[1,2], Alexandr S. Antonov [1], Elena V. Leshchenko [1,2],Mikhail V. Pivkin[1], Yuliya V Khudyakova[1], Vladimir A. Denisenko [1],Evgeny A. Pislyagin[1], Natalya Yu. Kim [1], Dmitrii V. Berdyshev[1], Gunhild von Amsberg[3,4] and Sergey A. Dyshlovoy[1,2,3,4]

[1] G.B. Elyakov Pacific Institute of Bioorganic Chemistry, Far Eastern Branch of the Russian Academy of Sciences, Prospect 100-letiya Vladivostoka, 159, Vladivostok 690022, Russia; zhuravleva.oi@dvfu.ru (O.I.Z.);alexanderantonovpiboc@gmail.com (A.S.A.); bykadorovachem@gmail.com (L.E.V.);oid27@mail.ru (M.V.P.); 161070@rambler.ru (Y.V.K);vladenis@pidoc.dvo.ru (V.A.D.); pislyagin@hotmail.com (E.A.P.); natalya_kim@mail.ru (N.Y.K.); berdyshev@piboc.dvo.ru (D.V.B.); dyshlovoy@gmail.com (S.A.D.)

[2] School of Natural Science, Far Eastern Federal University, Sukhanova St., 8, Vladivostok 690000, Russia

[3] Laboratory of Experimental Oncology, Department of Oncology, Hematology and Bone Marrow Transplantation with Section Pneumology, Hubertus Wald-Tumorzentrum, University Medical Center Hamburg-Eppendorf, 20246 Hamburg, Germany; g.von-amsberg@uke.de(G.A.)

[4] Martini-Klinik Prostate Cancer Center, University Hospital Hamburg-Eppendorf, 20246 Hamburg, Germany

* Correspondence: afiyat@piboc.dvo.ru;zhuravleva.oi@dvfu.ru; Tel.: +7-423-231-1168

Piltunines A–F from the marine-derived fungus *Penicillium piltunense* KMM 4668

Six new carotane sesquiterpenoids piltunines A–F (**1**–**6**) together with known compounds (**7**–**9**) were isolated from the marine-derived fungus *Penicillium piltunense* KMM 4668. Their structures were established using spectroscopic methods. The absolute configurations of **1**–**7** were determined based on CD and NOESY data as well as biogenetic considerations. The cytotoxic activity of some of the isolated compounds and their effects on regulation of ROS and NO production in lipopolysaccharide-stimulated macrophages were examined.

Keywords: *Penicillium piltunense*, secondary metabolites, carotane sesquiterpenoids, cytotoxic activity

Content

Experimental Section.. 6
Table S1. NMR Data for compounds**1a** and **7**.. 5
Table S2. NMR Data for compounds**1, 2** and **3**.. 6
Table S3. NMR Data for compounds**4, 5** and **6**.. 7
Figure S1. CD spectrum of **1**……………………………………………...….......…......……...…8
Figure S2. CD spectrum of **2**……………………………………………..………………...…8
Figure S3. CD spectrum of **3** 9
Figure S4. CD spectrum of **4** . ………………………………………………………………..9
Figure S5. CD spectrum of **5**………………………………………………………...…...10
Figure S6. CD spectrum of **6** 10
Figure S7. CD spectrum of **7** 11
Figure S8. UV spectrum of **1**………………………………………………………………..11
Figure S9. UV spectrum of **2**………………………………………………………………..12
Figure S10. UV spectrum of **3**……………………………………………………………12
Figure S11. UV spectrum of **4**……………………………………………………………13
Figure S12. UV spectrum of **5** 13
Figure S13. UV spectrum of **6** 14
Figure S14. IR spectrum of **1** (3500 – 1000 cm^{-1})…………………………………………..15
Figure S15. IR spectrum of **1** (1900 – 1000 cm^{-1})…………………………………………..16
Figure S16. IR spectrum of **2** (3500 – 1000 cm^{-1})…………………………………………..17
Figure S17. IR spectrum of **2** (1900 – 1000 cm^{-1})…………………………………………..18
Figure S18. IR spectrum of **4** (3500 – 1000 cm^{-1})…………………………………………..19
Figure S19. IR spectrum of **4** (1900 – 1000 cm^{-1})…………………………………………..20
Figure S20. IR spectrum of **6** (3500 – 1000 cm^{-1})…………………………………………..21
Figure S21. IR spectrum of **6** (1900 – 1000 cm^{-1})…………………………………………..22
Figure S22. ^{1}H NMR spectrum (700 MHz, CD_3OD) of **1** 23
Figure S23. ^{13}C NMR spectrum (176 MHz, CD_3OD) of **1** 24
Figure S24. DEPT-135 NMR spectrum (176 MHz, CD_3OD) of **1**…………………………………25
Figure S25. COSY-45 spectrum (700 MHz, CD_3OD) of **1** 26
Figure S26. HSQC spectrum (700 MHz, CD_3OD) of **1**... 27
Figure S27. HMBC spectrum (700 MHz, CD_3OD) of **1**.. 28
Figure S28. NOESY spectrum (700 MHz, CD_3OD) of **1** 29
Figure S29. ^{1}H NMR spectrum (500 MHz, CD_3OD) of **1a** 30
Figure S30. ^{13}C NMR spectrum (125 MHz, CD_3OD) of **1a** 31
Figure S31. HSQC spectrum (500 MHz, CD_3OD) of **1a**... 32
Figure S32. HMBC spectrum (500 MHz, CD_3OD) of **1a**.. 33
Figure S33. ^{1}H NMR spectrum (700 MHz, CD_3OD) of **2** 34
Figure S34. ^{13}C NMR spectrum (176 MHz, CD_3OD) of **2** 35
Figure S35. DEPT-135 NMR spectrum (176 MHz, CD_3OD) of **2**…………………………………36
Figure S36. COSY-45 spectrum (700 MHz, CD_3OD) of **2**…………………………………………37
Figure S37. HSQC spectrum (700 MHz, CD_3OD) of **2**... 38
Figure S38. HMBC spectrum (700 MHz, CD_3OD) of **2**.. 39

Figure S39. NOESY spectrum (700 MHz, CD_3OD) of **2** 40
Figure S40. ^{1}H NMR spectrum (700 MHz, CD_3OD) of **3** 41
Figure S41. ^{13}C NMR spectrum (176 MHz, CD_3OD) of **3** 42
Figure S42. DEPT-135 NMR spectrum (176 MHz, CD_3OD) of **3**........43
Figure S43. COSY-45 spectrum (700 MHz, CD_3OD) of **3**........44
Figure S44. HSQC spectrum (700 MHz, CD_3OD) of **3**........ 45
Figure S45. HMBC spectrum (700 MHz, CD_3OD) of **3**........ 46
Figure S46. NOESY spectrum (700 MHz, CD_3OD) of **3** 47
Figure S47. ^{1}H NMR spectrum (700 MHz, CD_3OD) of **4** 48
Figure S48. ^{13}C NMR spectrum (176 MHz, CD_3OD) of **4** 49
Figure S49. DEPT-135 NMR spectrum (176 MHz, CD_3OD) of **4**........50
Figure S50. COSY-45 spectrum (700 MHz, CD_3OD) of **4**........51
Figure S51. HSQC spectrum (700 MHz, CD_3OD) of **4**........ 52
Figure S52. HMBC spectrum (500 MHz, CD_3OD) of **4**........ 53
Figure S53. NOESY spectrum (700 MHz, CD_3OD) of **4** 54
Figure S54. ^{1}H NMR spectrum (700 MHz, CD_3OD) of **5** 55
Figure S55. ^{13}C NMR spectrum (176 MHz, CD_3OD) of **5** 56
Figure S56. DEPT-135 NMR spectrum (176 MHz, CD_3OD) of **5**........57
Figure S57. COSY-45 spectrum (700 MHz, CD_3OD) of **5**........58
Figure S58. HSQC spectrum (700 MHz, CD_3OD) of **5**........ 59
Figure S59. HMBC spectrum (700 MHz, CD_3OD) of **5**........ 60
Figure S60. NOESY spectrum (700 MHz, CD_3OD) of **5** 61
Figure S61. ^{1}H NMR spectrum (700 MHz, CD_3OD) of **6** 62
Figure S62. ^{13}C NMR spectrum (176 MHz, CD_3OD) of **6** 63
Figure S63. DEPT-135 NMR spectrum (176 MHz, CD_3OD) of **6**........64
Figure S64. COSY-45 spectrum (700 MHz, CD_3OD) of **6**........65
Figure S65. HSQC spectrum (700 MHz, CD_3OD) of **6**........ 66
Figure S66. HMBC spectrum (700 MHz, CD_3OD) of **6**........ 67
Figure S67. NOESY spectrum (700 MHz, CD_3OD) of **6** 68
Figure S68. ^{1}H NMR spectrum (500 MHz, $CD_3OD+CDCl_3$) of **7** 69
Figure S69. ^{13}C NMR spectrum (125 MHz, $CD_3OD+CDCl_3$) of **7**........,,........70
Figure S70. DEPT-135 NMR spectrum (125 MHz, $CD_3OD+CDCl_3$) of **7**........71
Figure S71. HSQC spectrum (500 MHz, $CD_3OD+CDCl_3$) of **7**........ 72
Figure S72. HMBC spectrum (500 MHz, $CD_3OD+CDCl_3$) of **7**........ 73
Figure S73. NOESY spectrum (500 MHz, $CD_3OD+CDCl_3$) of 7 74
Figure S74. ^{1}H NMR spectrum (500 MHz, DMSO) of 8........75
Figure S75. ^{13}C NMR spectrum (125 MHz, DMSO) of **8**........76
Figure S76. ^{1}H NMR spectrum (700 MHz, DMSO) of **9**........77
Figure S77. ^{13}C NMR spectrum (176 MHz, DMSO) of **9**........78

Experimental Section

Table S1. NMR Data for compounds **1a** and **7**

Position	**1a**[a]			**7**[b]		
	^{13}C (δC)	^{1}H (δH, J in Hz)	HMBC	^{13}C (δ_C)	^{1}H (δ_H, *J* in Hz)	HMBC
1	36.1 CH_2	α: 2.06 d (13.5)<br>β: 2.20 dd (6.9, 12.5)	3, 6, 7, 13<br>2, 3, 6, 7, 8, 12	37.6 CH_2	α: 2.03 d (13.2)<br>β: 2.25 dd (8.1, 13.2)	2, 3, 6, 7, 13<br>2, 3, 6, 7, 8
2	84.7 CH	4.18 d (9.1)	3, 4, 7, 13	82.6 CH	4.25 m	3, 4, 7, 13
3	78.4 C			77.6 C		
4	33.2 CH_2	α: 1.49 m<br>β: 2.37 m	3, 5, 12<br>2, 3, 5, 6, 12	33.7 CH_2	α: 1.41 td (1.0, 13.1)<br>β: 2.41 dd (7.5,14.3)	3, 5, 6, 12<br>2, 3, 5, 6
5	23.1 CH_2	α: 2.44 d (7.6)<br>β: 1.80 m	4, 6, 7, 10<br>3, 4, 6, 7	18.1 CH_2	α: 1.50 m<br>β: 1.81 m	4, 6, 7, 10<br>3
6	55.8 CH	2.35 d (7.5)	1, 4, 5, 8, 13	56.8 CH	1.71 m	4, 5, 9, 10, 13
7	52.5 C			51.1 C		
8	34.2 CH_2	α: 1.71 dd (8.1, 12.5<br>β: 1.52 t (5.1)	1, 7, 9<br>6, 7, 9, 10, 13	32.0 CH_2	α: 1.85 dd (3.5, 10.5)<br>β: 1.68 m	1, 6, 7, 13<br>1, 6, 7, 9, 13
9	31.3 CH_2	α: 2.47 d (7.8)<br>β: 2.12 m	7, 10	38.5 CH_2	α: 2.12 m<br>β: 1.65 m	6, 7, 8, 10, 11<br>6, 7, 8, 11
10	140.0 C			84.5 C		
11	127.9 C			148.8 C		
12	175.9 C			176.7 C		
13	75.9 CH_2	a: 3,38 dd (1.4, 8.5)<br>b: 3.77 d (8)	3, 6, 7, 8<br>1, 2, 6, 7	75.0 CH_2	a: 3.41 d (8.0)<br>b: 4.67 d (8.1)	6, 7, 8<br>1, 6, 7, 8
14	66.1 CH_2	a: 4.00 d (11.1)<br>b: 4.09 d (11.0)	10, 11, 14	18.9 CH_3	1.78s	10, 11, 15
15	16.6 CH_3	1.84 brs	10, 11, 15	109.3 CH_2	a: 4.87 t (1.5)<br>b: 5.01 brs	10, 11, 14
16	52.5 CH_3	3.79 s	12	37.6 CH_2		

[a] Chemical shifts were measured at 500.13 Hz and 125.77 Hz in CD_3OD.[b] Chemical shifts were measured at 500.13 Hz and 125.77 Hz in mixture CD_3OD+$CDCl_3$.

Table S2. NMR data for piltunines A–C (**1–3**) [a].

Position	**1**				**2**				**3**			
	^{13}C (δC)	^{1}H (δH, J in Hz)	HMBC	NOESY	^{13}C (δ_C)	^{1}H (δ_H, *J* in Hz)	HMBC	NOESY	^{13}C (δC)	^{1}H (δH, J in Hz)	HMBC	NOESY
1	37.4 CH_2	α: 2.05 d (13.1)<br>β: 2.19 dd (8.6, 13.3)	2, 6, 7, 13<br>2, 3, 6, 7, 8, 12	6<br>2, 8β, 13a	37.3 CH_2	α: 2.05 d (14.1)<br>β: 2.19 d (8.2)	3, 6, 7, 13<br>2, 3, 6, 7, 8	4α, 6, 8α<br>2, 8β, 13a	34.3 CH_2	α: 1.89 d (13.0)<br>β: 2.28 dd (8.7, 13.0)	3, 6, 7, 13<br>2, 3, 7, 8	4α, 6, 8α<br>2, 8β
2	85.5 CH	4.32 d (8.5)	1, 3, 4, 12, 13	1β	85.5 CH	4.32 d (8.1)	3, 4, 7, 13	1β	84.1 CH	4.40 d (8.5)	3, 4, 7, 13	1β
3	80.0 C				79.9 C				78.9 C			
4	35.7 CH_2	α: 1.43 t (12.0)<br>β: 2.44 m	3, 5, 6<br>2, 3, 5, 6, 12	6	35.6 CH_2	α: 1.43 t (11.0)<br>β: 2.44 m	 2, 5, 6, 7	1α, 6<br>15	35.6 CH	α: 1.42 t (12.3)<br>β: 2.43 m	3, 6<br>2, 3, 6	1α, 6<br>15
5	25.0 CH_2	2.44 m	3, 4, 6, 7<br>3, 7	15<br>13b	25.0 CH_2	α: 2.43 m<br>β: 1.73 m	3, 6, 7<br>7	15<br>13b	25.3 CH_2	α: 2.40 m<br>β: 1.71 dd (14.0, 12.6)	3, 4<br>3, 7	13<br>15
6	57.6 CH	2.37 brd (11.6)	5, 9	1α, 4α, 8α, 15	57.7 CH	2.39 d (12.0)		1α, 4α, 8α, 15	58.1 CH	2.31 d (14.0)		4
7	54.4 C				54.5 C				58.5 C			
8	35.9 CH_2	α: 1.71 m<br>β: 1.53 dd (7.2, 12.1)	1, 6, 7, 9, 13<br>5, 6, 7, 9, 10, 13		35.7 CH_2	α: 1.74 m<br>β: 1.54 dd (8.1, 12.0)	7, 9<br>6, 7, 9, 10, 13	4, 6, 13	31.9 CH_2	α: 1.46 dd (3.1, 12.2)<br>β: 1.94 dd (7.1, 12.2)	1, 7, 9, 13<br>6, 10, 13	1α, 6<br>1β
9	32.6 CH_2	α: 2.47 m<br>β: 2.11 m	6, 7, 8, 10, 11	14a,b	32.8 CH_2	α: 2.49 dd (9.1, 17.2)<br>β: 2.17 m	6, 7, 8<br>7, 10	14a,b<br>13b, 14a,b	32.6 CH_2	α: 2.24 m<br>β: 2.21 m		14<br>13, 14
10	141.3 C				144.5 C				137.4 C			
11	129.7 C				125.2 C				124.8 C			
12	178.4 C				178.4 C				178.4 C			
13	77.0 CH_2	a: 3.37 dd (1.4, 8.3)<br>b: 3.73 d (8.1)	6, 7, 8<br>1, 2, 6, 7	1β, 8β, 9β<br>5β, 9β	77.0 CH_2	a: 3.38 dd (1.0, 8.1)<br>b: 3.71 d (8.1)	6, 8<br>1, 2, 6, 7	1β, 8β<br>5β, 9β	101.1 CH	4.74 s	1, 2, 6, 7	5β, 9β
14	66.5 CH_2	a: 3.92 d (11.8)<br>b: 4.01 d (11.8)	10, 11, 15<br>10, 11, 15	9α<br>9α	69.4 CH_2	a: 4.48 d (12.0)<br>b: 4.52 d (12.1)	10, 11, 15, 16<br>10, 11, 15, 16	9α,β, 15<br>9α,β, 15	24.3 CH_3	1.62 s	10, 11, 15	9α,β, 15
15	17.3 CH_3	1.81 brs	10, 11 14	5α, 6	17.5 CH_3	1.78 drs	10, 11, 14	4β, 5α, 6	21.4 CH_3	1.74 brs	10, 11, 14	4β, 5α, 6, 14
16					173.6 C							
17					21.4 CH_3	2.03 s	14, 16	9β, 15				

[a] Chemical shifts were measured at 700.13 Hz and 176.04 Hz in CD_3OD.

Table S3. NMR data for piltunines D–F (**4**–**6**) [a]

Position	**4**				**5**				**6**			
	^{13}C (δC)	^{1}H (δH, J in Hz)	HMBC	NOESY	^{13}C (δ_C)	^{1}H (δ_H, *J* in Hz)	HMBC	NOESY	^{13}C (δC)	^{1}H (δH, J in Hz)	HMBC	NOESY
1	36.9 CH_2	α: 2.16 d (13.1)<br>β: 2.21 dd (8.1, 13.0)	6, 7, 13<br>2, 3, 6, 7, 8	4α, 6<br>2, 8β	36.3 CH_2	α: 2.19 d (13.5)<br>β: 2.24 d (8.8)	2, 3, 6, 7, 13<br>2, 3, 6, 7, 13	13a<br>4α, 6, 8α	39.1 CH_2	α: 2.32 d (13.4)<br>β: 2.07 dd (8.3, 13.4)	3, 6, 7, 13<br>3, 3, 6, 7, 8	4α<br>8α, 13a
2	85.6 CH	4.33 d (8.5)	1, 3, 7	1β	85.7 CH	4.36 d (8.4)	1, 3, 4, 7, 12, 13	1α,β	85.0 CH	4.42 d (8.4)	4, 7, 12, 13	1α,β
3	80.0 C				80.0 C				78.6 C			
4	36.7 CH_2	α: 1.37 m<br>β: 2.46 m	6<br>2, 3, 12	6	36.4 CH_2	α: 1.39 t (14.0)<br>β: 2.48 dd (8.0, 14.0)	3, 5, 6, 12<br>2, 3, 5, 6, 12	1α	32.6 CH_2	α: 1.46 ddd (14.7, 10.4, 1.5)<br>β: 2.26 dd (10.3, 14.6)	2, 3, 5, 6<br>3, 5, 6, 12	1α<br>13b
5	23.5 CH_2	α: 2.37 dd (7.9, 14.5)<br>β: 1.88 q (14.5)	3	14,15<br>13b	21.8 CH_2	α: 2.61 m<br>β: 1.64 q (13.0)	6, 7, 10<br>3, 4, 6, 7	13b	23.3 CH_2	α: 3.12 ddd (15.6, 9.8, 1.6)<br>β: 2.74 ddd (15.9, 10.4, 1.7)	3, 6<br>3	13b
6	60.4 CH	2.59 d (12.0)		1α, 4α, 14, 15	58.7 CH	2.70 d (13.0)	1, 7, 9	1α, 8α	162.7 C			
7	56.0 C				55.5 C				60.5 C			
8	40.4 CH_2	α: 2.48 m<br>β: 2.06 dd (2.8, 15.6)	1, 10<br>6, 7, 9, 10, 13	1α<br>1β, 13a	40.9 CH_2	α: 2.26 dd (2.6, 14.0)<br>β: 2.62 m	1, 6, 7, 9, 10, 13<br>1, 6, 7, 9, 10, 11, 13	1α	33.4 CH_2	α: 1.96 m<br>β: 1.89 m	1, 6, 7, 9, 10, 13<br>1, 6, 7, 9, 10, 13	1β<br>13a
9	129.7 CH	5.68 dd (2.8, 5.0)	6, 7, 10, 11	14, 15, 16	147.8 CH	6.90 q (2.6)	6, 7, 8, 10, 11	14	33.7 CH_2	2.67 m	6, 10	14
10	152.0 C				149.0				135.4 C			
11	77.7 C				200.8 C				202.5 C			
12	178.6 C				178.5 C				178.3 C			
13	78.5 CH_2	a: 3.38 d (8.5)<br>b: 3.98 d (8.4)	6, 7, 8<br>1, 2, 6, 7	1β, 8β<br>5β	77.6 CH_2	a: 3.38 d (8.7)<br>b: 3.91 d (8.4)	6, 7, 8<br>1, 2, 6, 7	1β<br>5β	79.7 CH_2	a: 3.74 d(8.8)<br>b: 3.84 d (8.4)	1, 2, 6, 7<br>6, 7, 8	1β, 8β<br>4β, 5β
14	27.4 CH_3	1.30 s	10, 11,15	5α, 6, 9, 16	27.9 CH_3	2.26 s	9, 10, 11	9	31.0 CH_3	2.24 s	9,10, 11	9
15	26.6 CH_3	1.31 s	10, 11, 14	5α, 9, 16								
16	51.8 CH_3	3.12 s	11	5α, 6, 9, 13b, 14, 15								

[a] Chemical shifts were measured at 700.13 Hz and 176.04 Hz in CD_3OD.

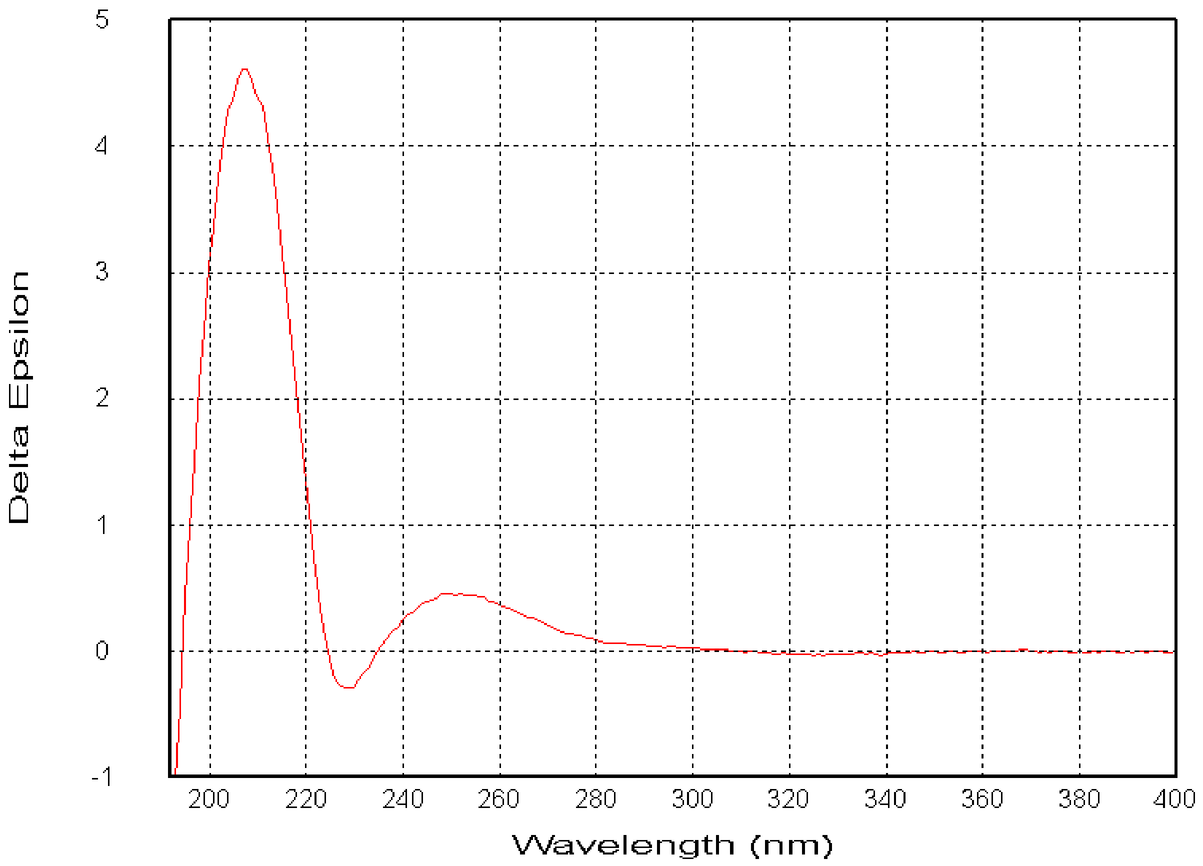

Figure S1. CD spectrum of **1**

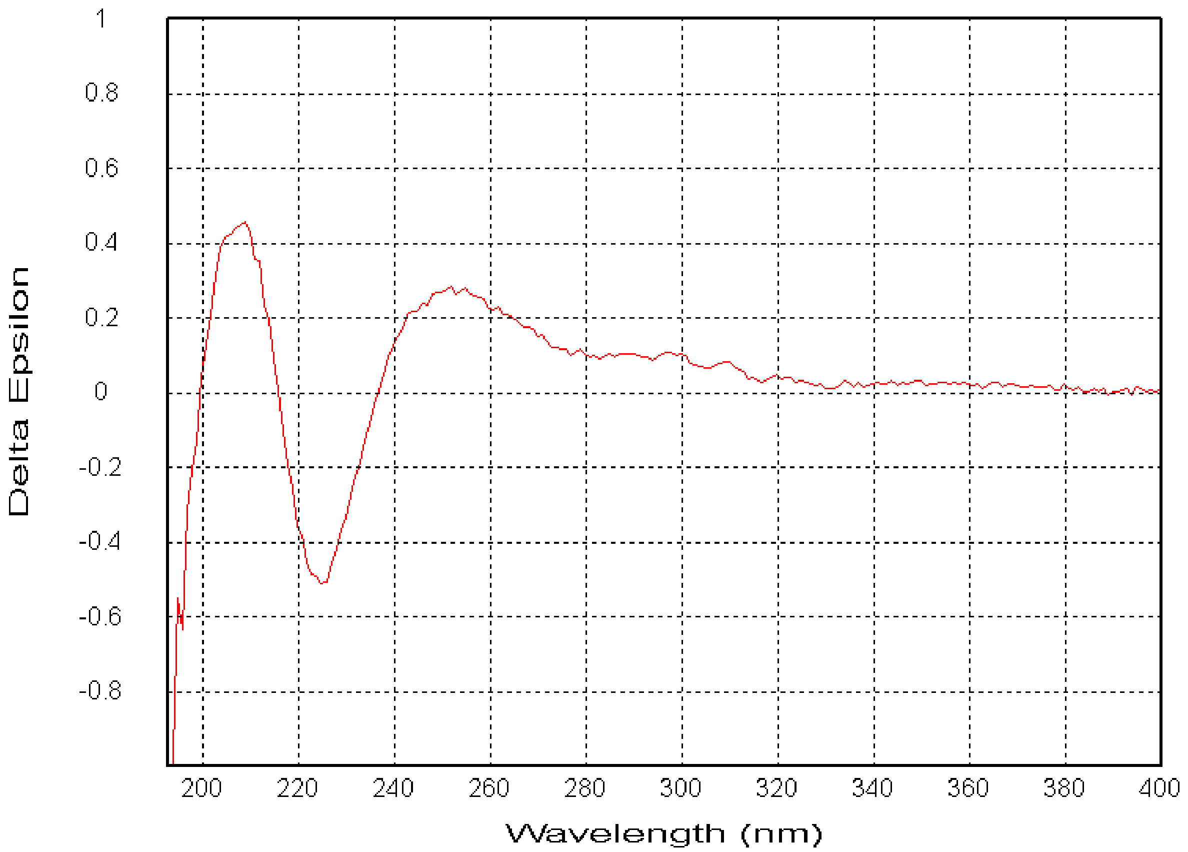

Figure S2. CD spectrum of **2**

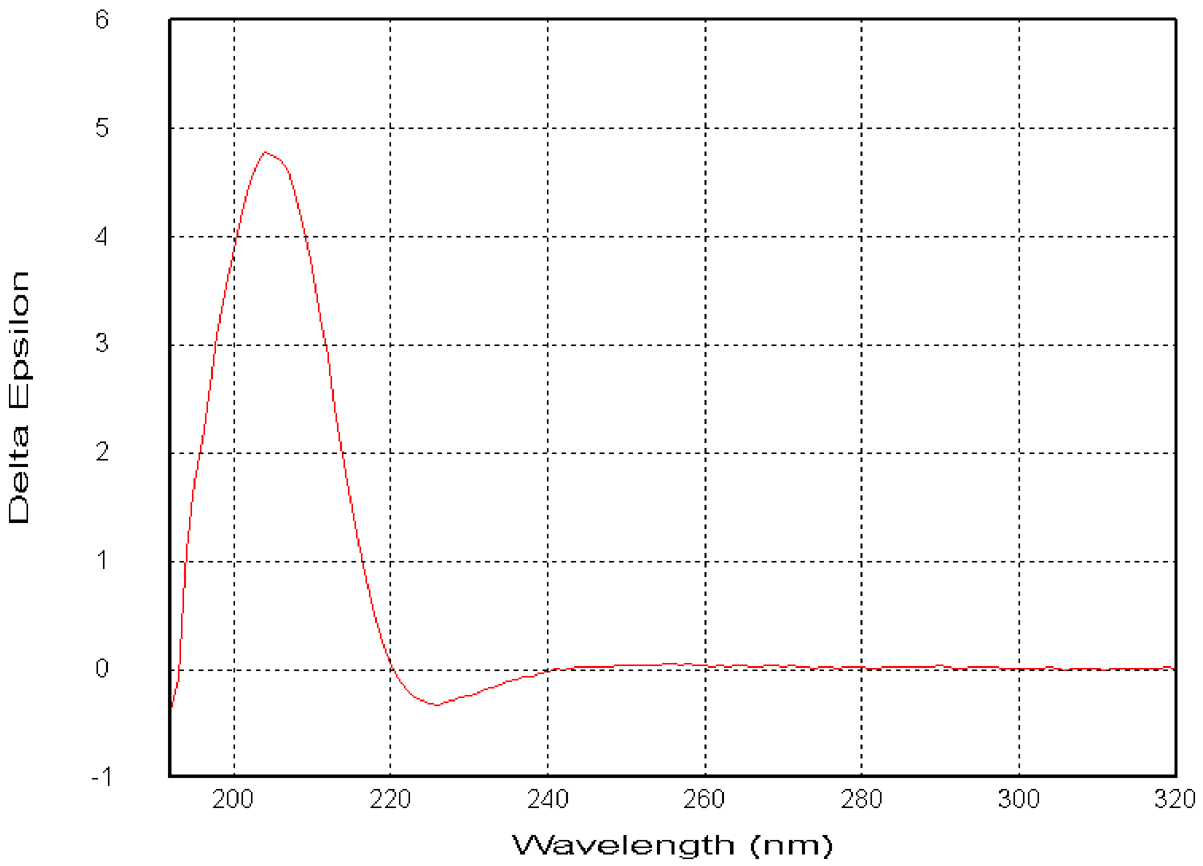

Figure S3. CD spectrum of **3**

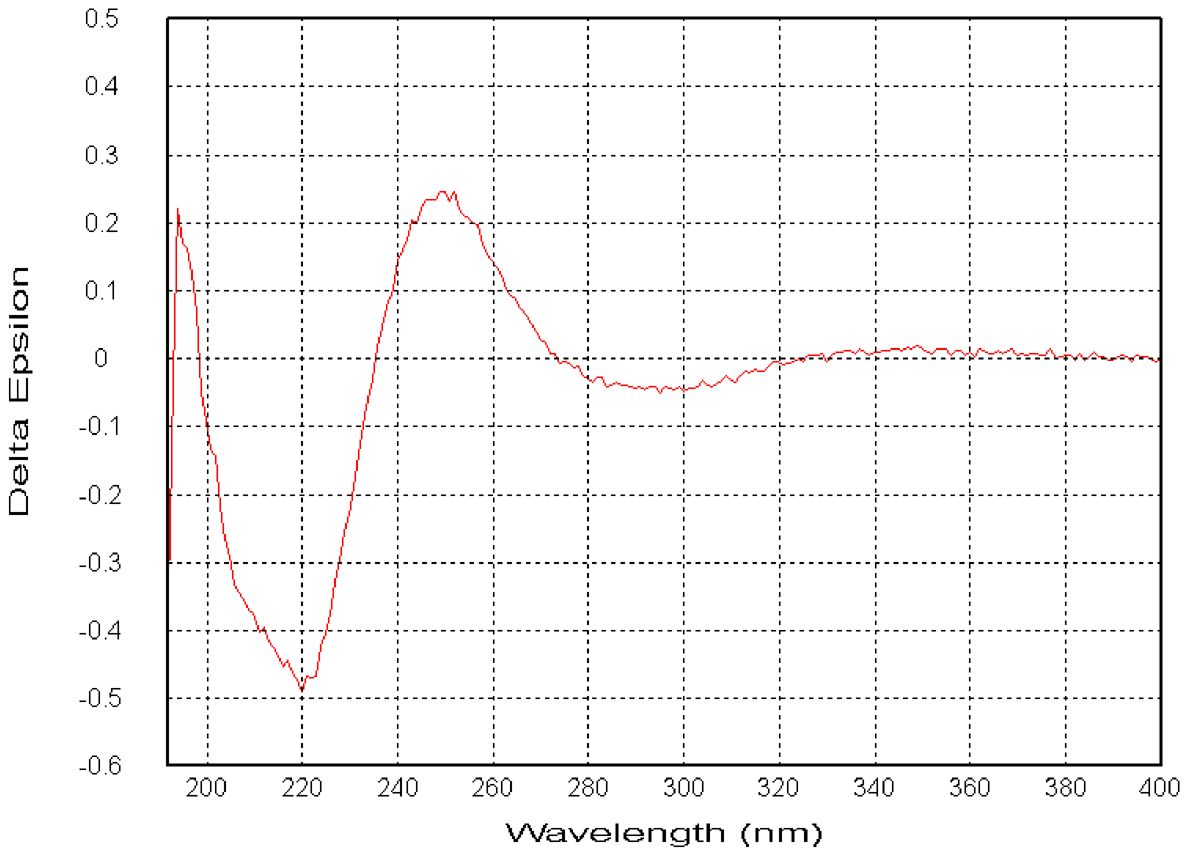

Figure S4. CD spectrum of **4**

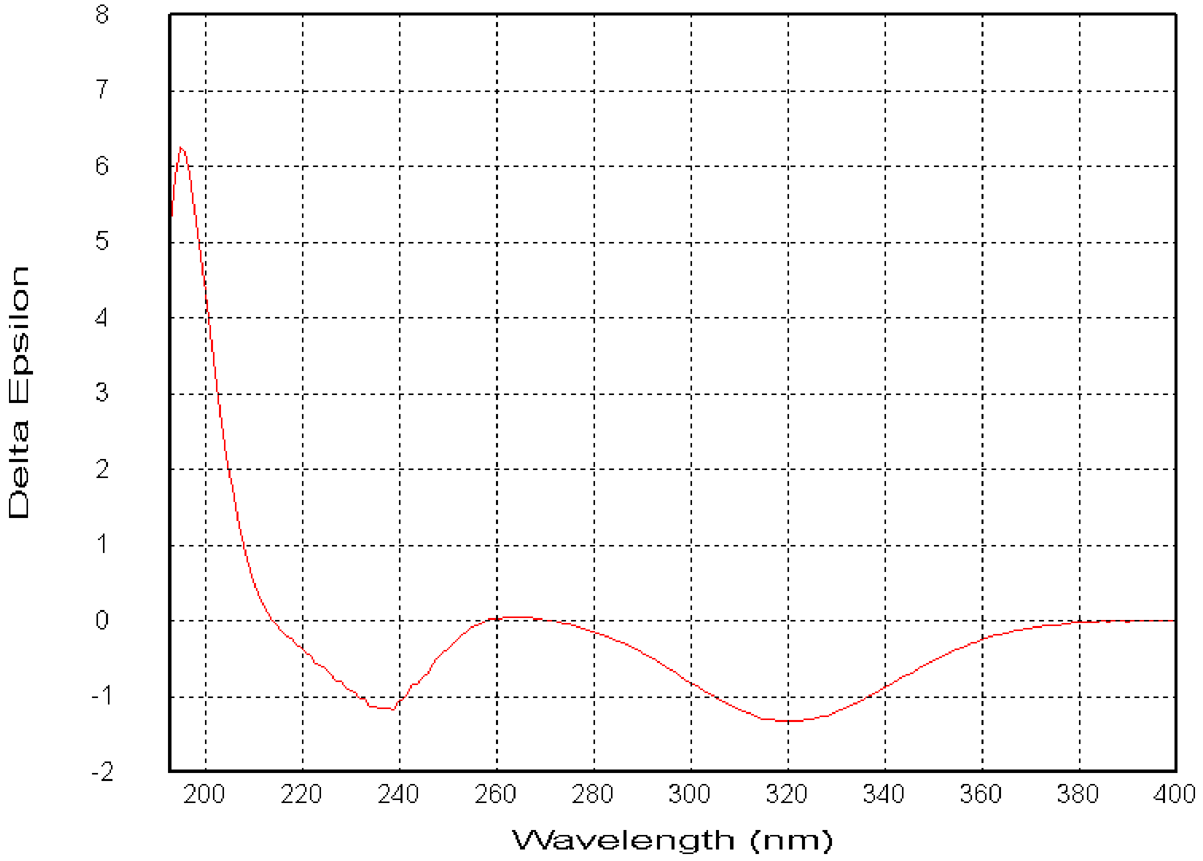

Figure S5. CD spectrum of **5**

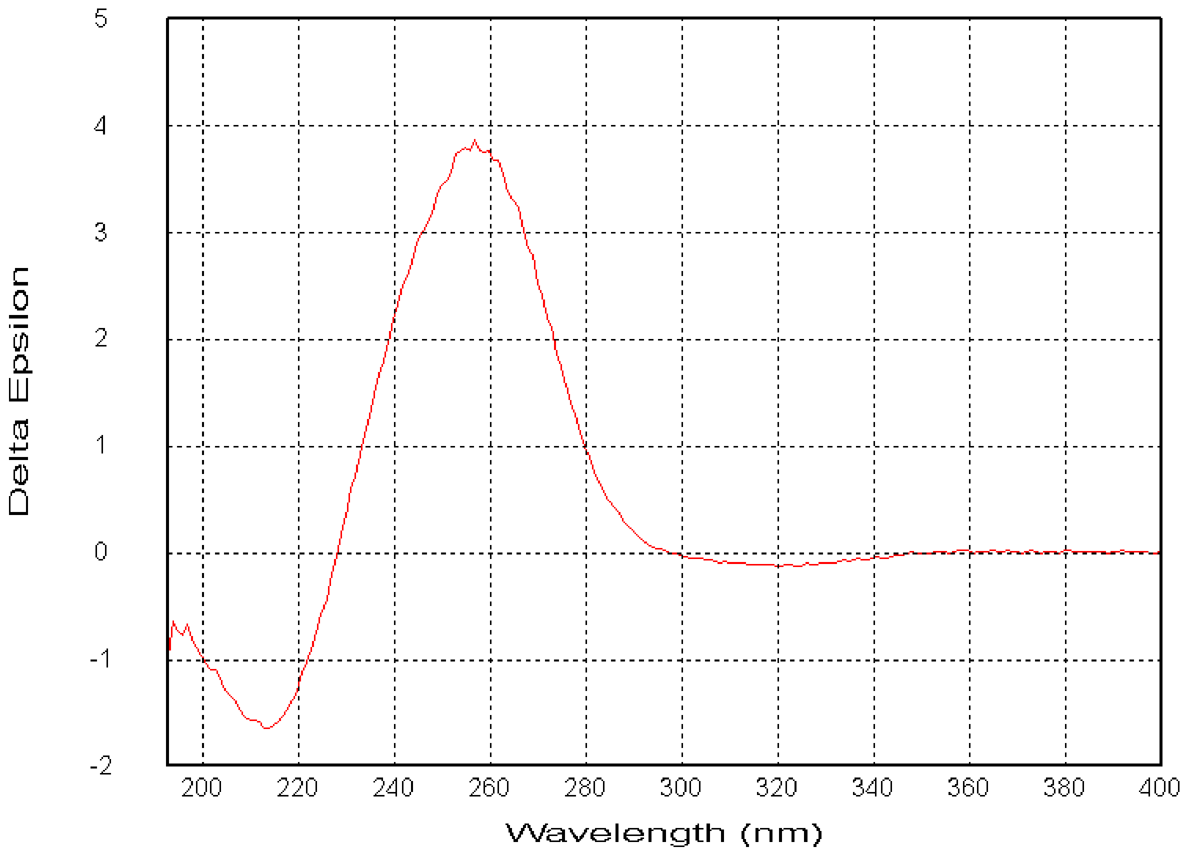

Figure S6. CD spectrum of **6**

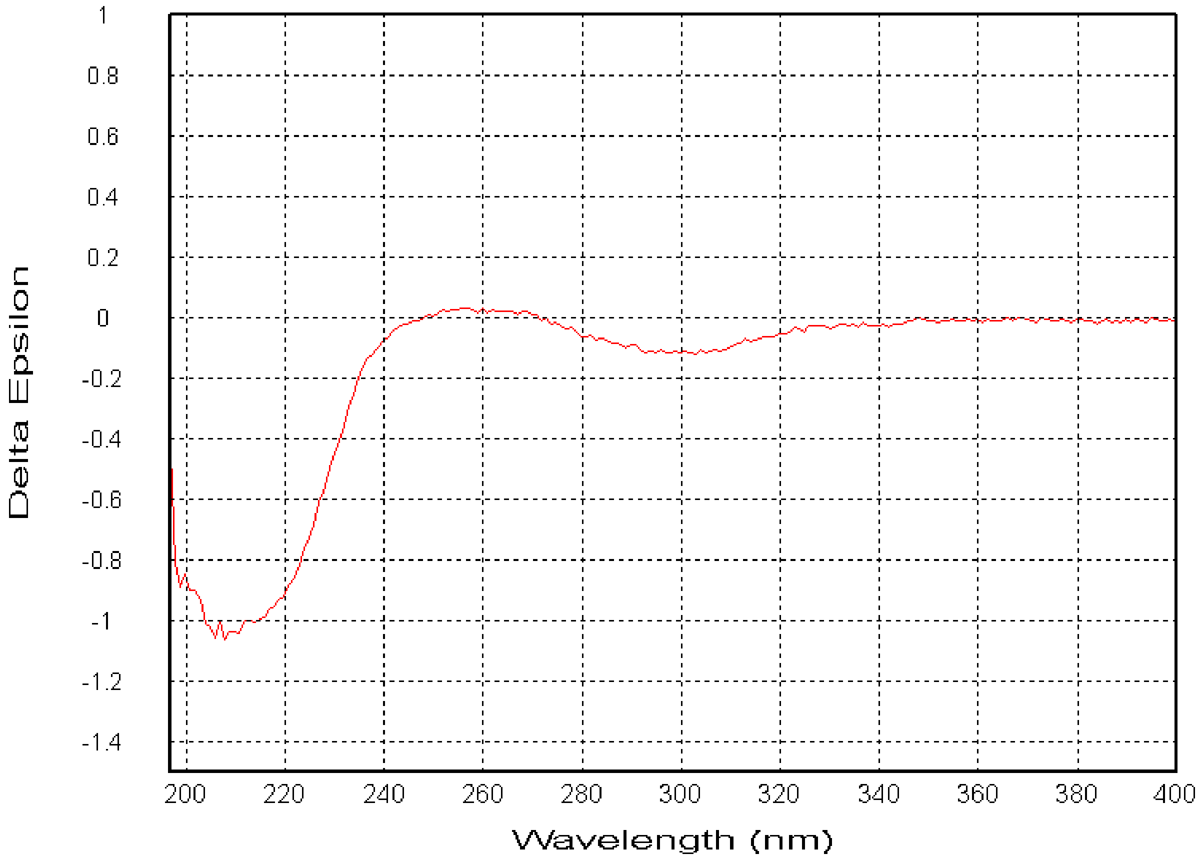

Figure S7. CD spectrum of **7**

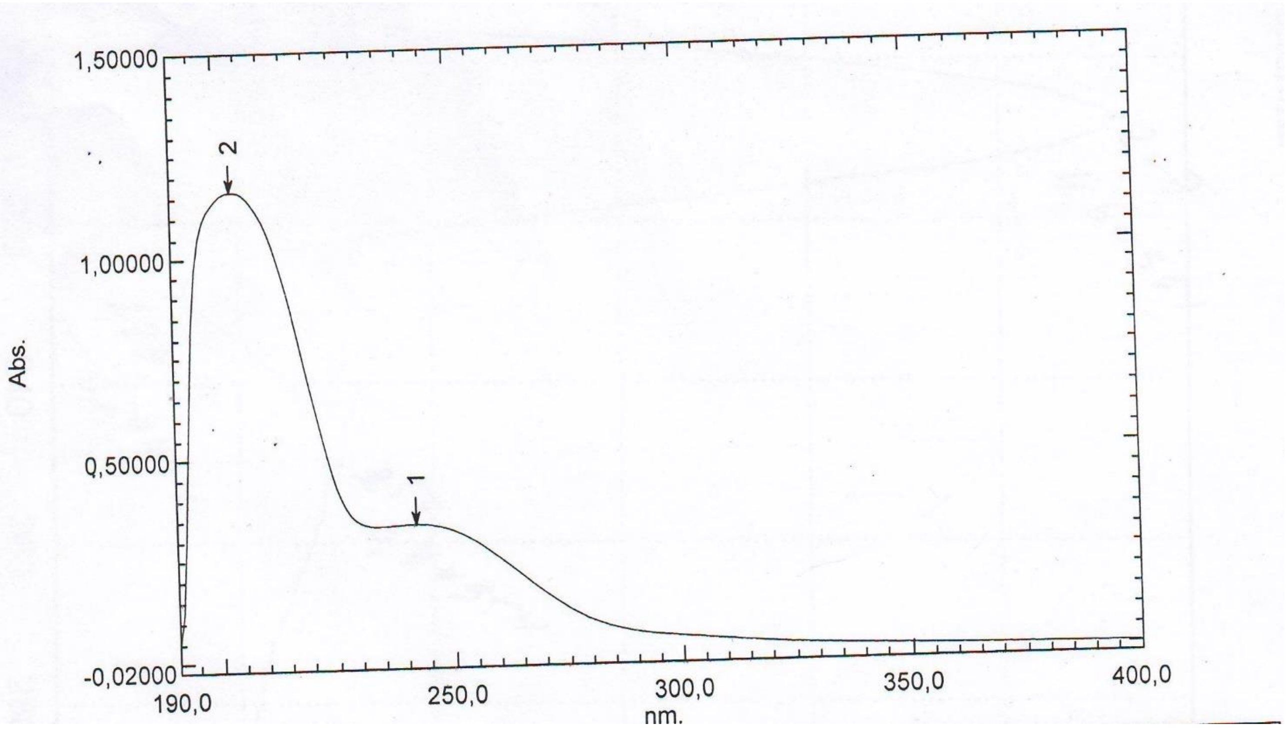

Figure S8. UV spectrum of **1**

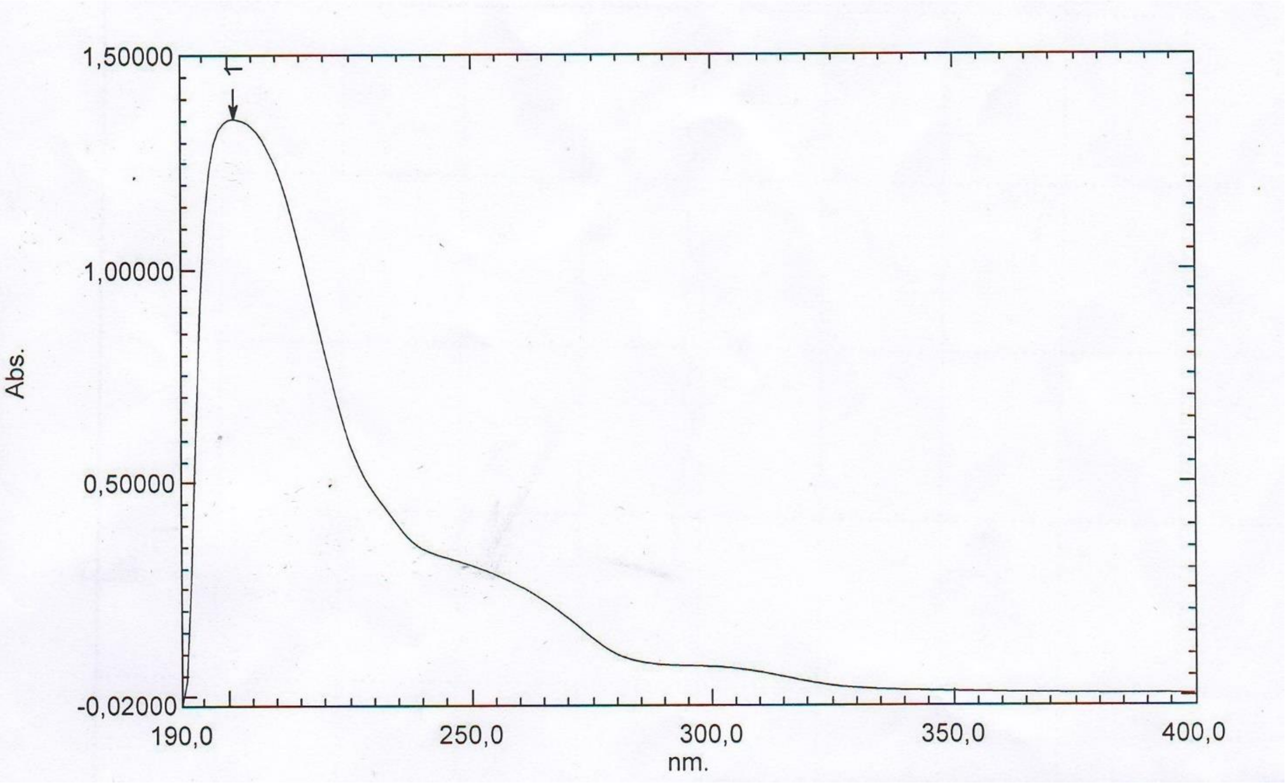

Figure S9. UV spectrum of **2**.

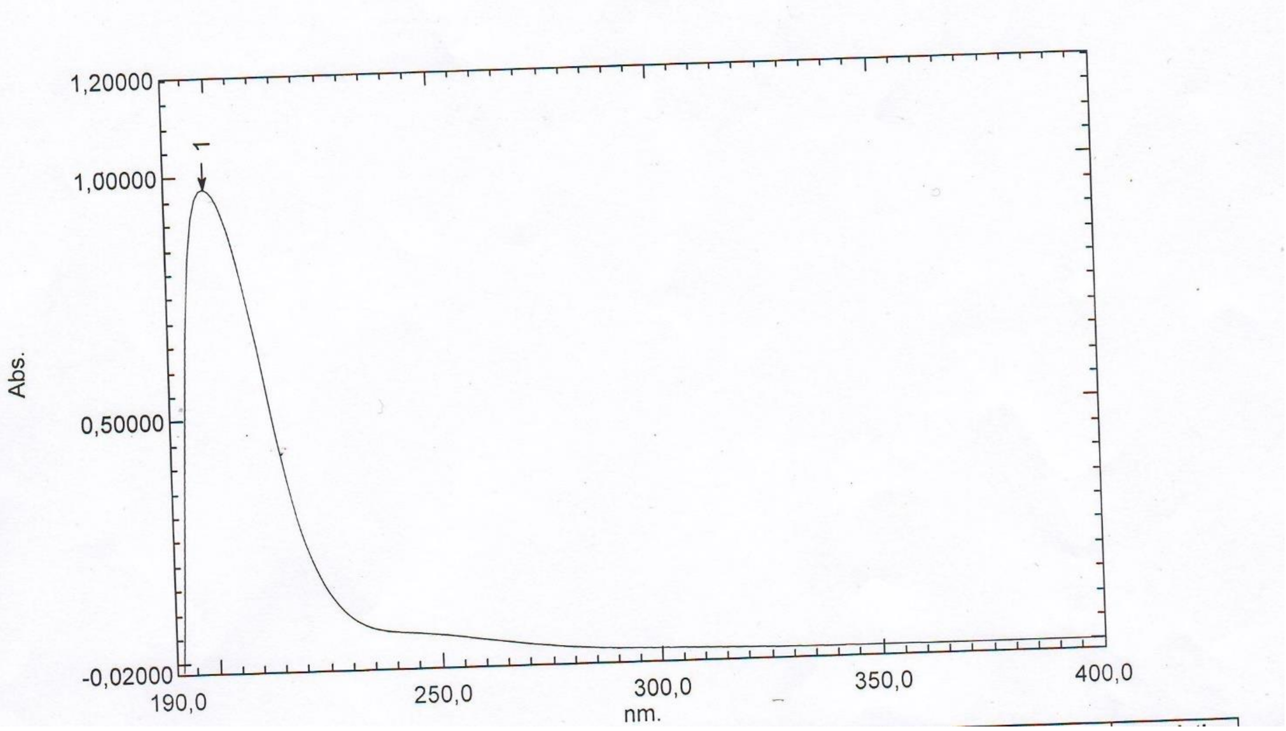

Figure S10. UV spectrum of **3**

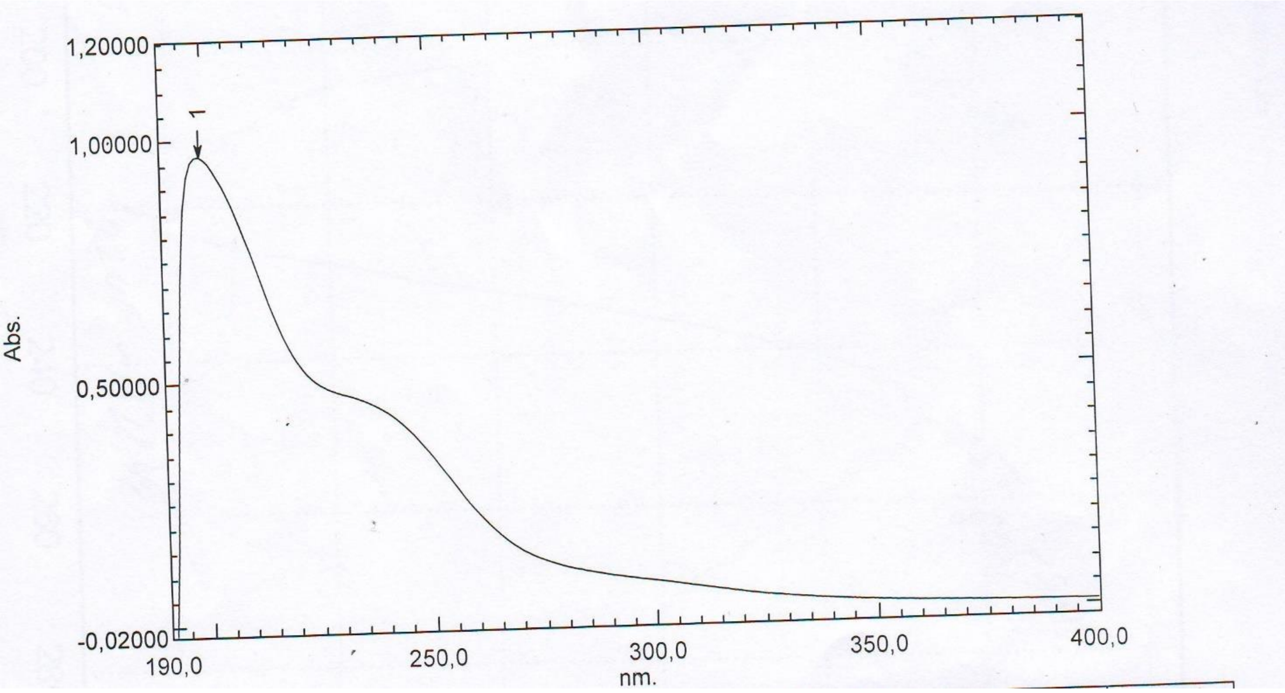

Figure S11. UV spectrum of **4**

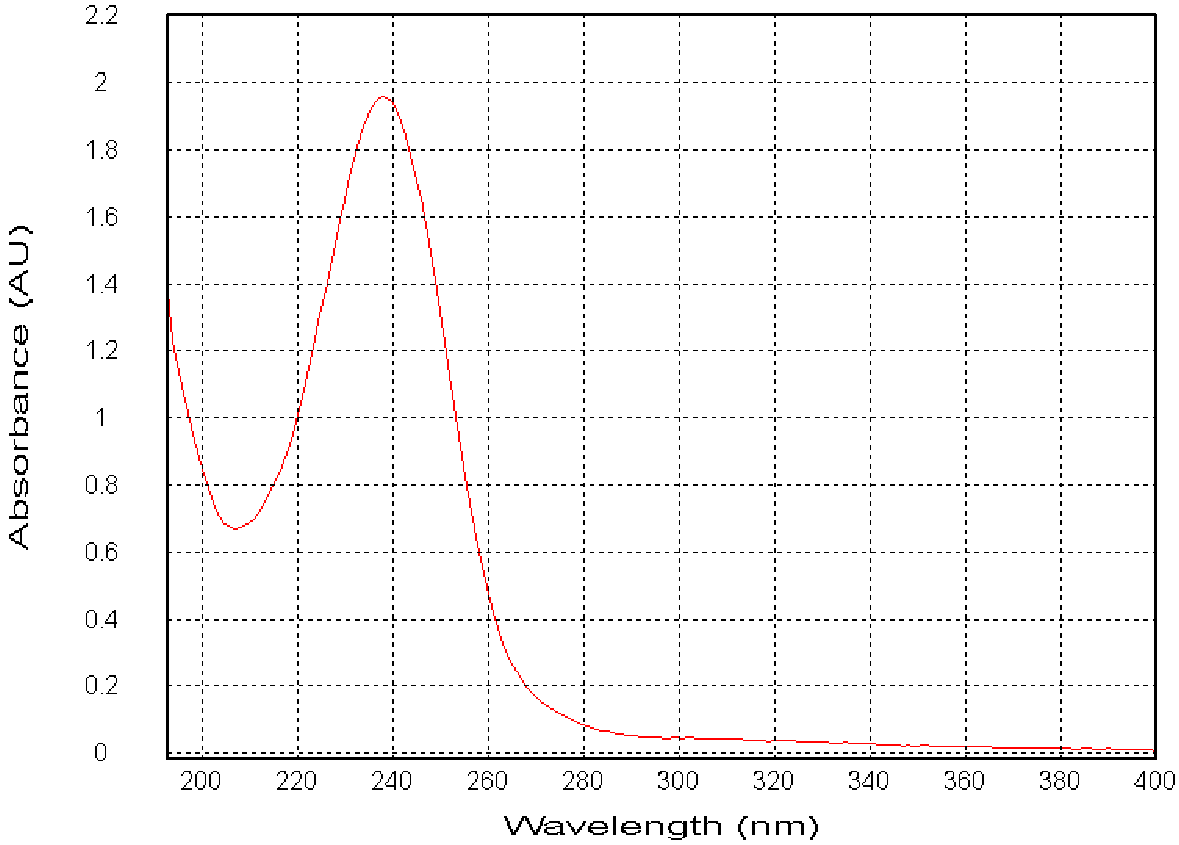

Figure S12. UV spectrum of **5**

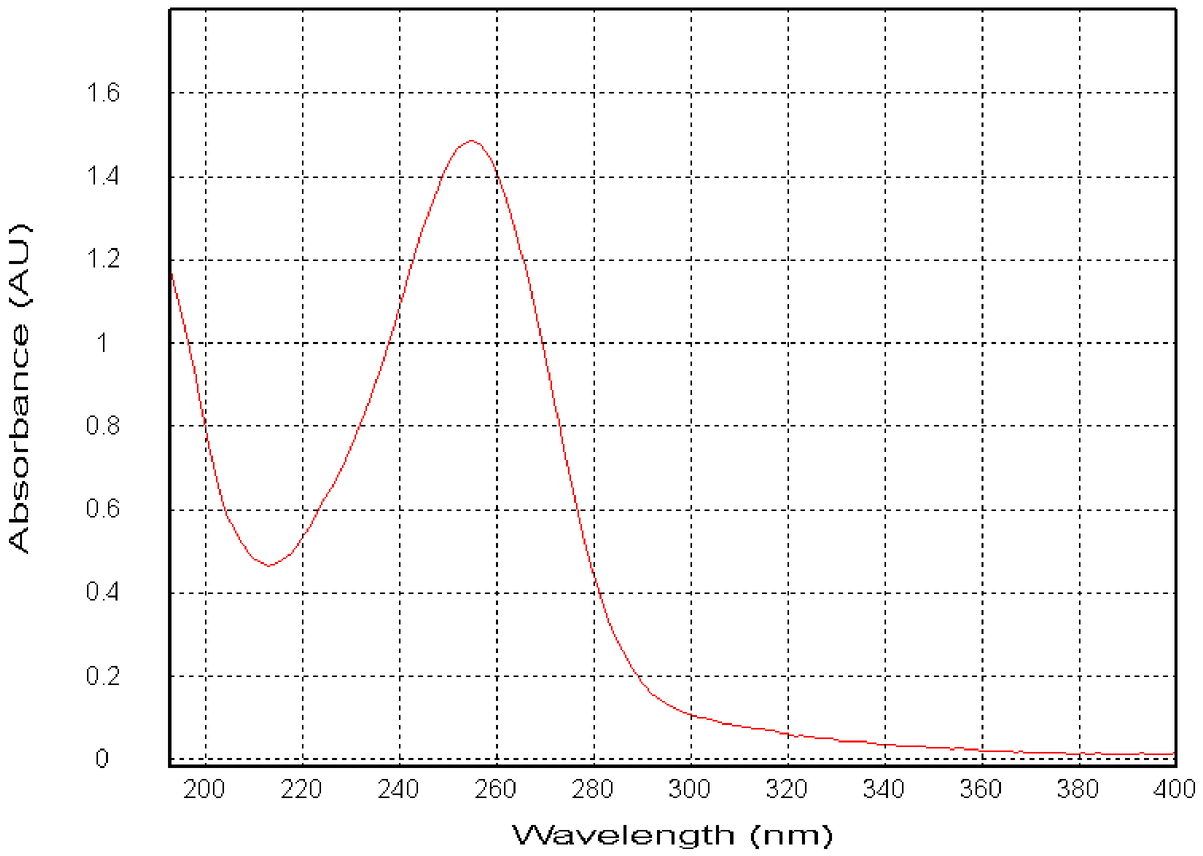

Figure S13. UV spectrum of **6**

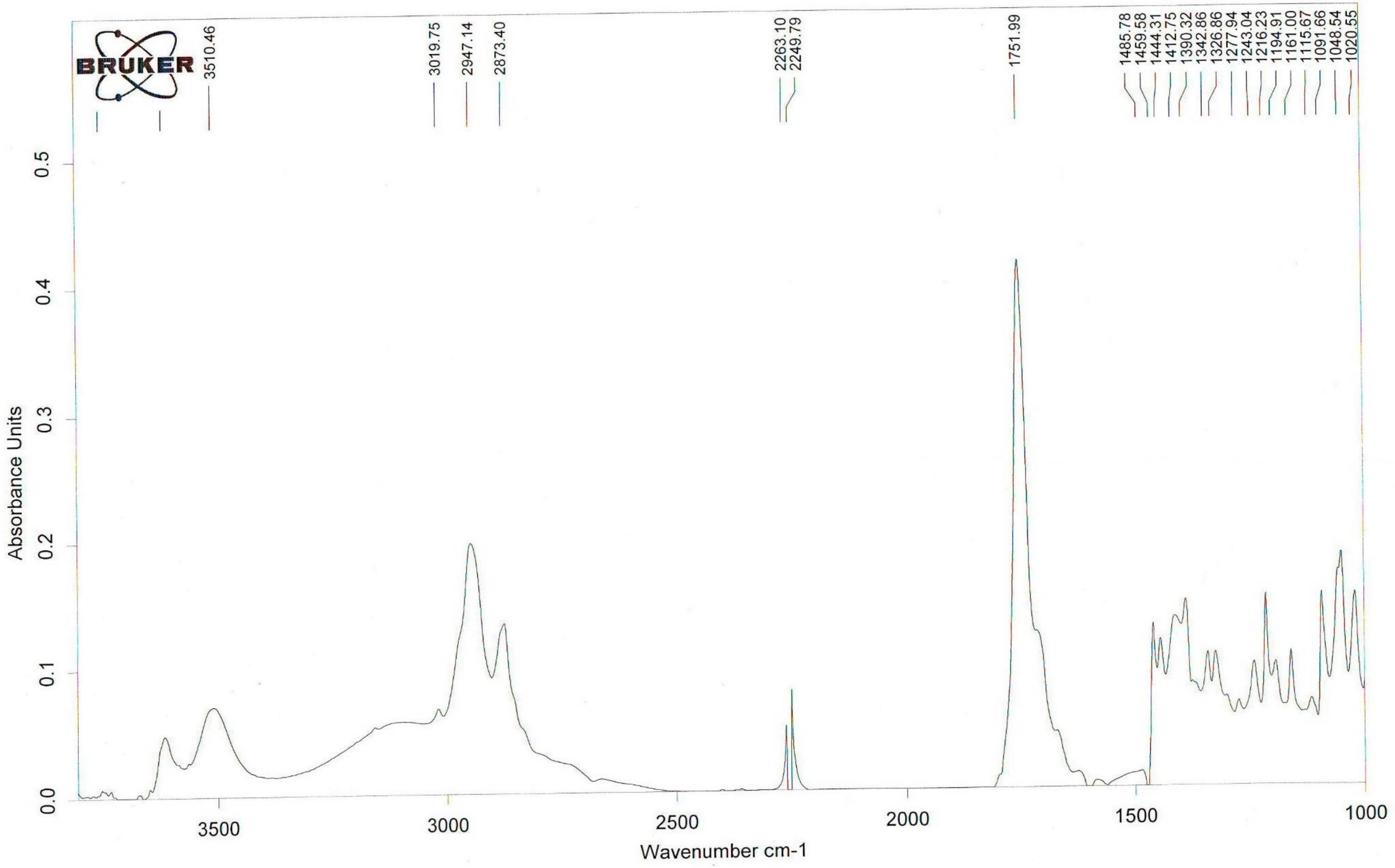

Figure S14. IR spectrum of **1** (3500 – 1000 cm^{-1})

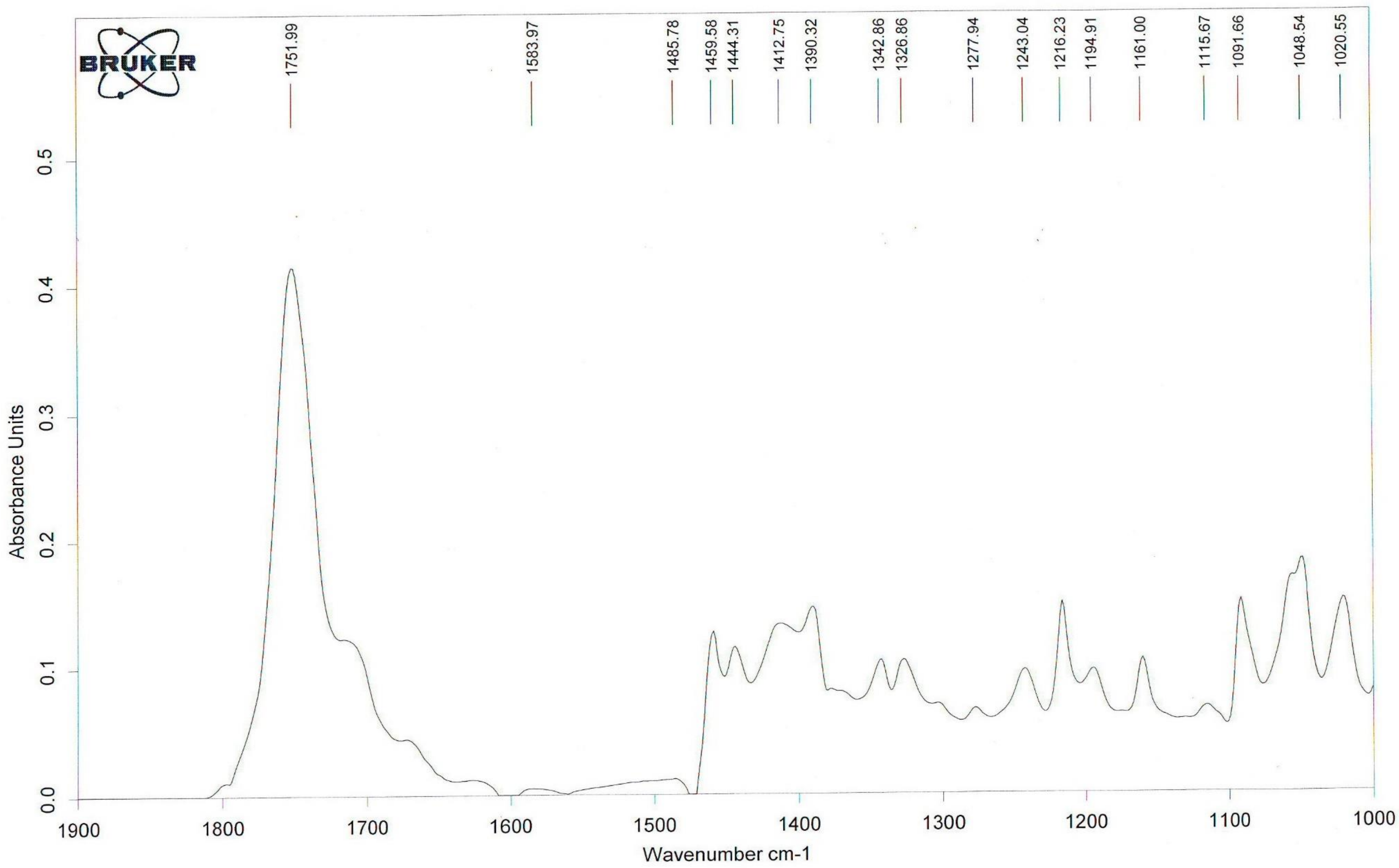

Figure S15. IR spectrum of **1** (1900 – 1000 cm^{-1})

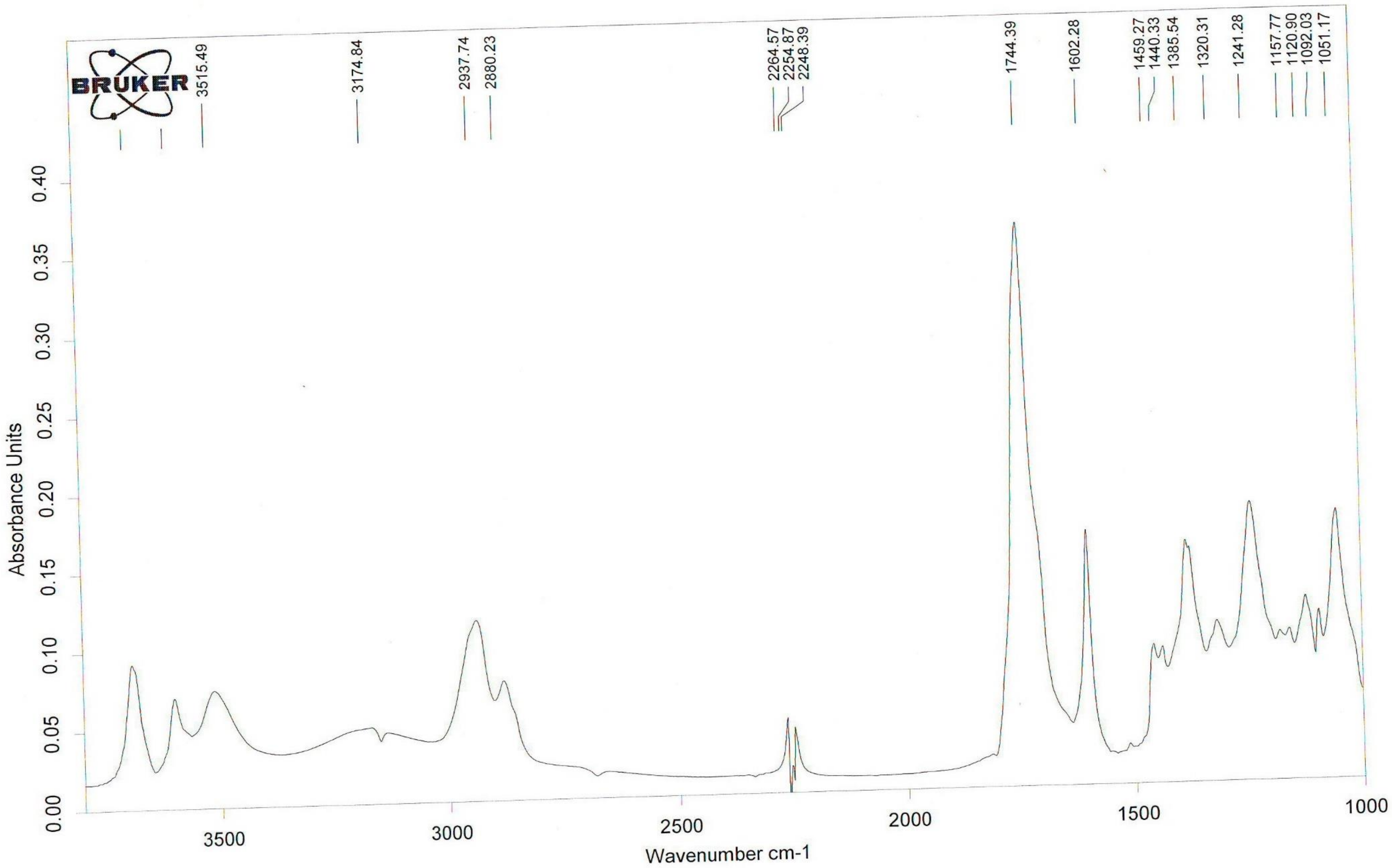

Figure S16. IR spectrum of **2** (3500 – 1000 cm^{-1})

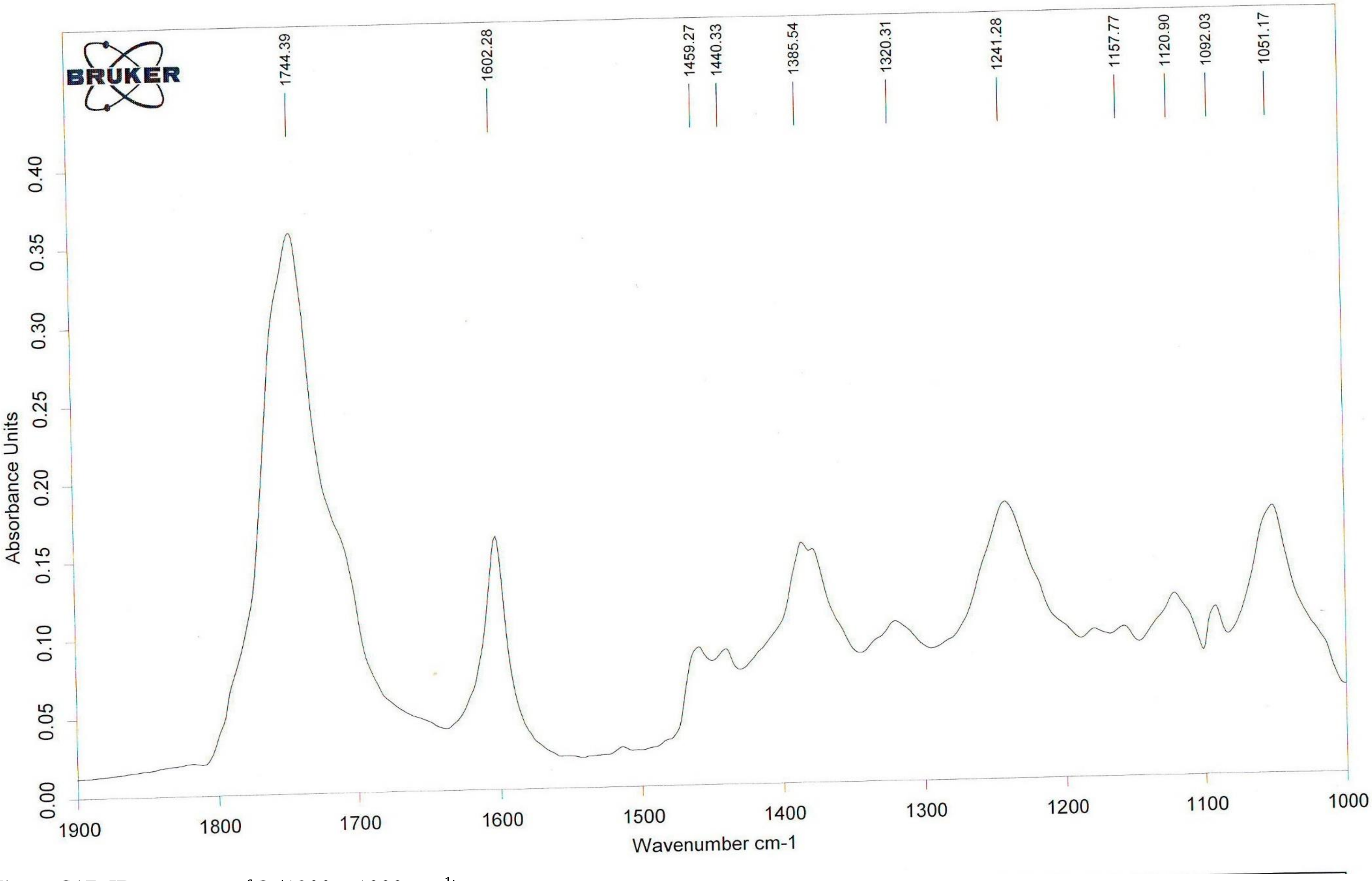

Figure S17. IR spectrum of **2** (1900 – 1000 cm^{-1})

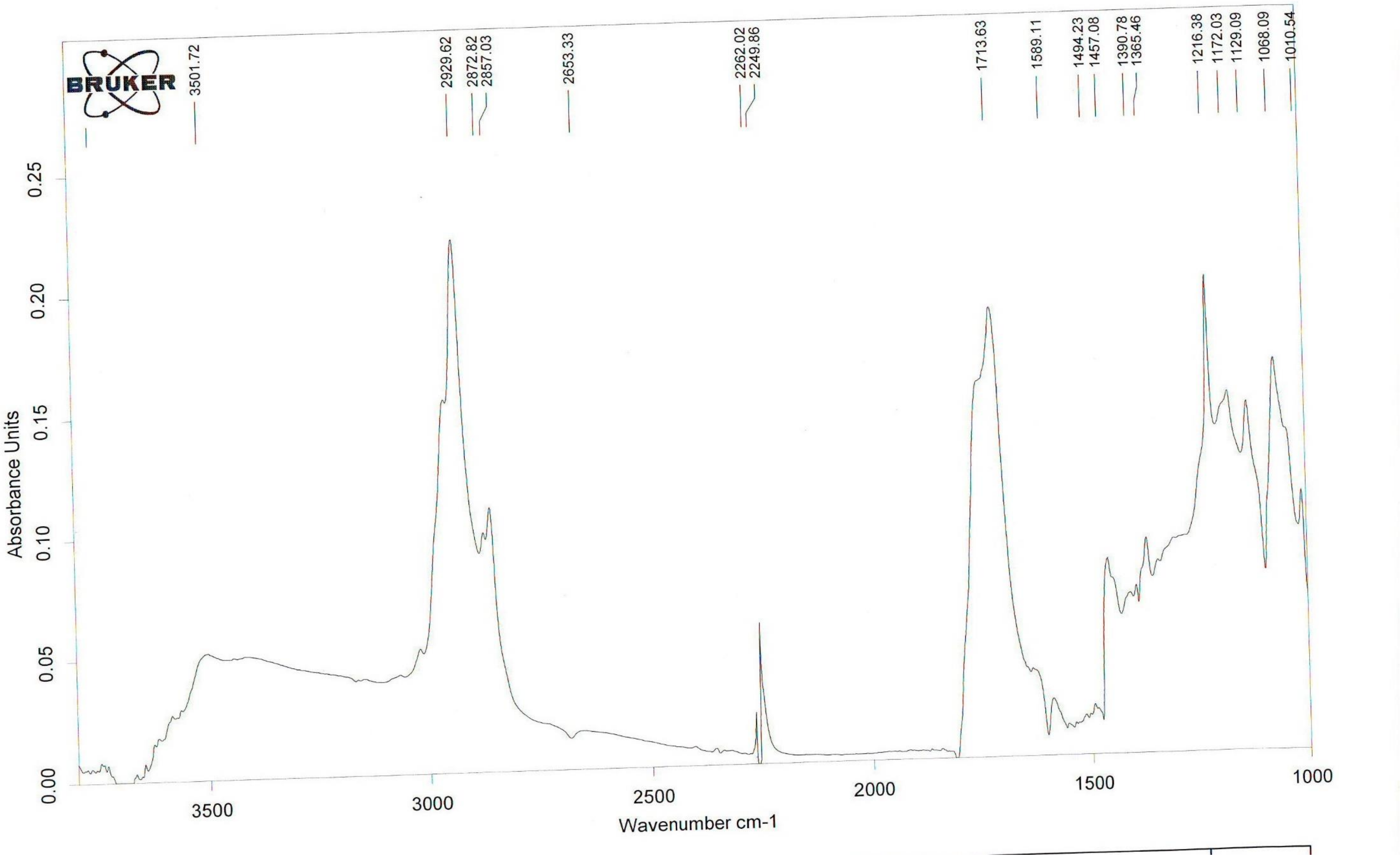

Figure S18. IR spectrum of **4** (3500 – 1000 cm^{-1})

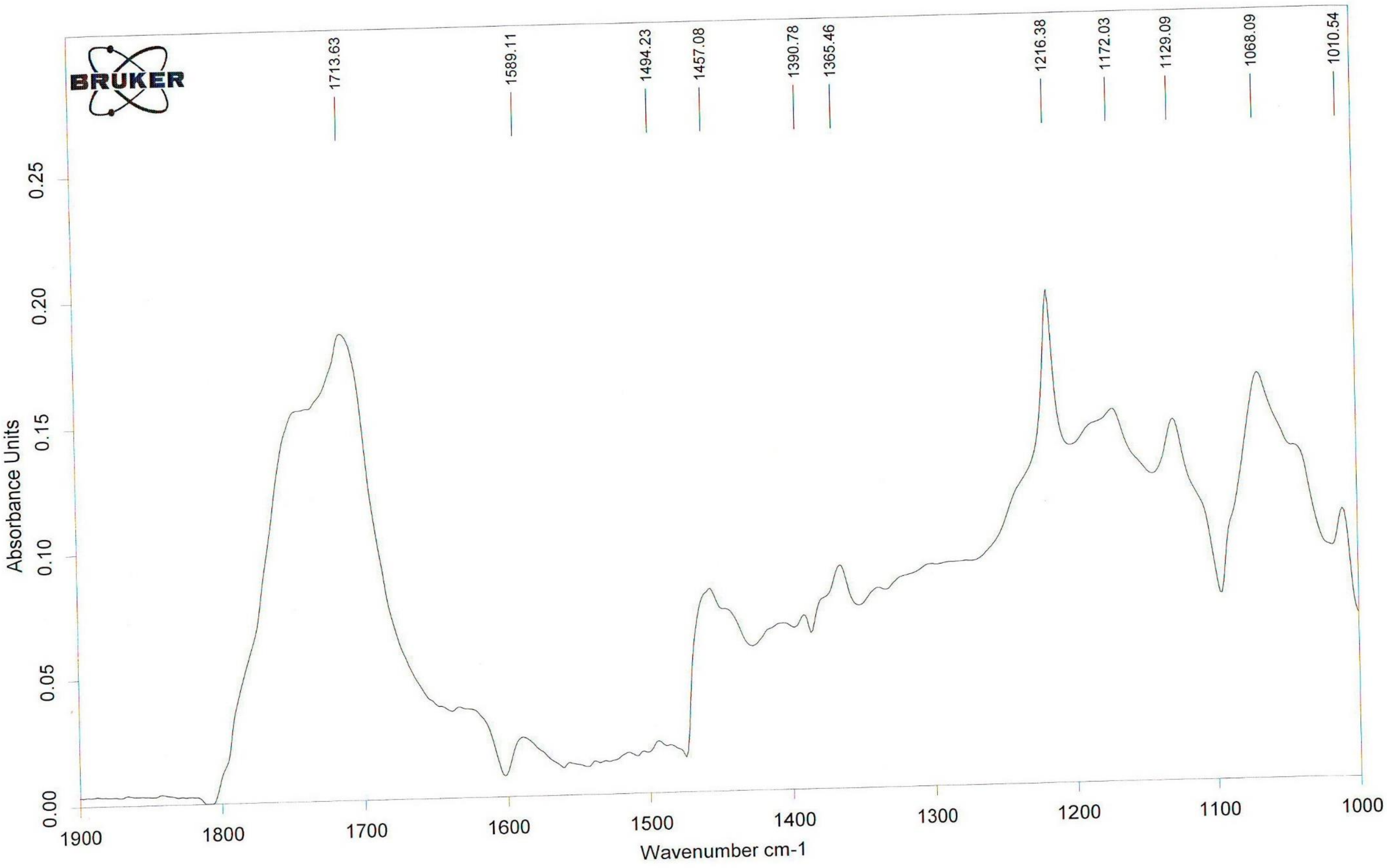

Figure S19. IR spectrum of **4** (1900 – 1000 cm^{-1})

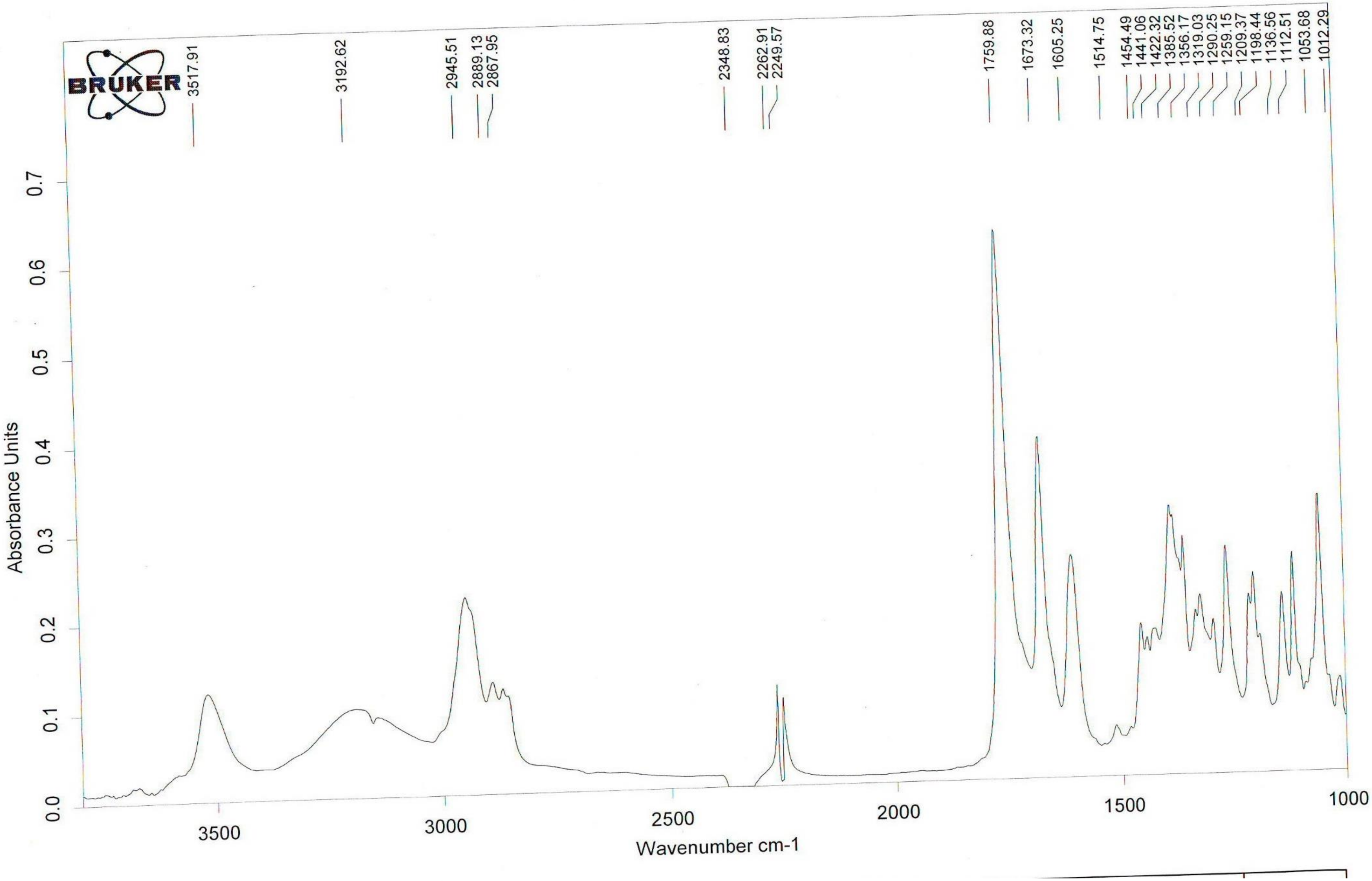

Figure S20. IR spectrum of **6** (3500 – 1000 cm^{-1})

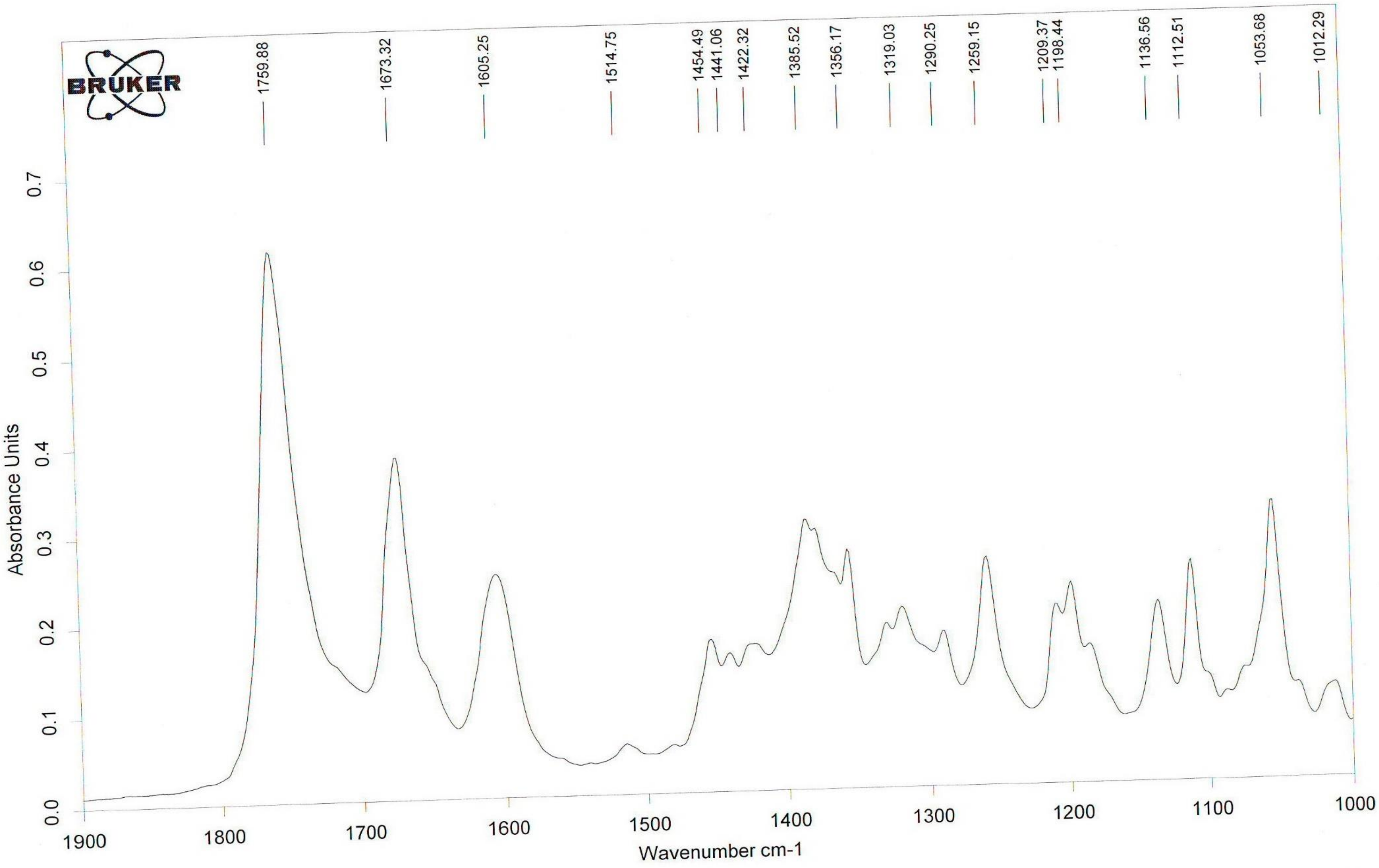

Figure S21. IR spectrum of **6** (1900 – 1000 cm^{-1})

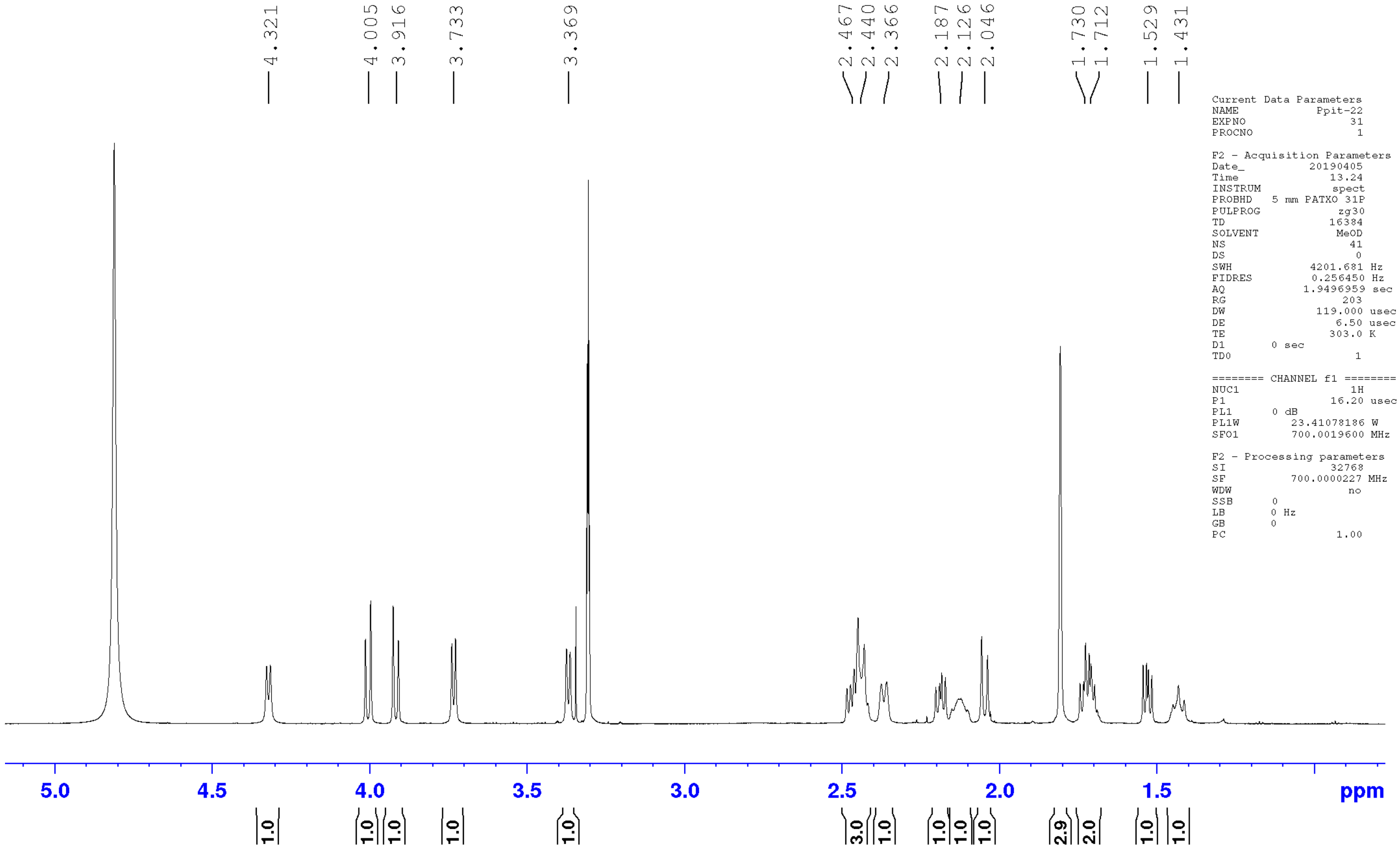

Figure S22. ^{1}H NMR spectrum (700 MHz, CD_3OD) of **1**

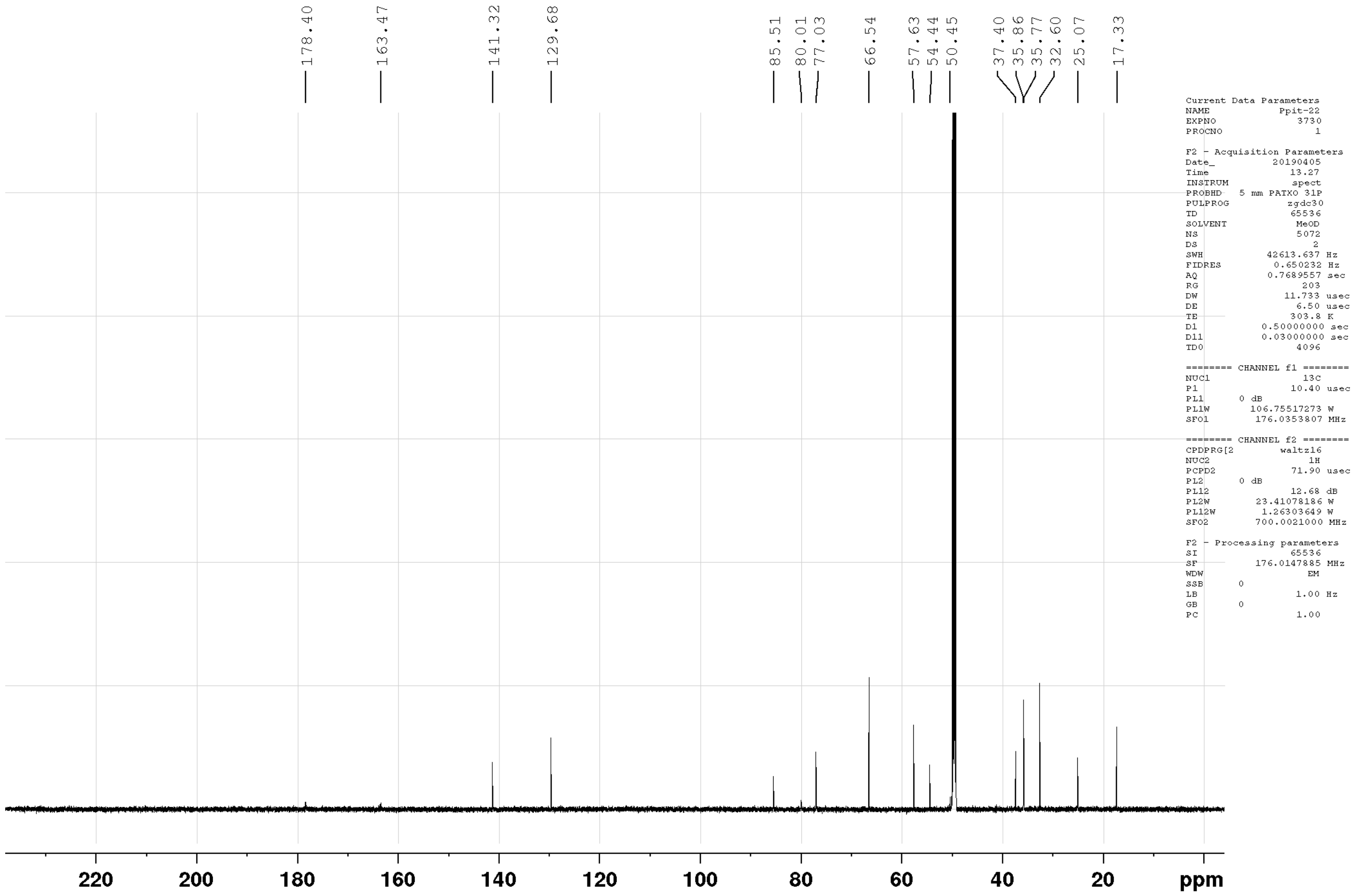

Figure S23. ^{13}C NMR spectrum (176 MHz,CD_3OD) of **1**

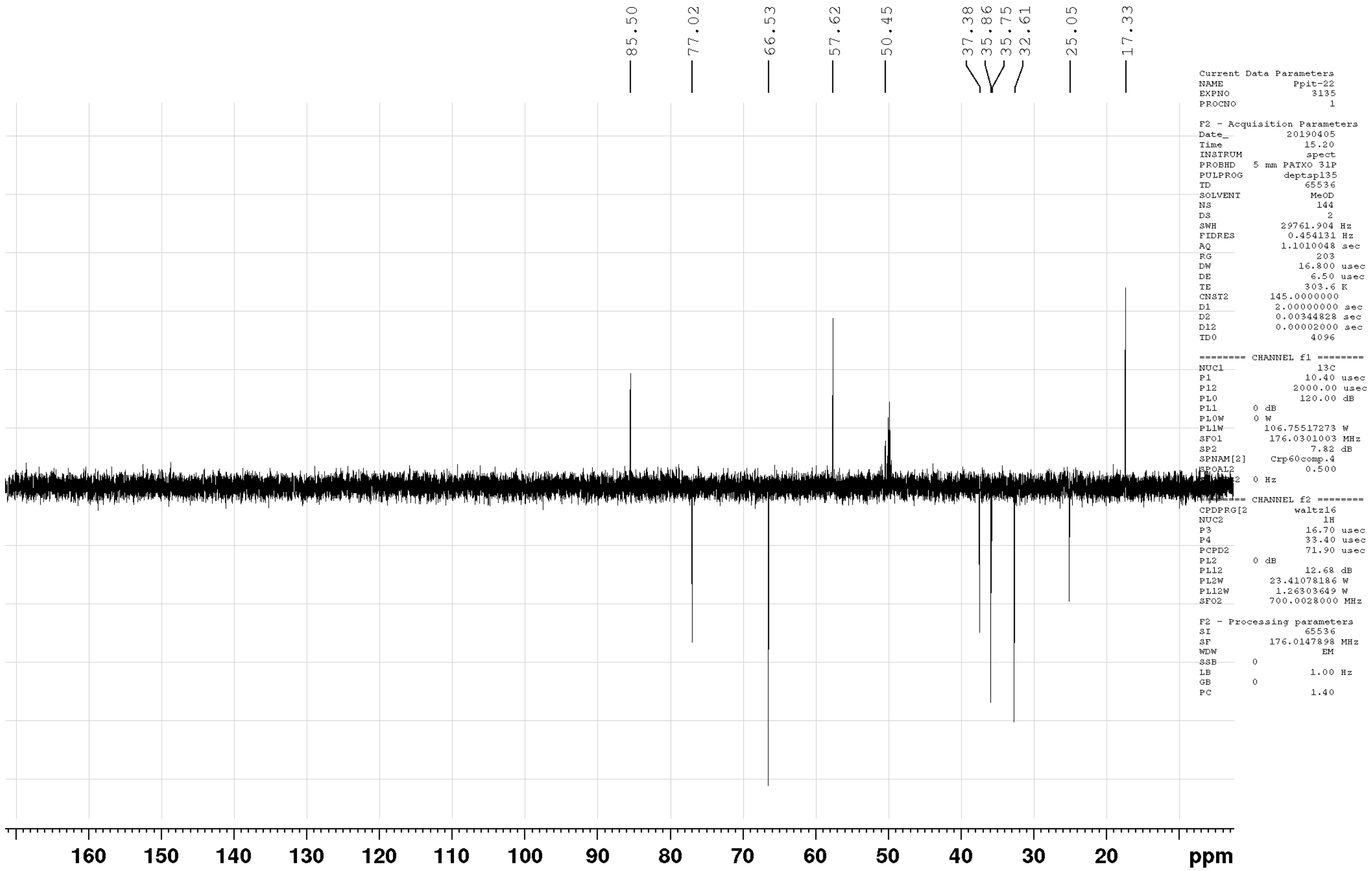

Figure S24. DEPT-135 NMR spectrum (176 MHz, CD_3OD) of **1**

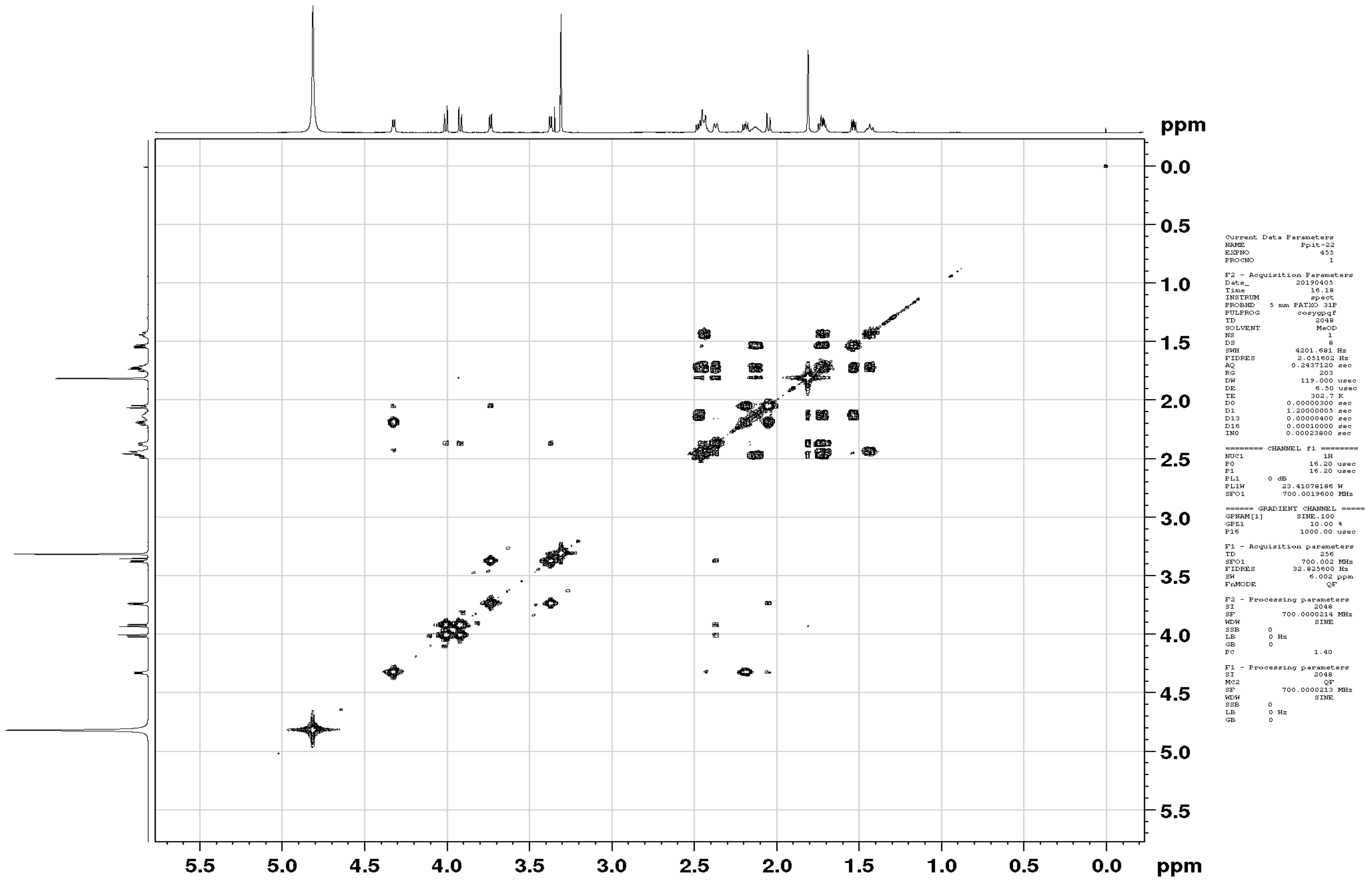

Figure S25. COSY-45 spectrum (700 MHz, CD_3OD) of **1**

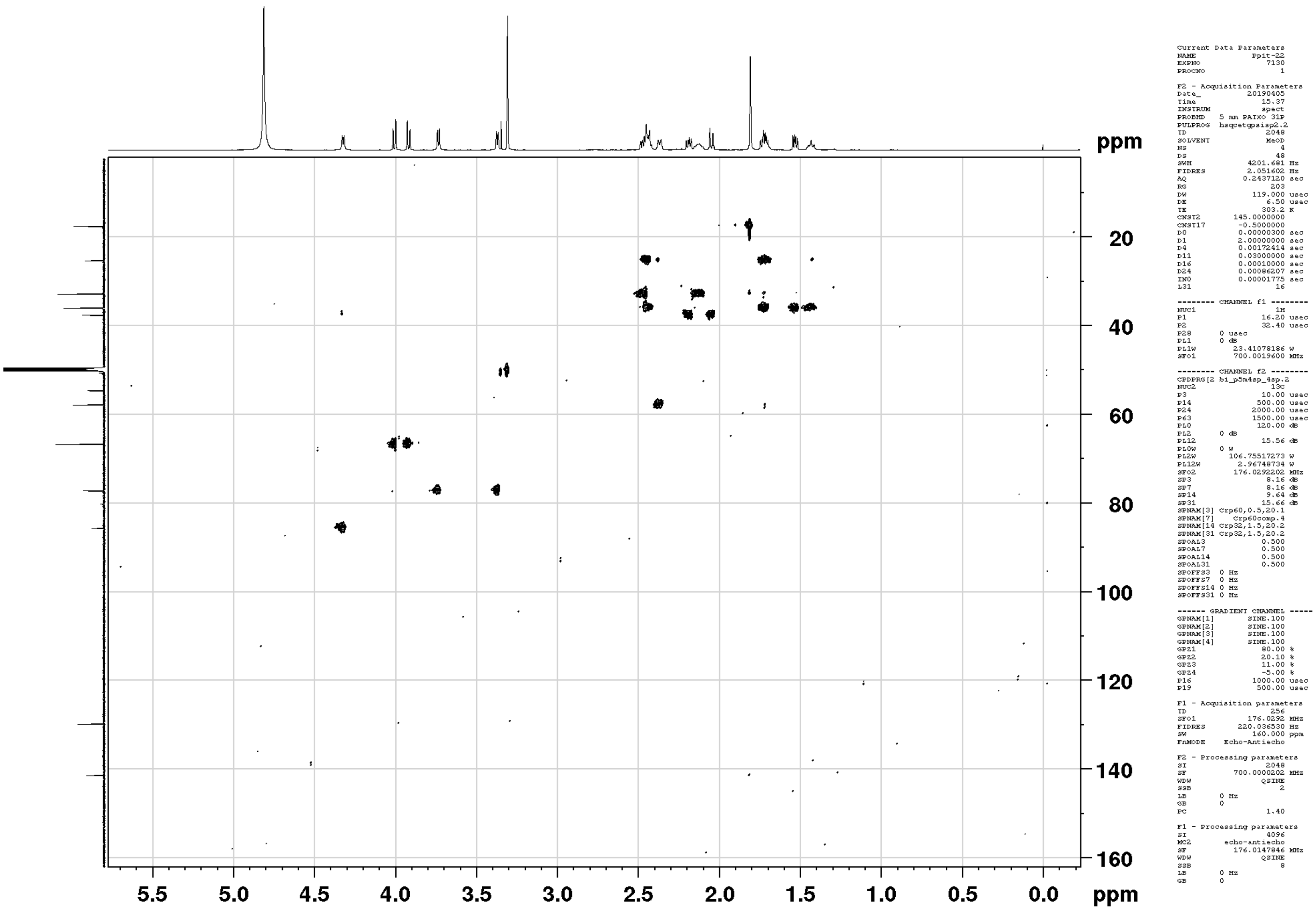

Figure S26. HSQC spectrum (700 MHz, CD_3OD) of **1**

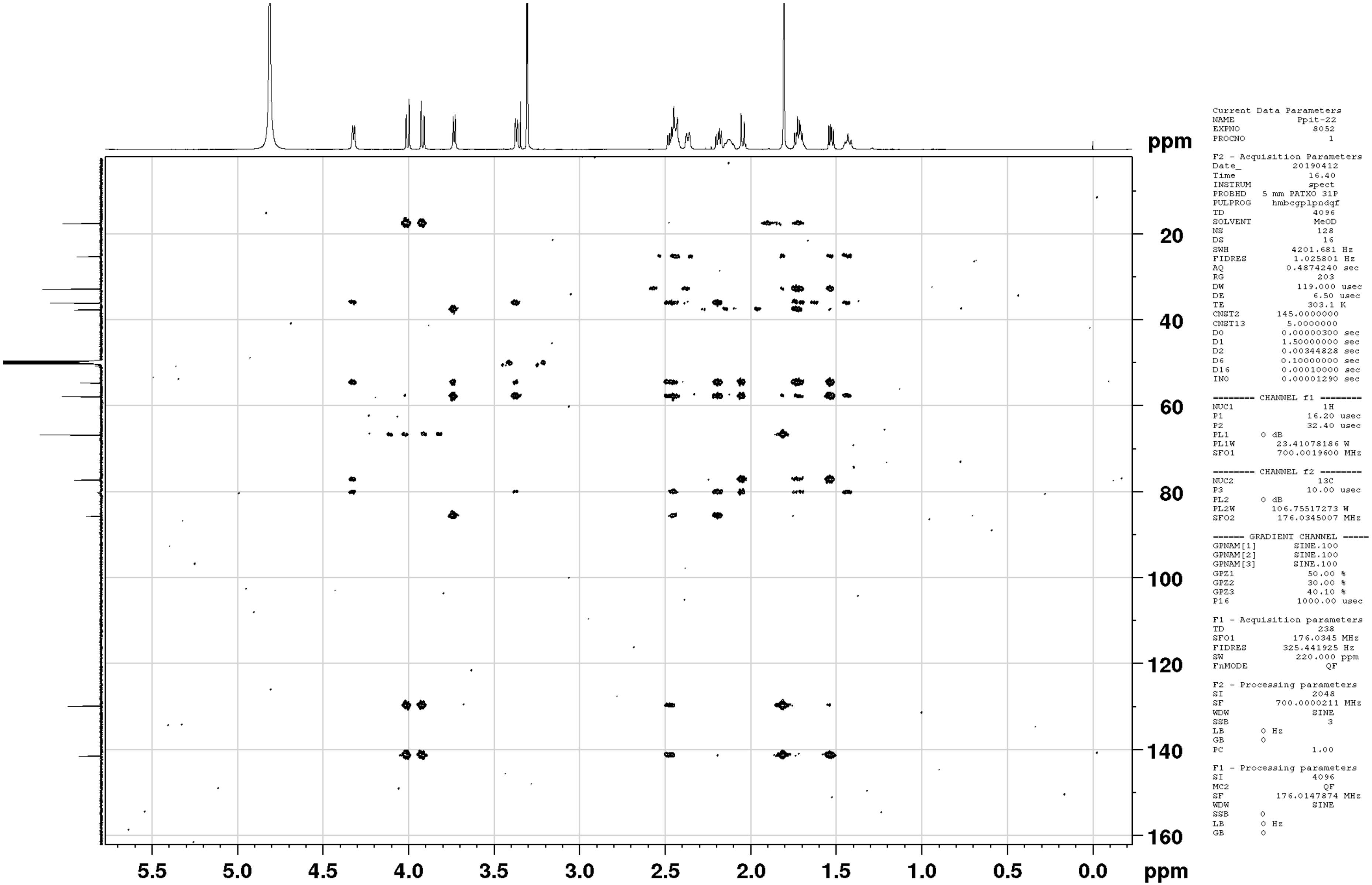

Figure S27. HMBC spectrum (700 MHz, CD_3OD) of **1**

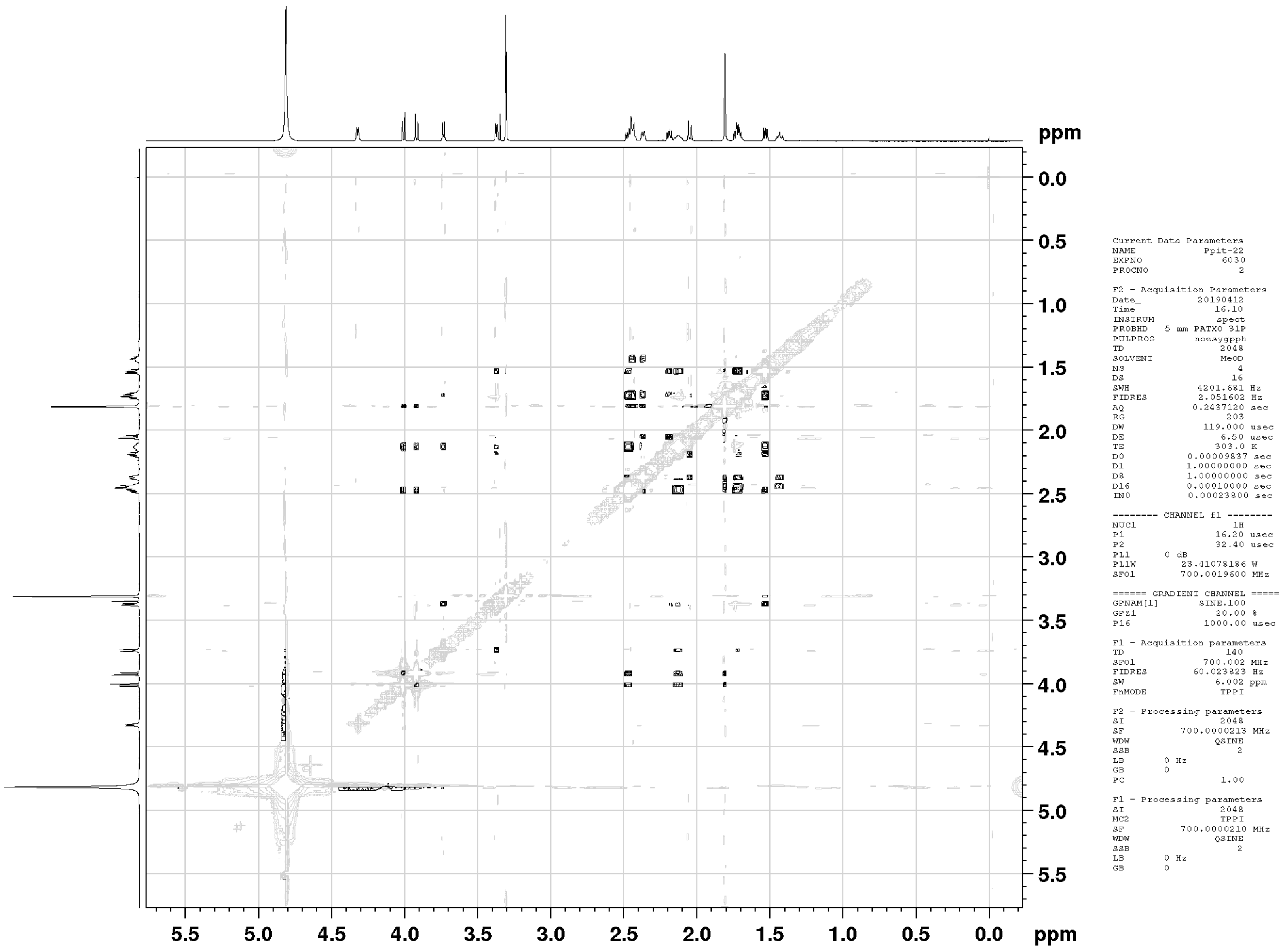

Figure S28. NOESY spectrum (700 MHz, CD_3OD) of **1**

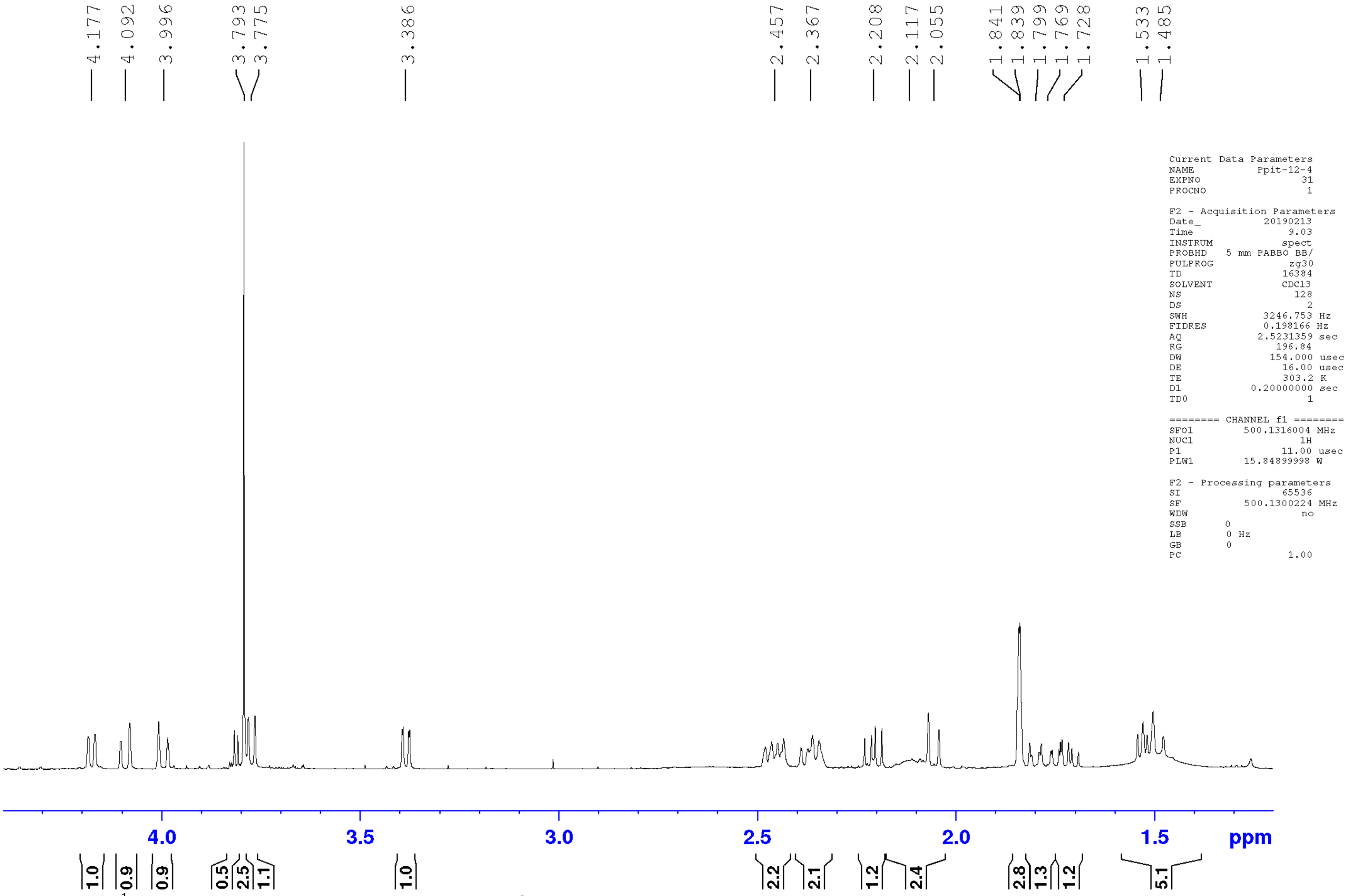

Figure S29. ^{1}H NMR spectrum (500 MHz, CD_3OD) of **1a**

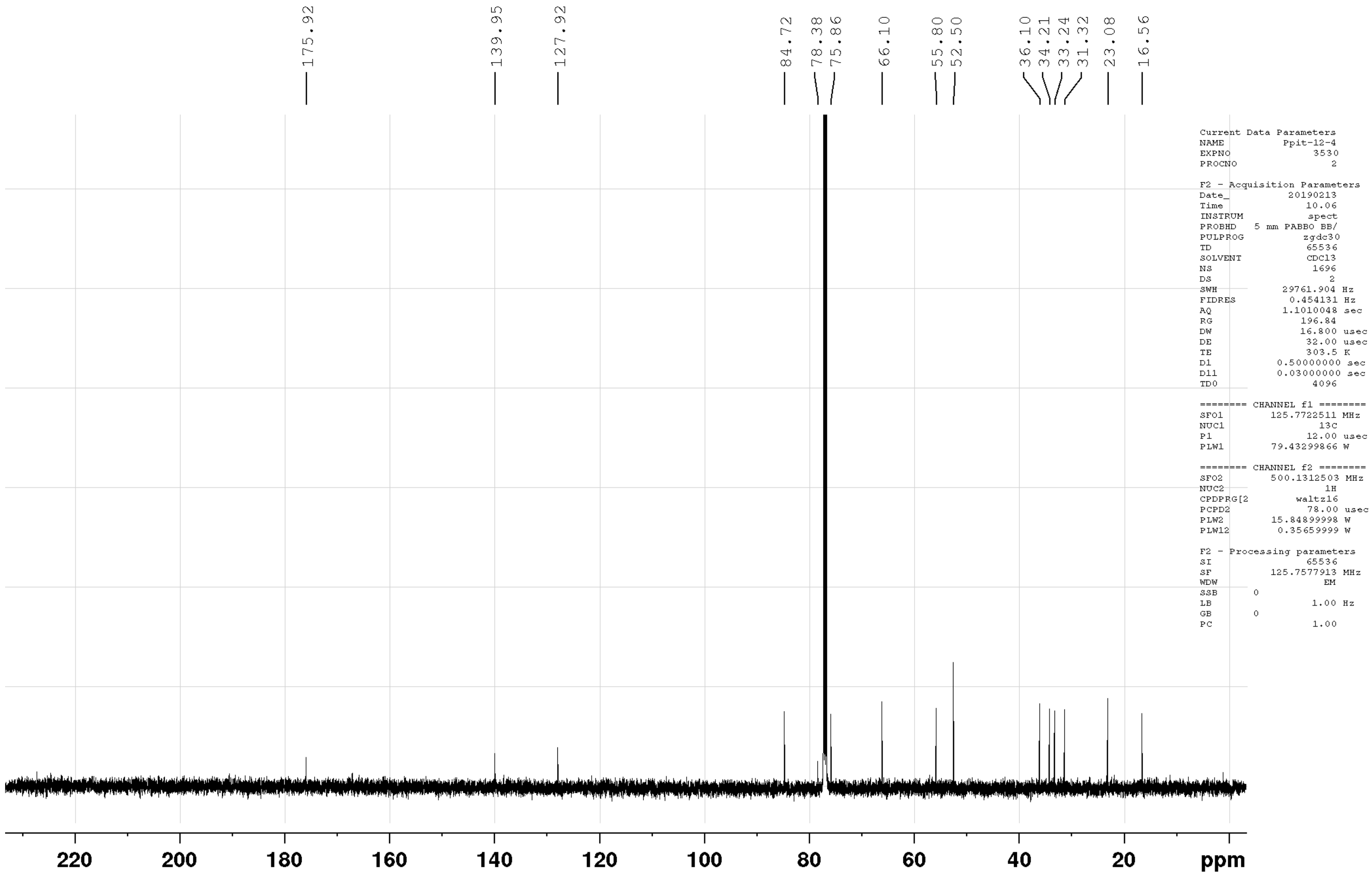

Figure S30. ^{13}C NMR spectrum (125 MHz, CD_3OD) of **1a**

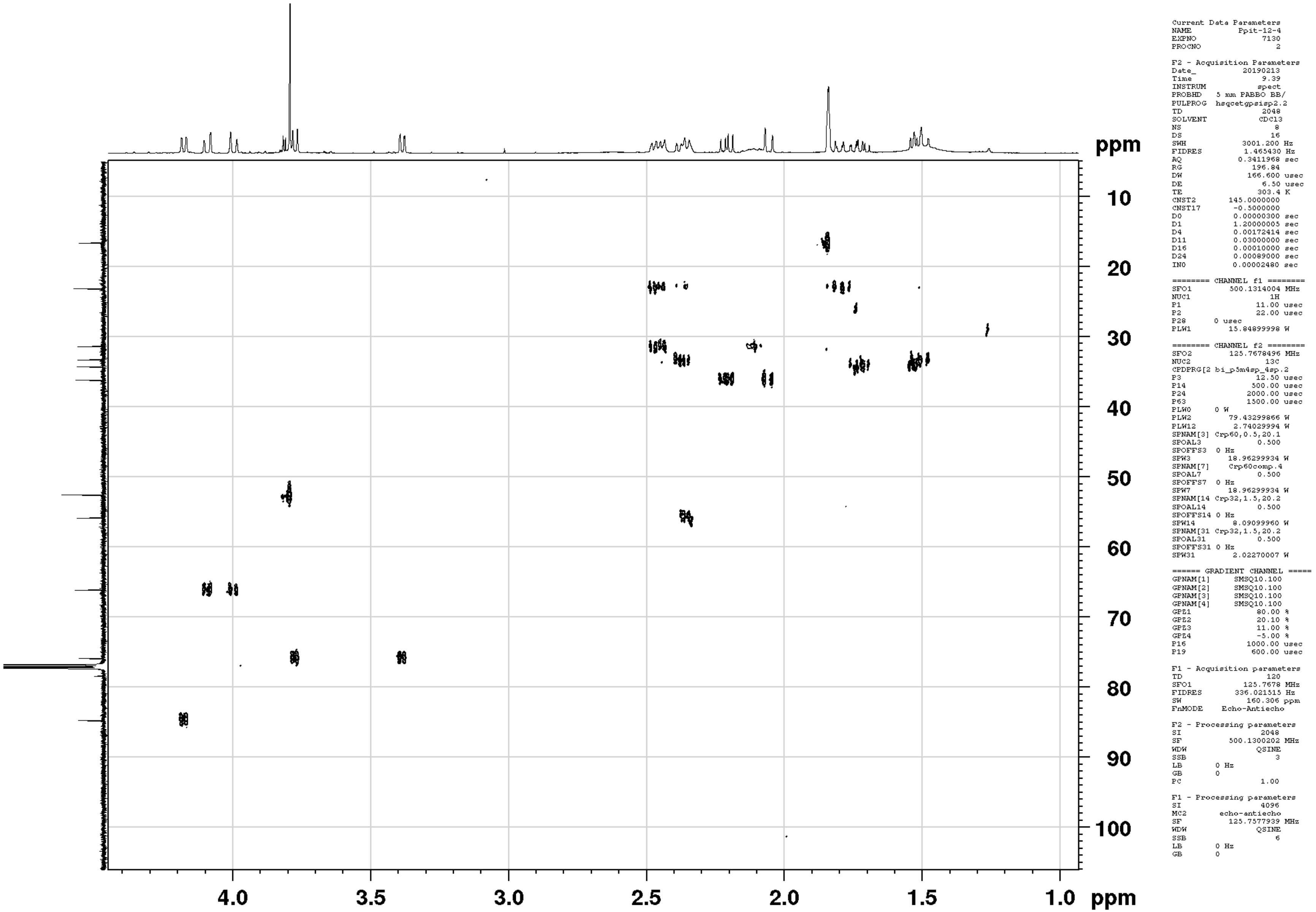

Figure S31. HSQC spectrum (500 MHz, CD_3OD) of **1a**

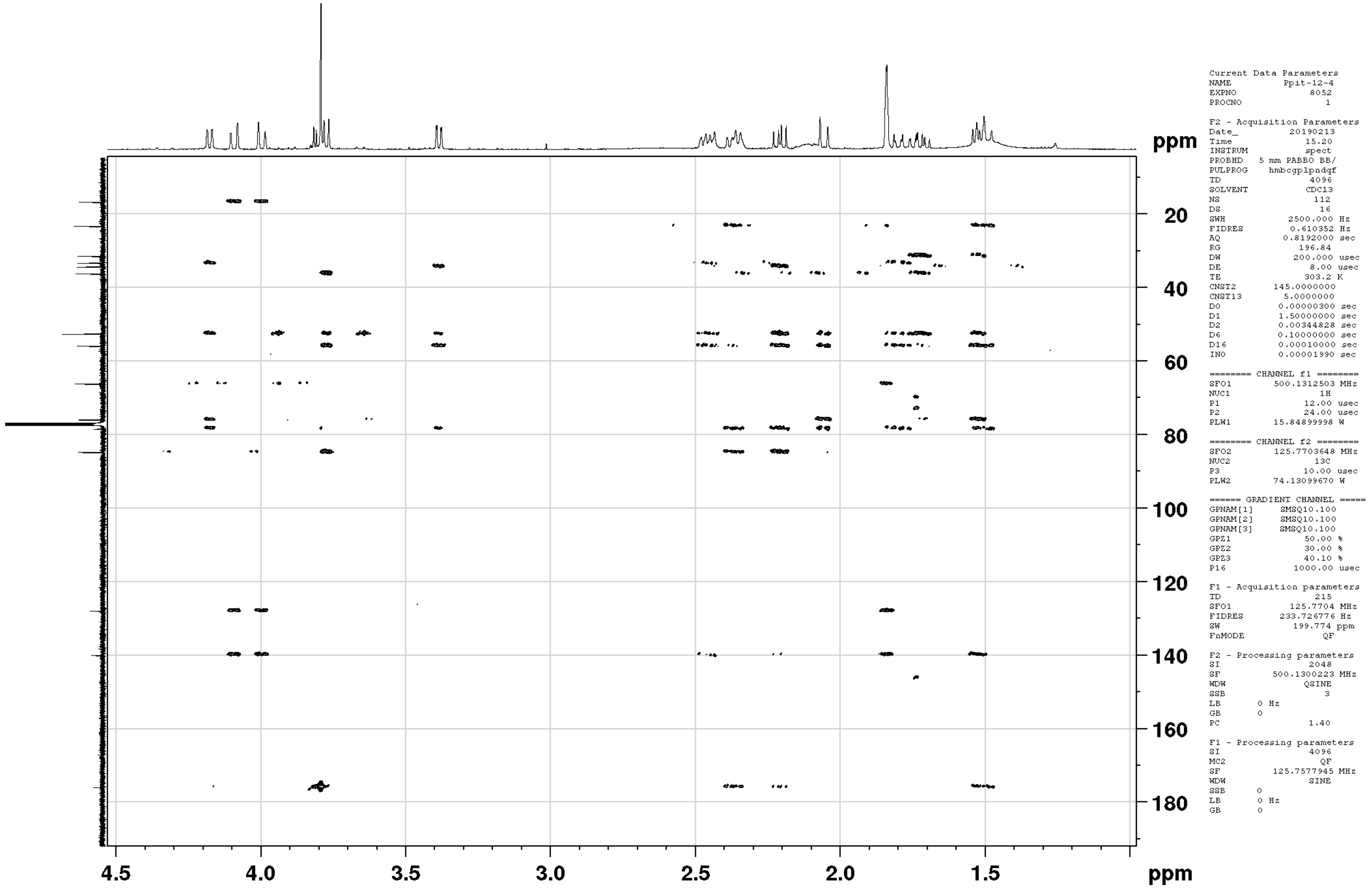

Figure S32. HMBC spectrum (500 MHz, CD_3OD) of **1a**

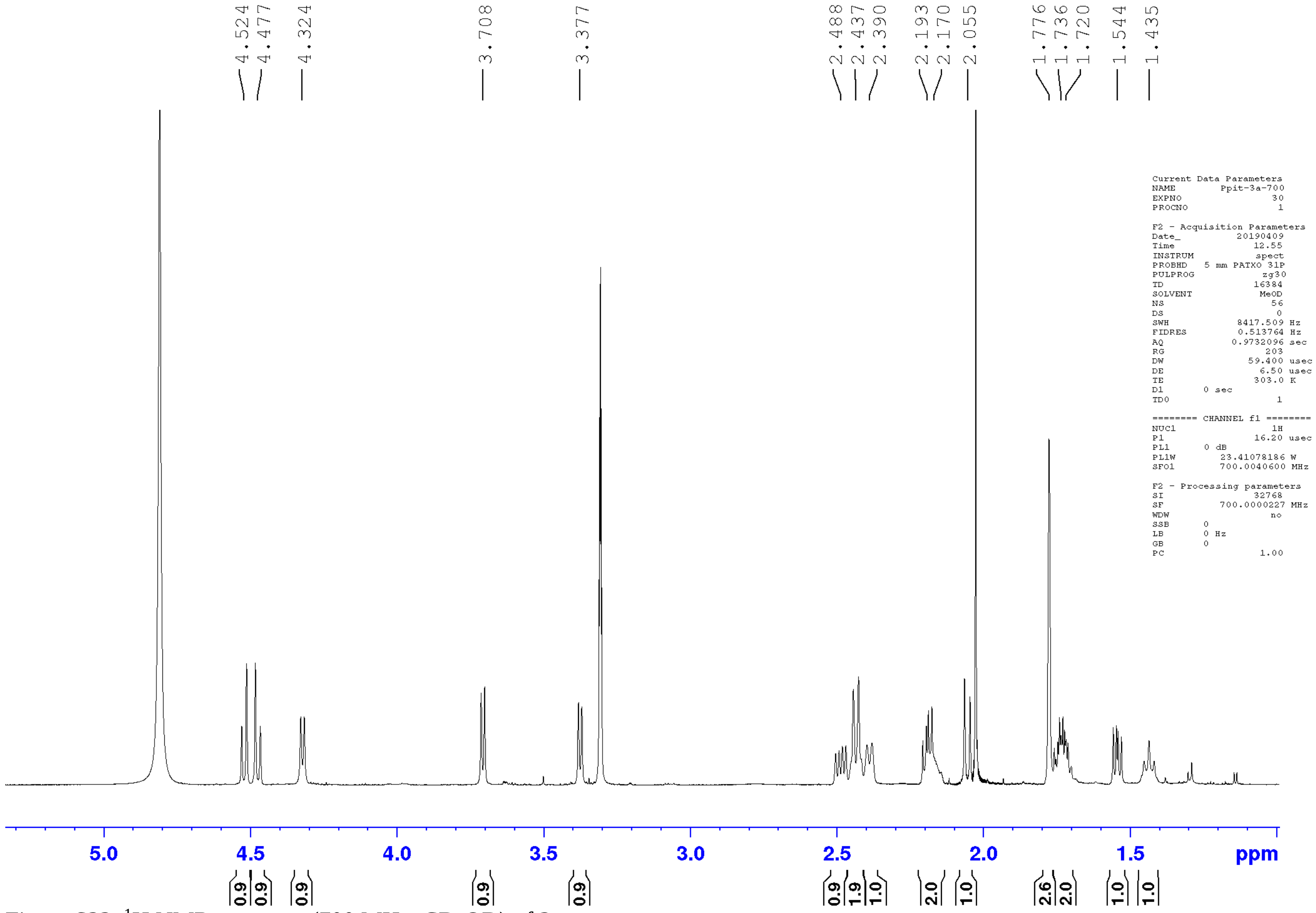

Figure S33. ^{1}H NMR spectrum (700 MHz, CD_3OD) of **2**

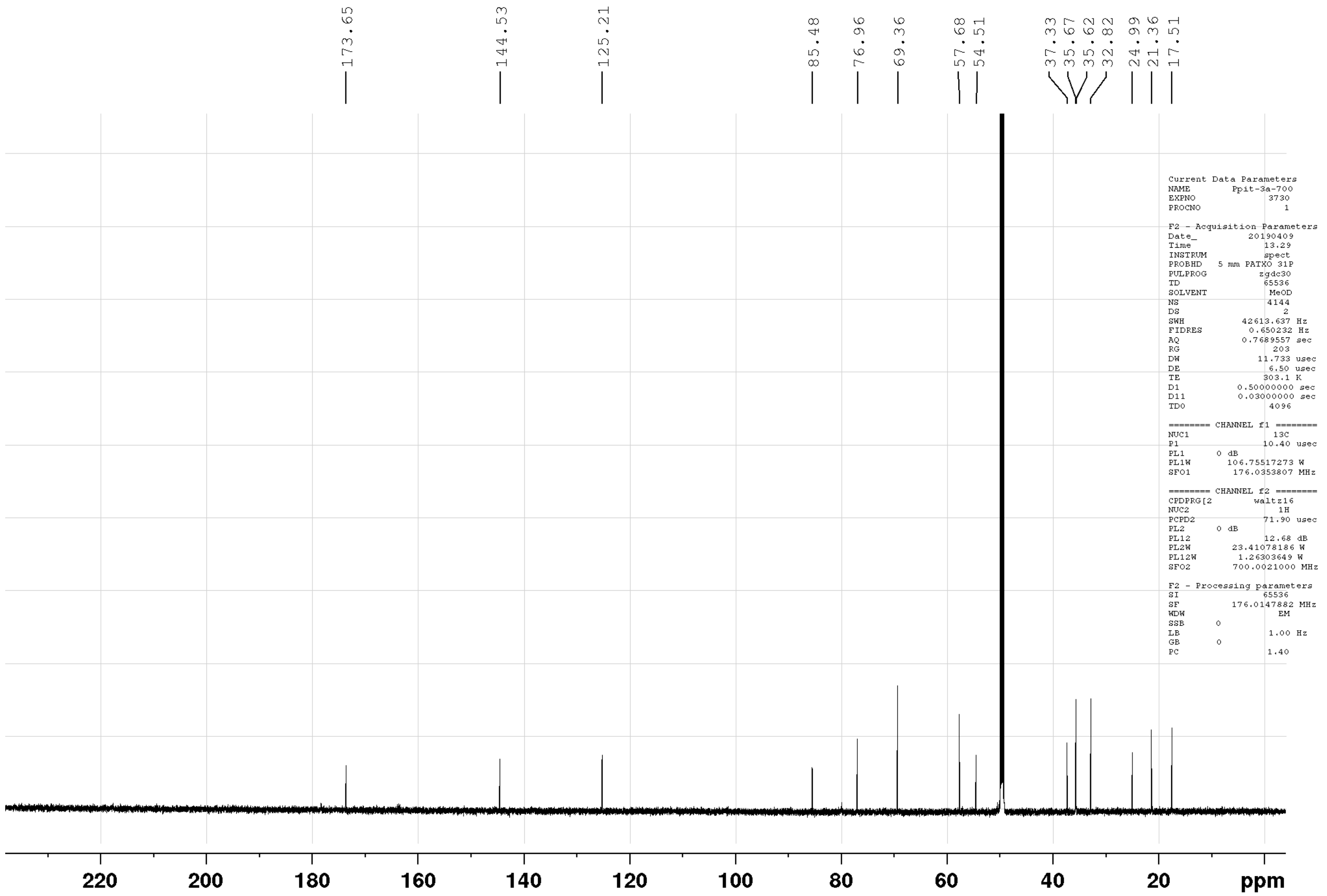

Figure S34. ^{13}C NMR spectrum (176 MHz, CD_3OD) of **2**

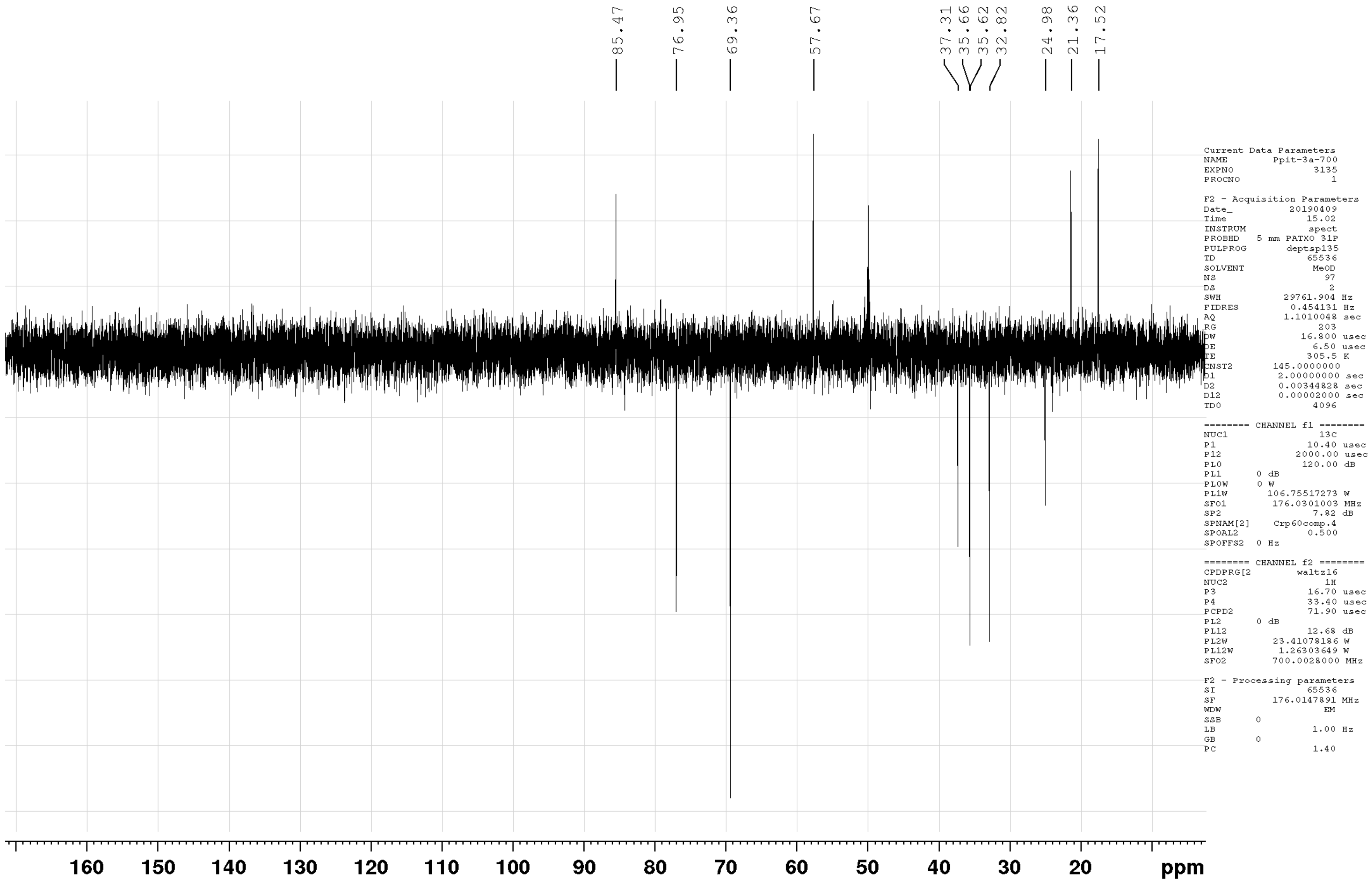

Figure S35. DEPT-135 NMR spectrum (176 MHz, CD_3OD) of **2**

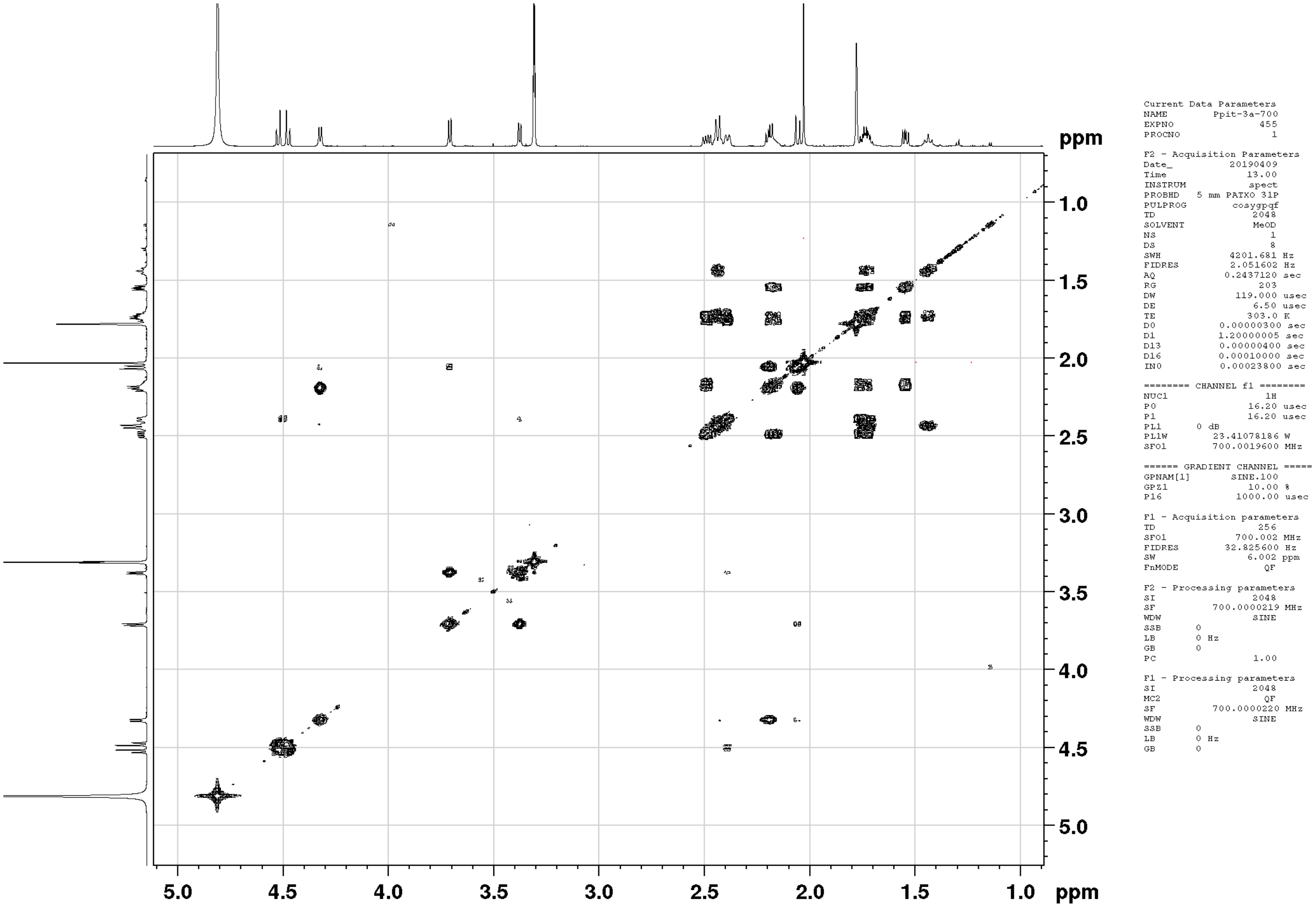

Figure S36. COSY-45 spectrum (700 MHz, CD_3OD) of **2**

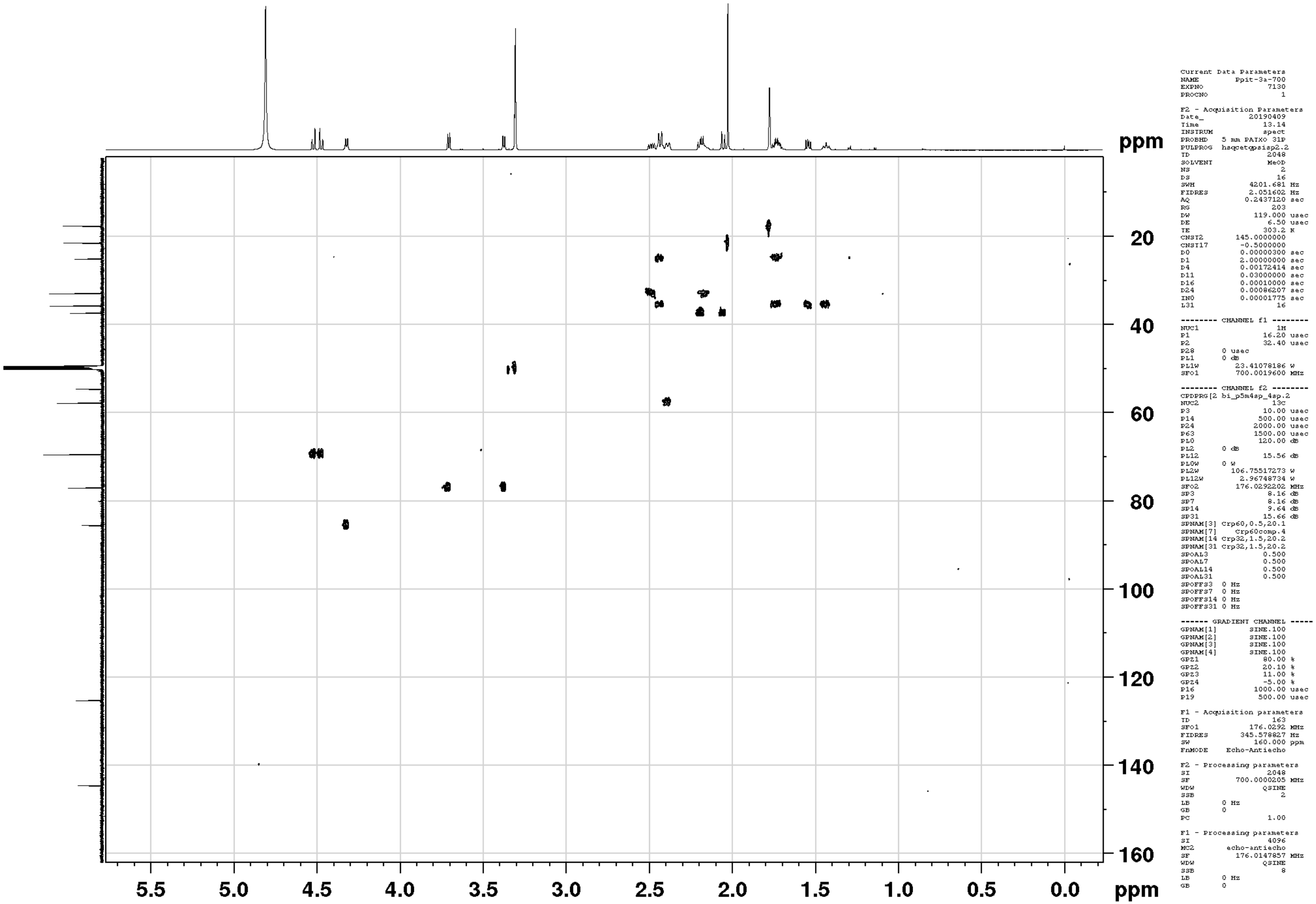

Figure S37. HSQC spectrum (700 MHz, CD_3OD) of **2**

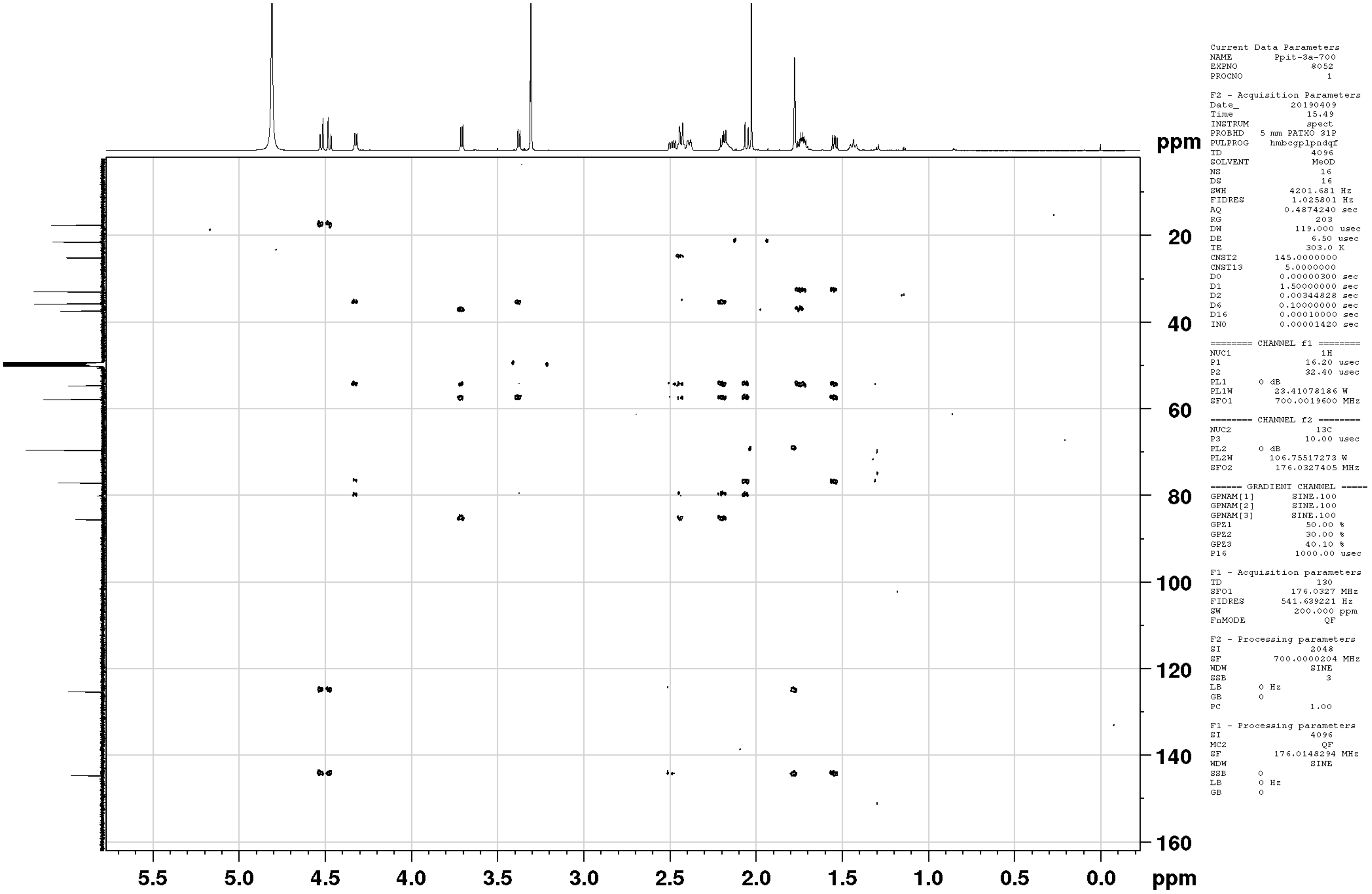

Figure S38. HMBC spectrum (700 MHz, CD_3OD) of **2**

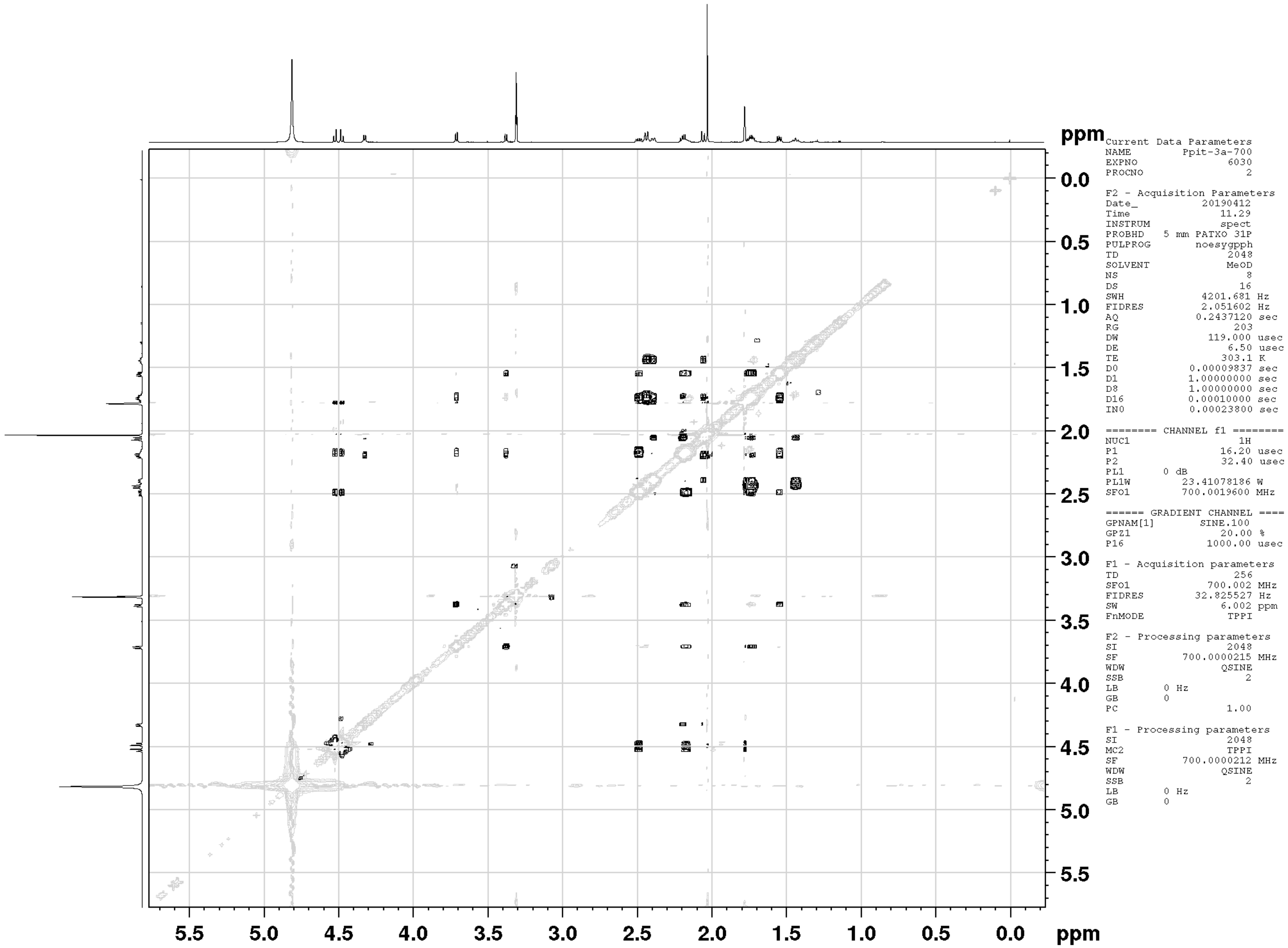

Figure S39. NOESY spectrum (700 MHz, CD_3OD) of **2**

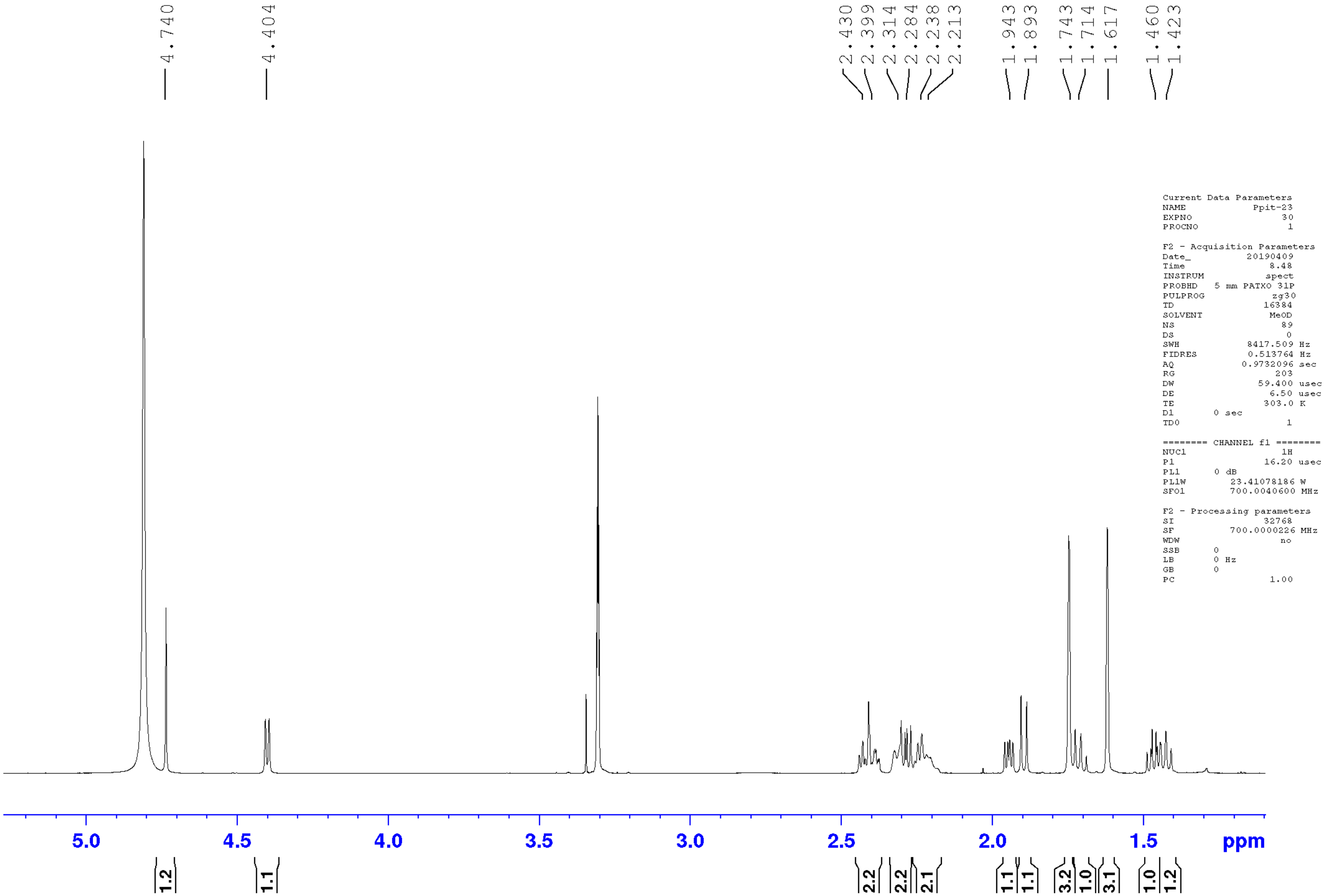

Figure S40. ^{1}H NMR spectrum (700 MHz, CD_3OD) of **3**

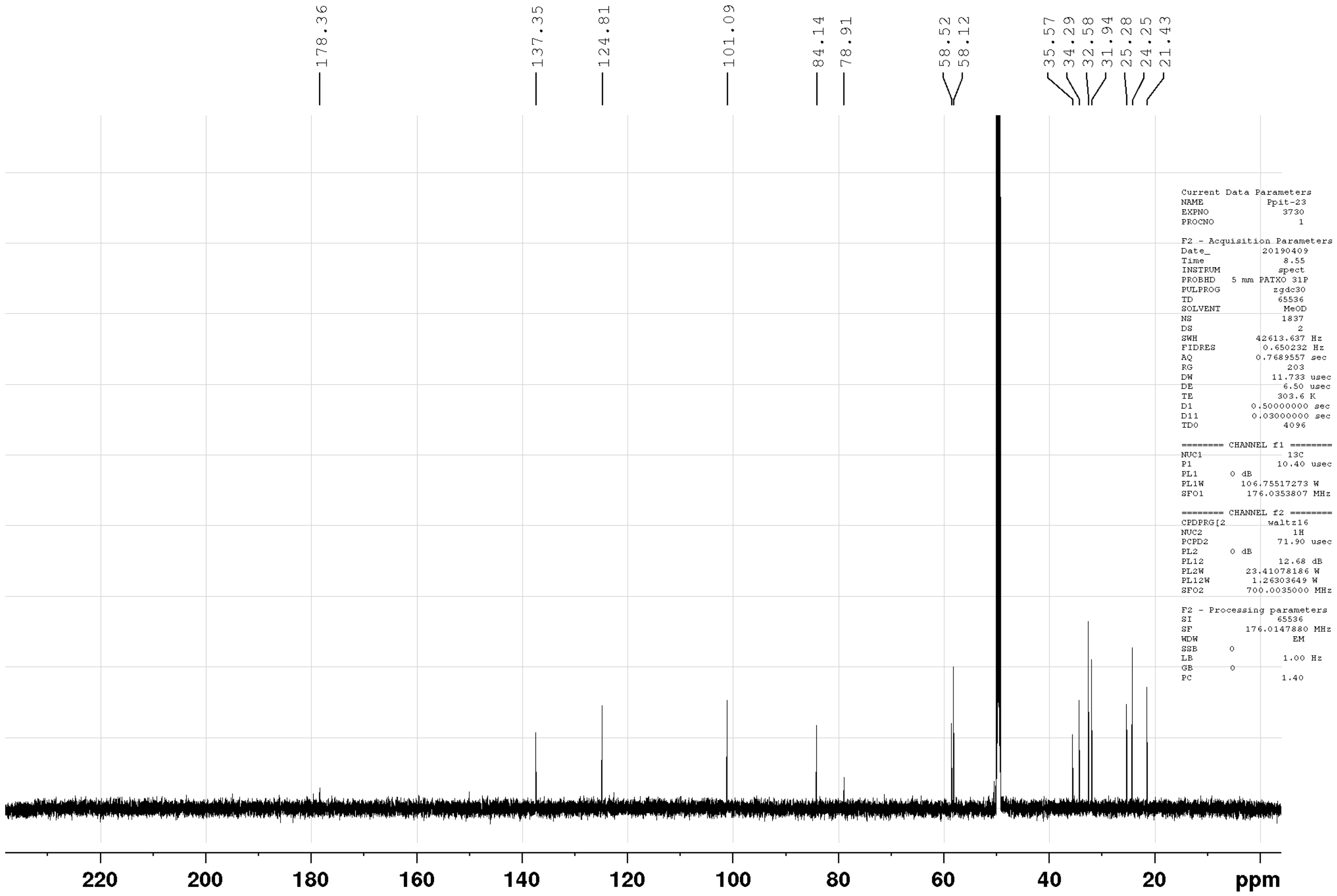

Figure S41. ^{13}C NMR spectrum (176 MHz, CD_3OD) of **3**

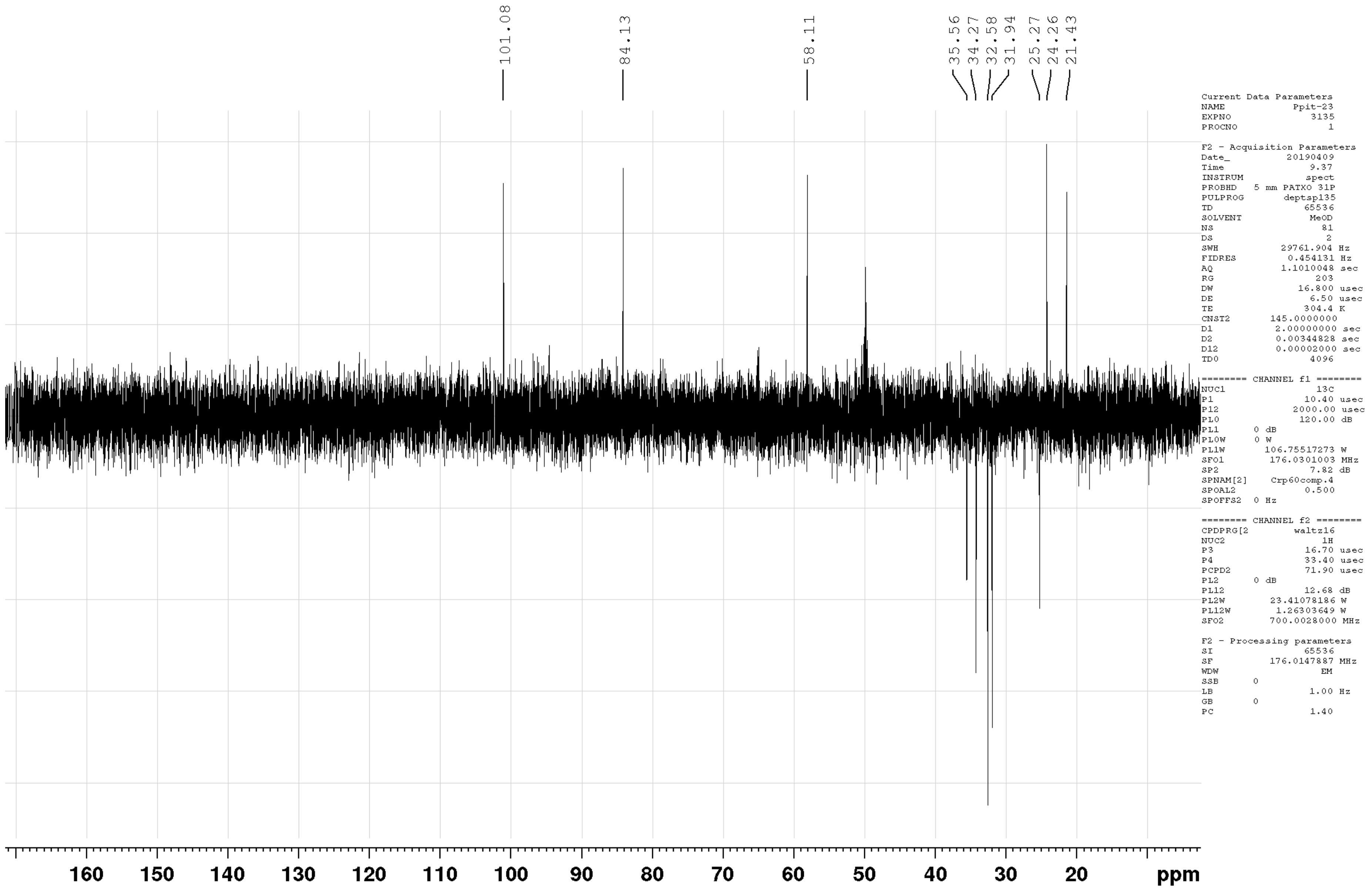

Figure S42. DEPT-135 NMR spectrum (176 MHz, CD_3OD) of **3**

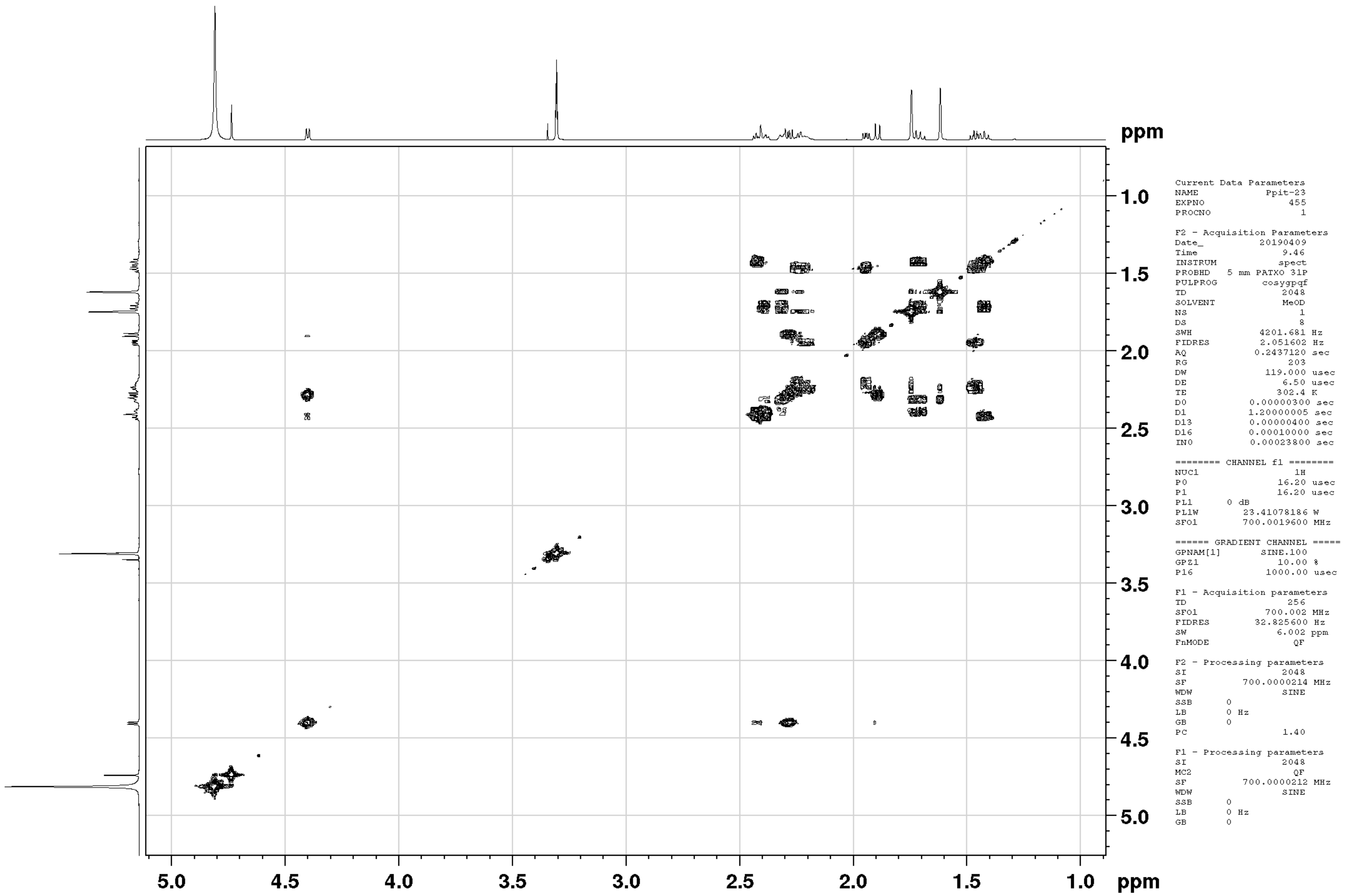

Figure S43. COSY-45 spectrum (700 MHz, CD_3OD) of **3**

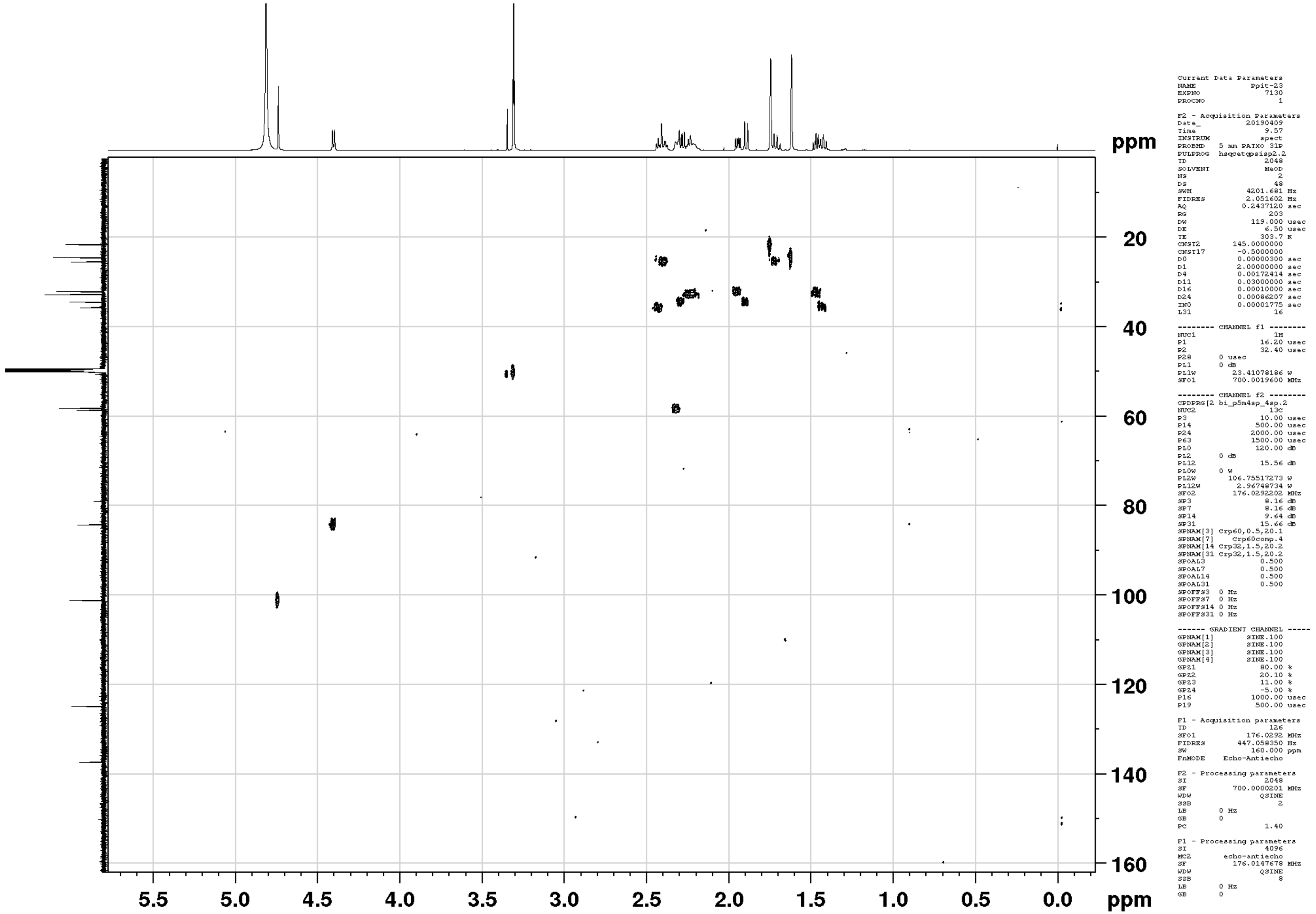

Figure S44. HSQC spectrum (700 MHz, CD_3OD) of **3**

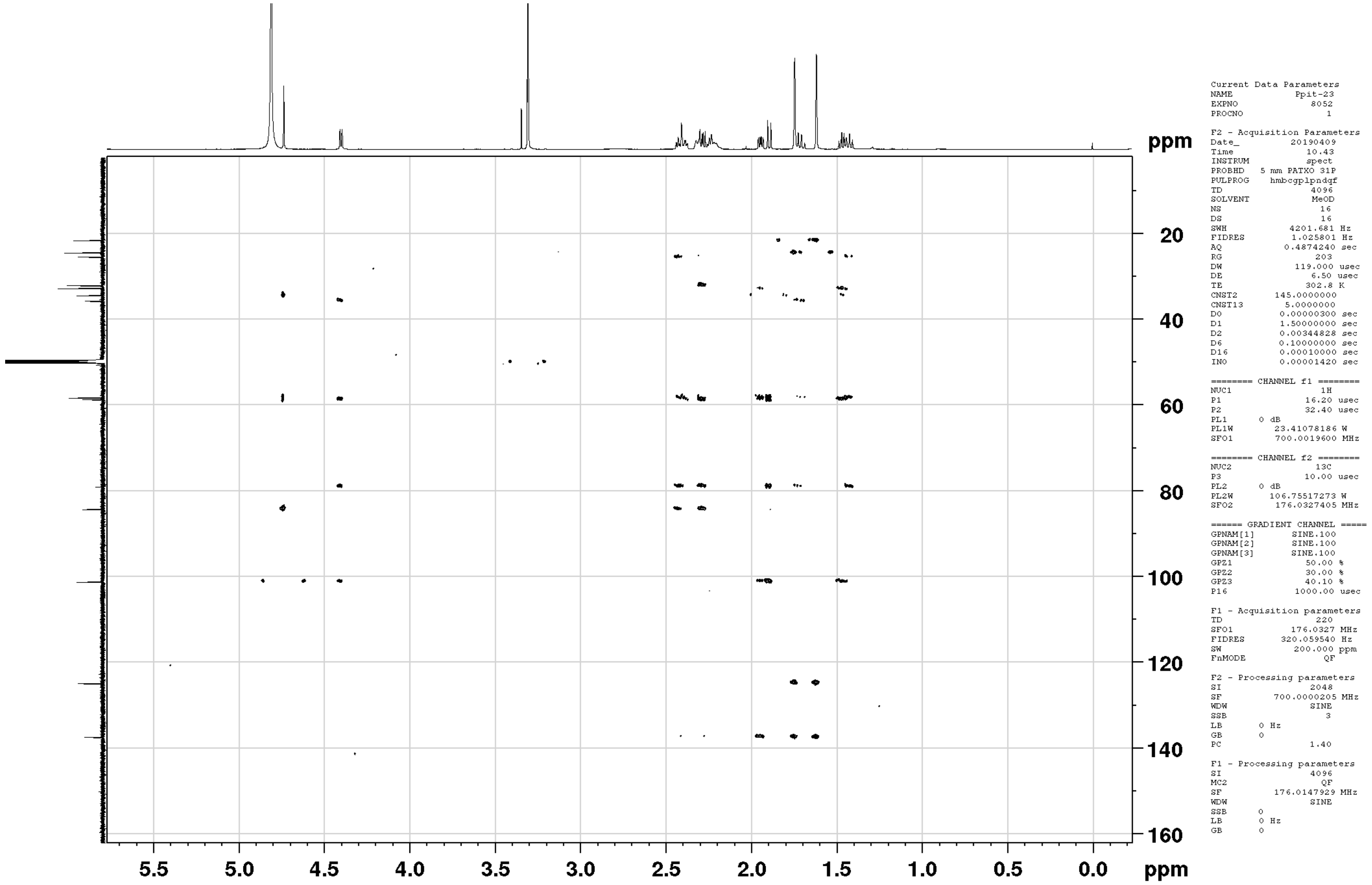

Figure S45. HMBC spectrum (700 MHz, CD_3OD) of **3**

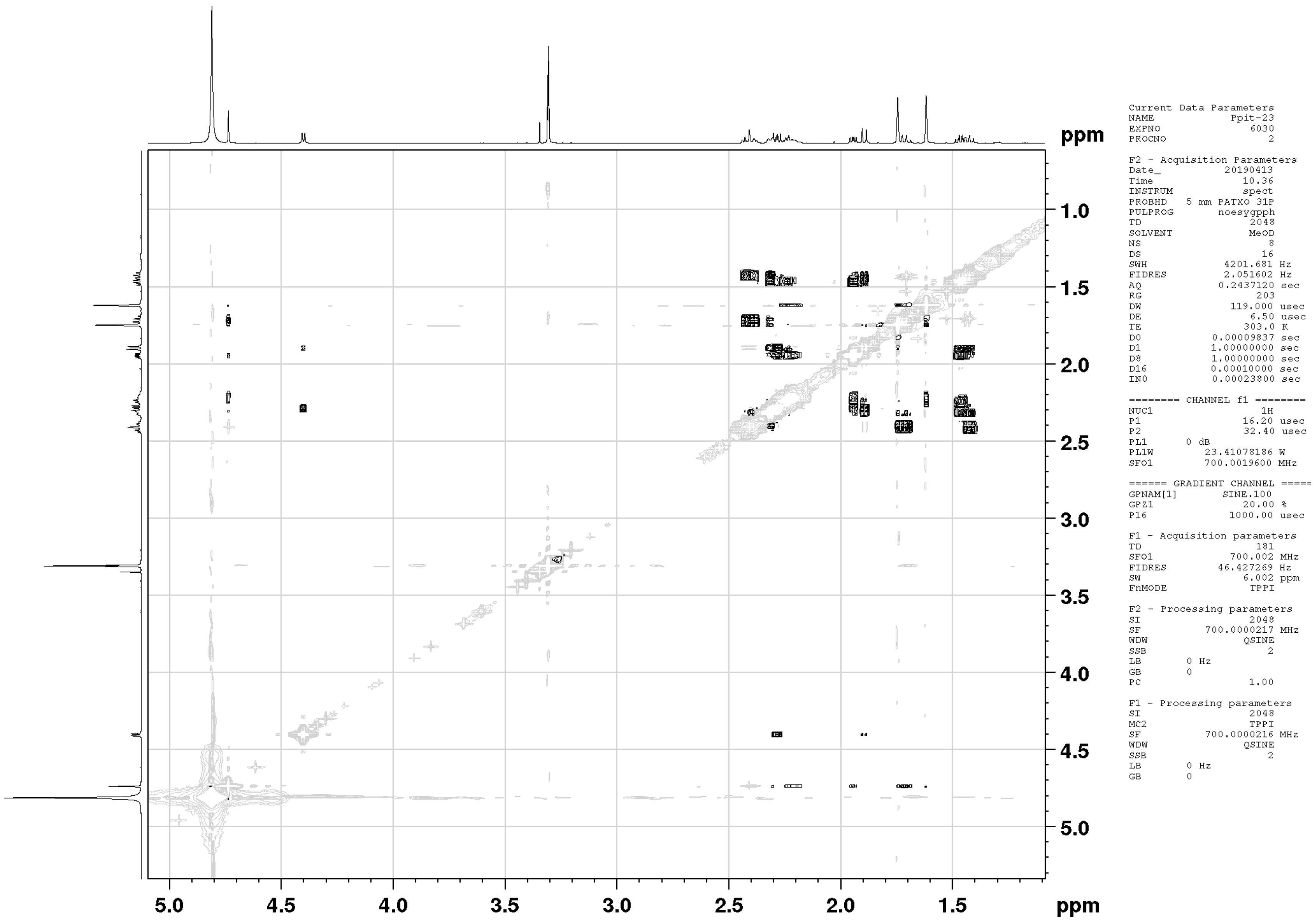

Figure S46. NOESY spectrum (700 MHz, CD_3OD) of **3**

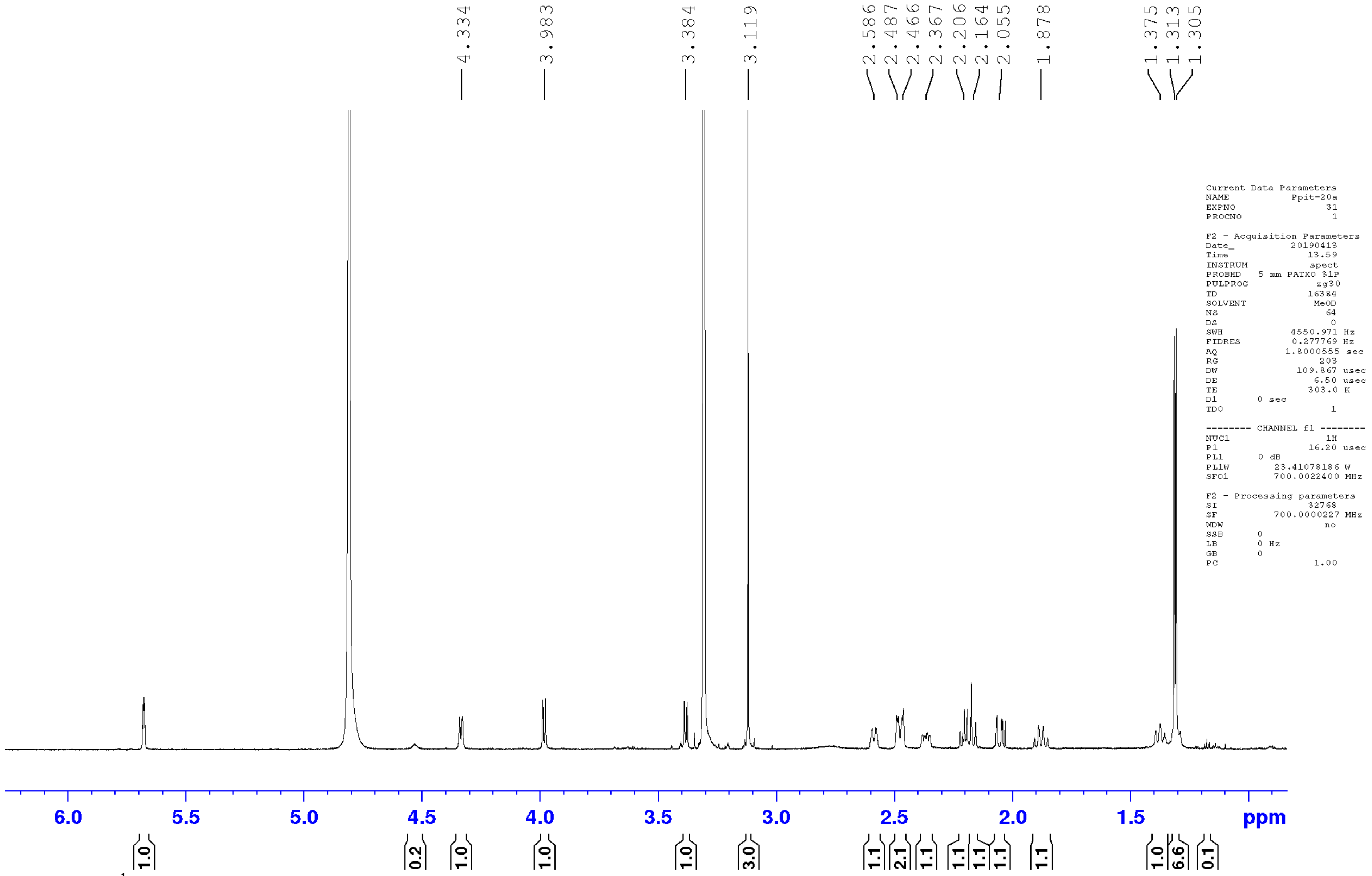

Figure S47. ^{1}H NMR spectrum (700 MHz, CD_3OD) of **4**

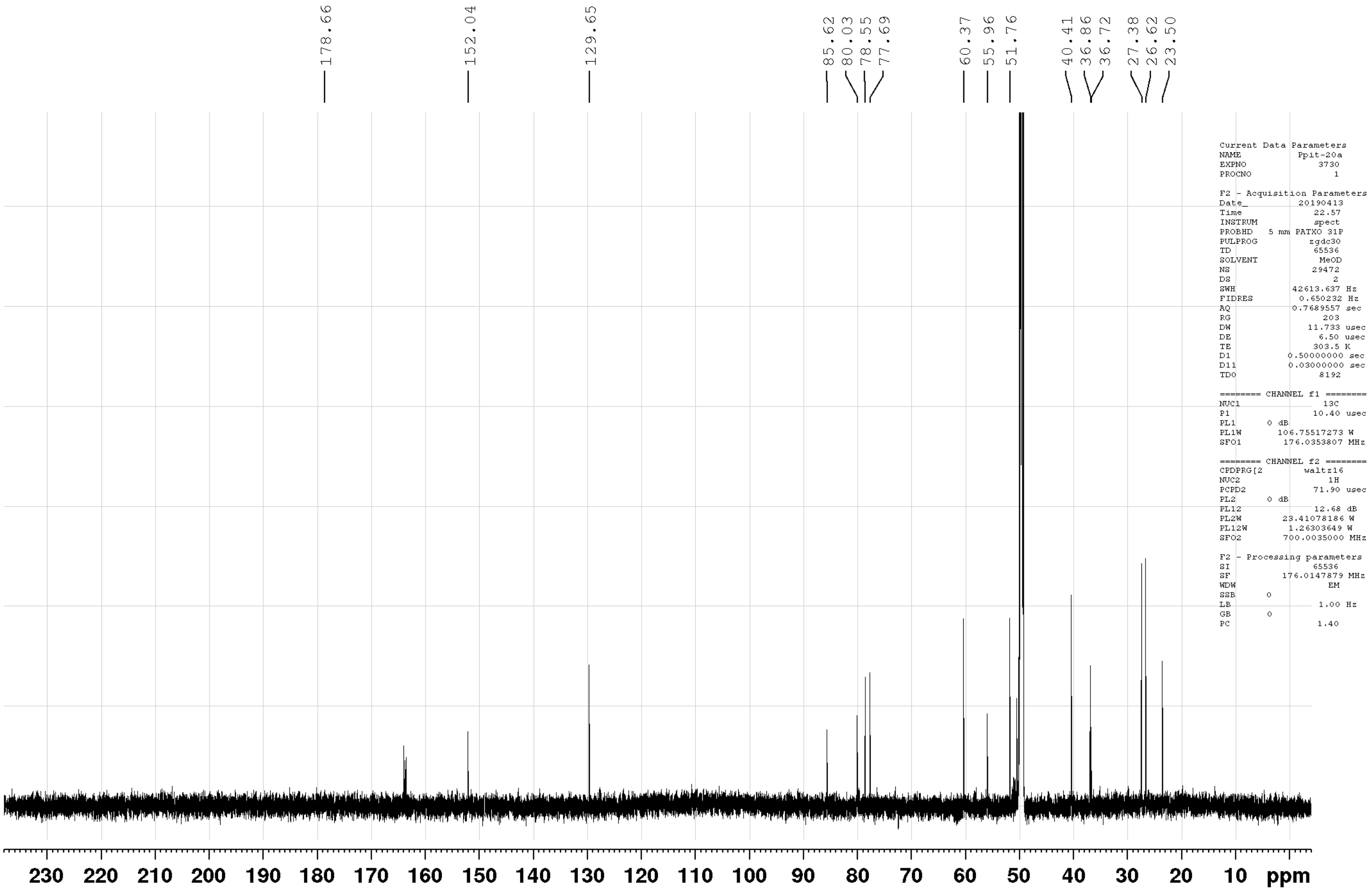

Figure S48. ^{13}C NMR spectrum (176 MHz, CD_3OD) of **4**

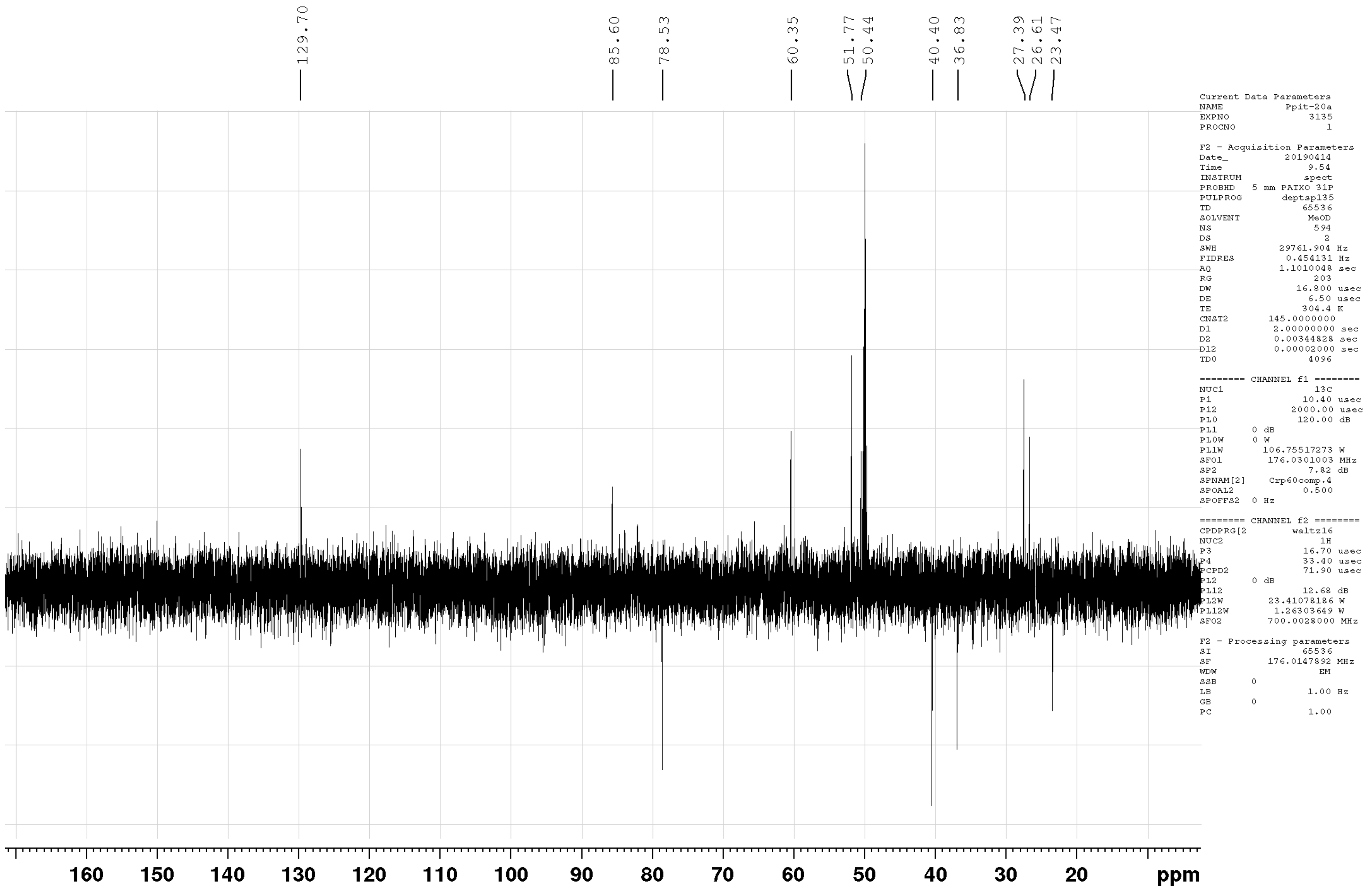

Figure S49. DEPT-135 NMR spectrum (176 MHz, CD_3OD) of **4**

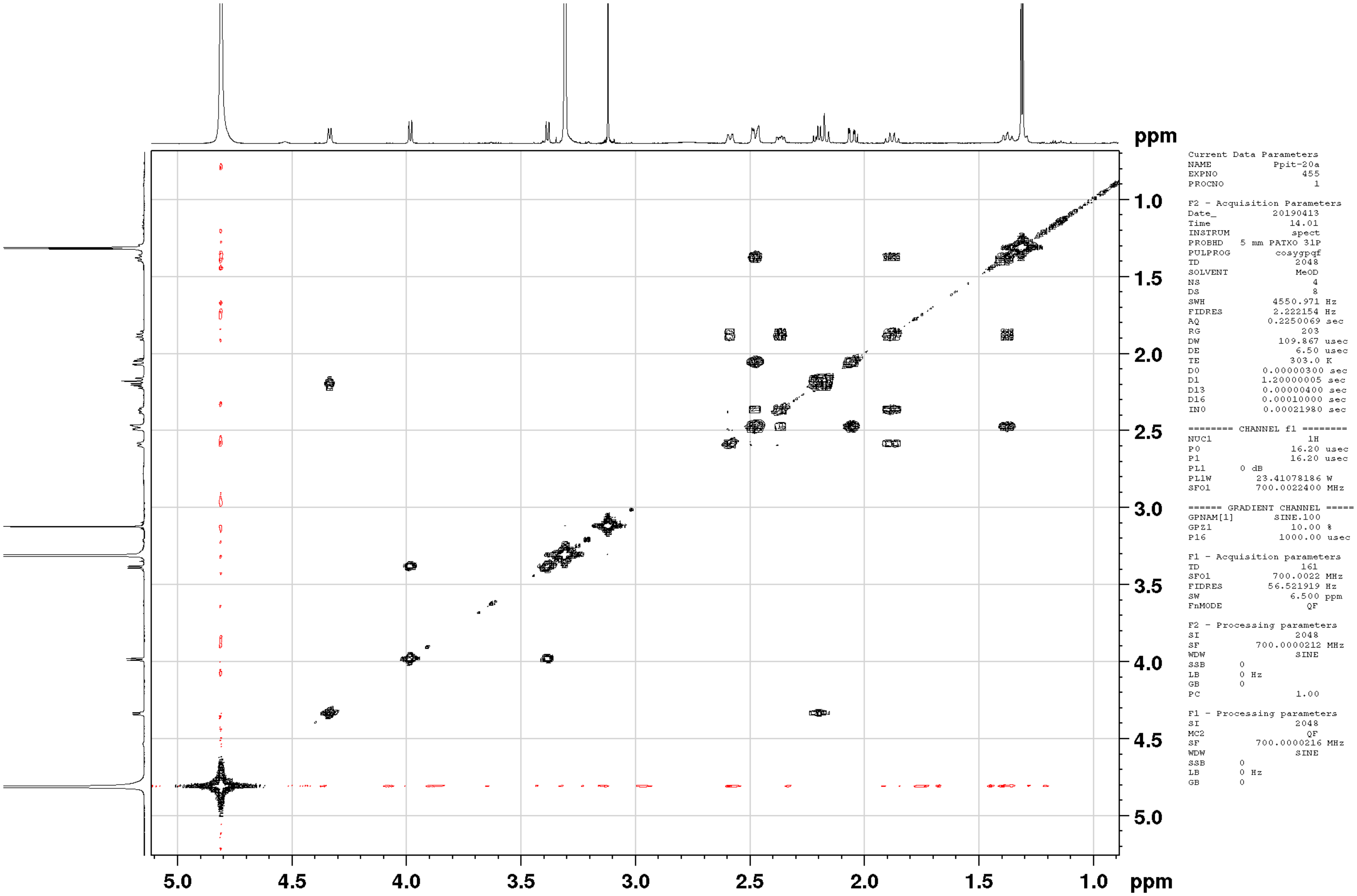

Figure S50. COSY-45 spectrum (700 MHz, CD_3OD) of **4**

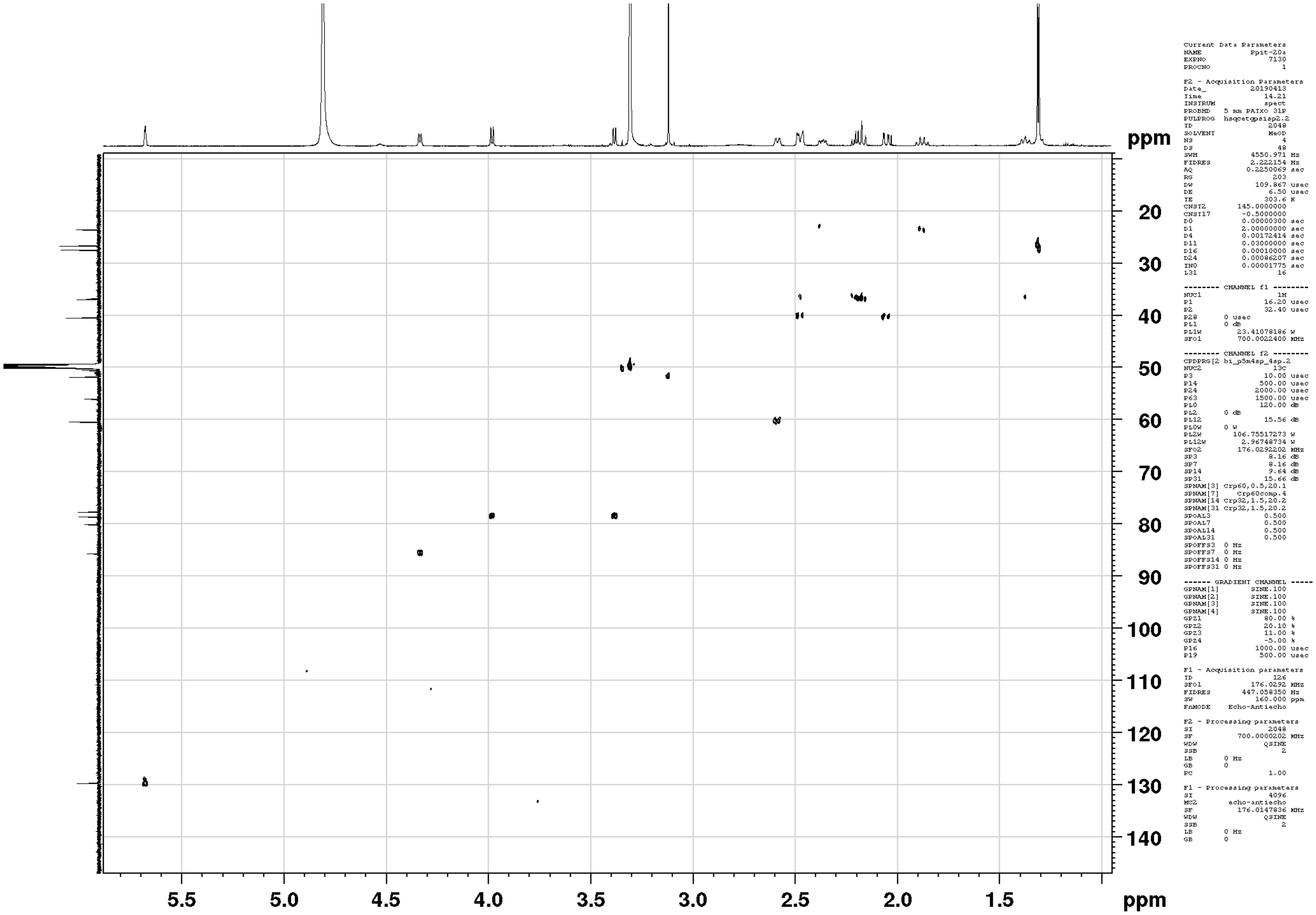

Figure S51. HSQC spectrum (700 MHz, CD_3OD) of **4**

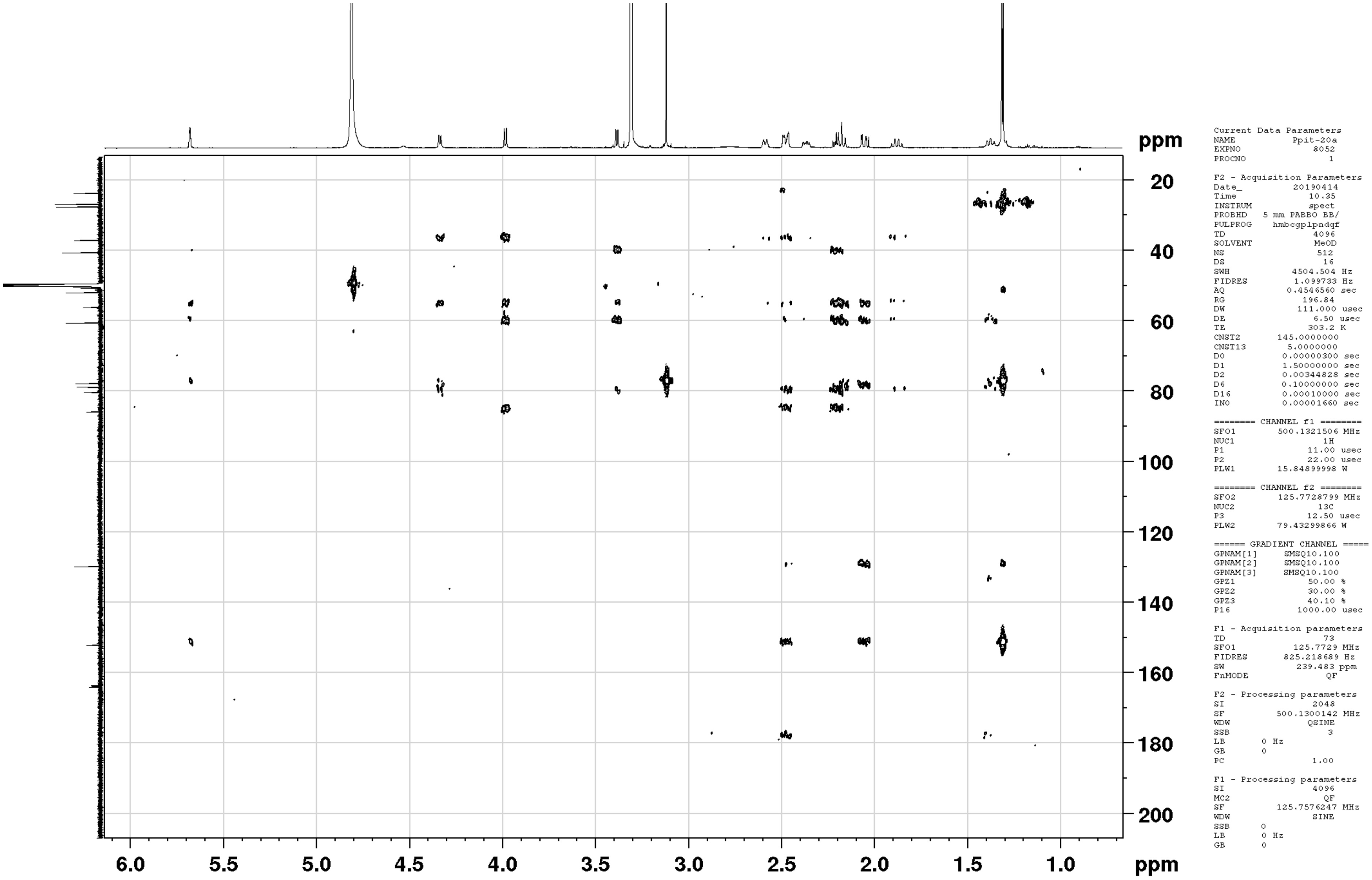

Figure S52. HMBC spectrum (500 MHz, CD_3OD) of **4**

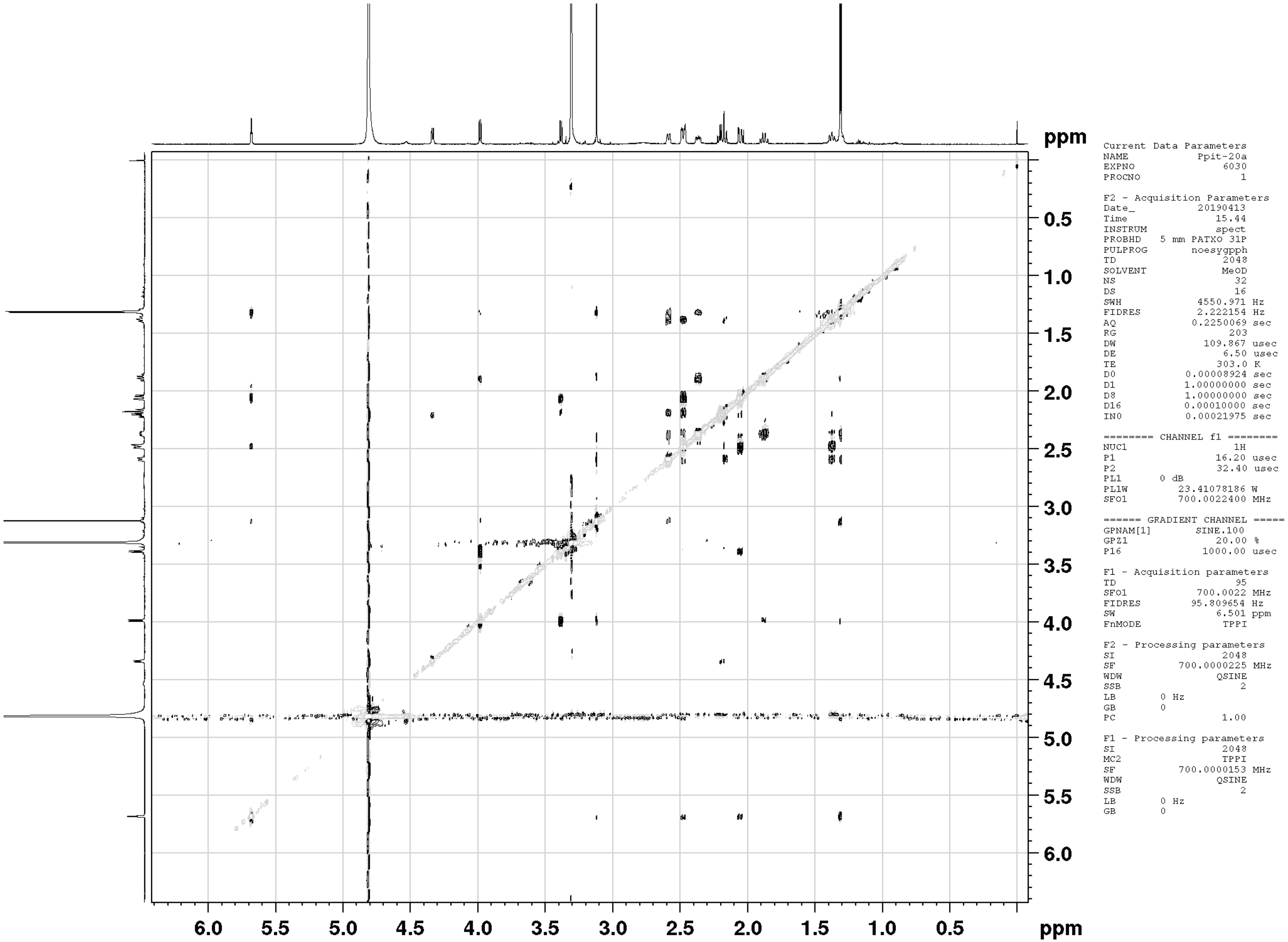

Figure S53. NOESY spectrum (700 MHz, CD_3OD) of **4**

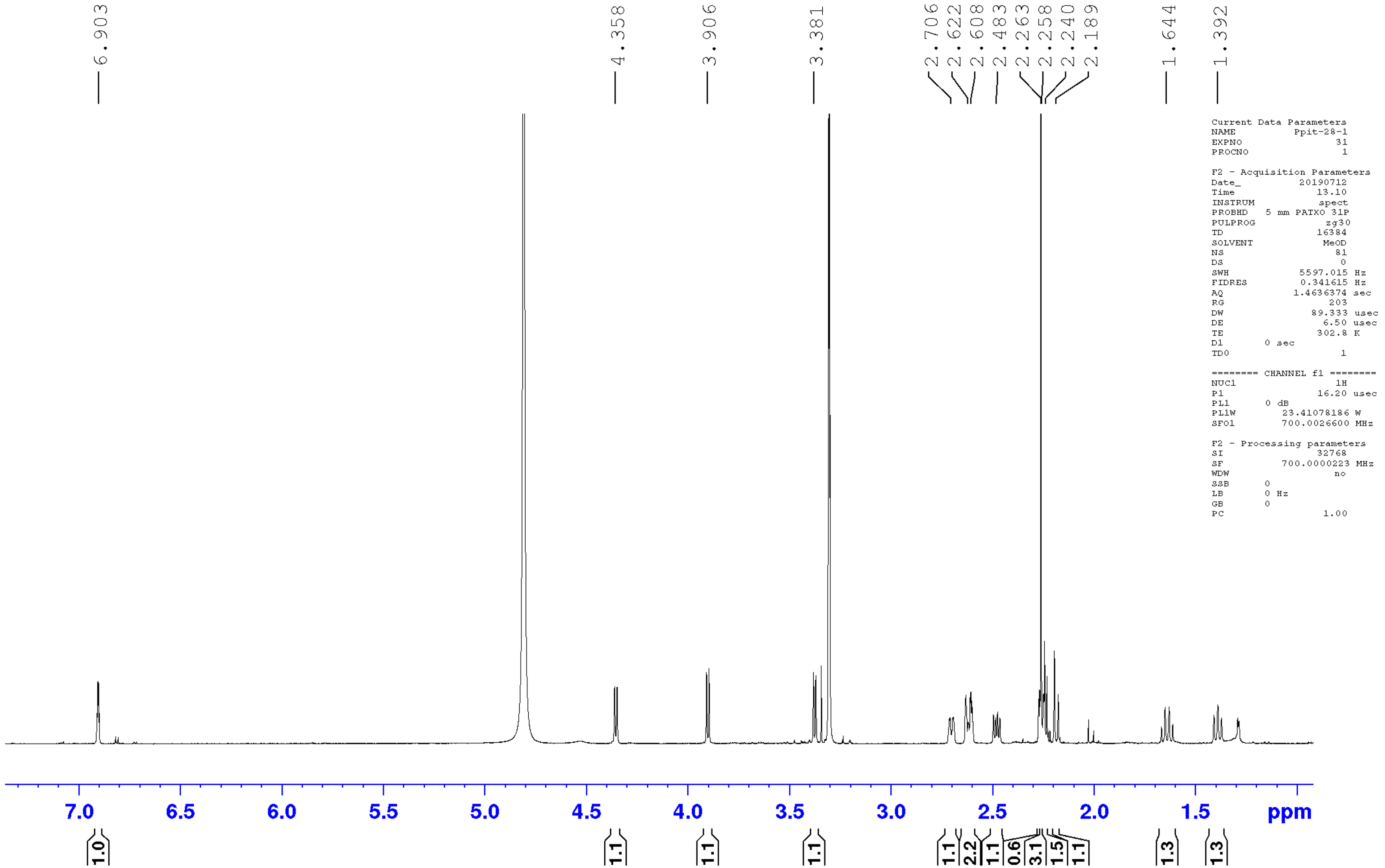

Figure S54. ^{1}H NMR spectrum (700 MHz, CD_3OD) of **5**

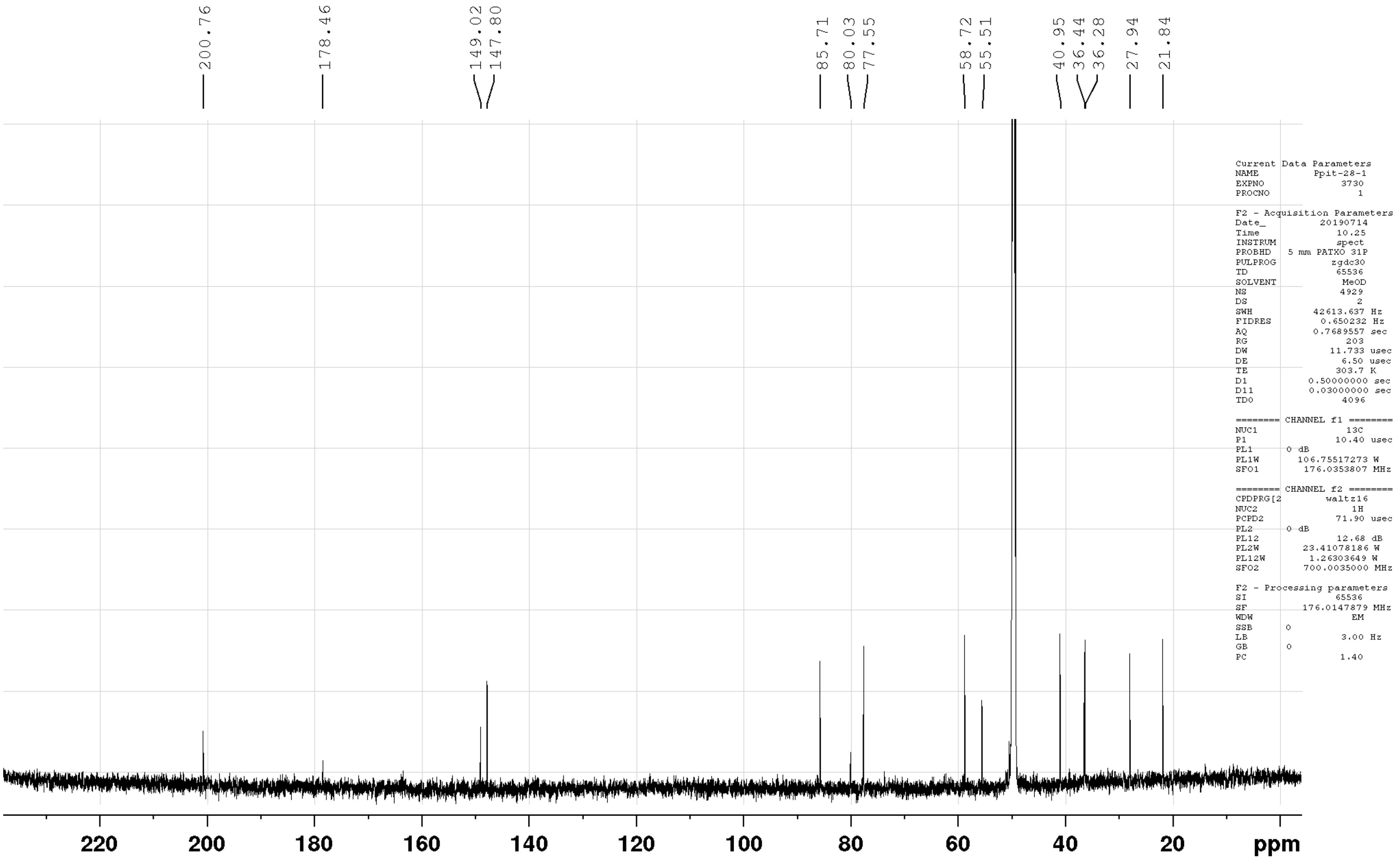

Figure S55. ^{13}C NMR spectrum (176 MHz, CD_3OD) of **5**

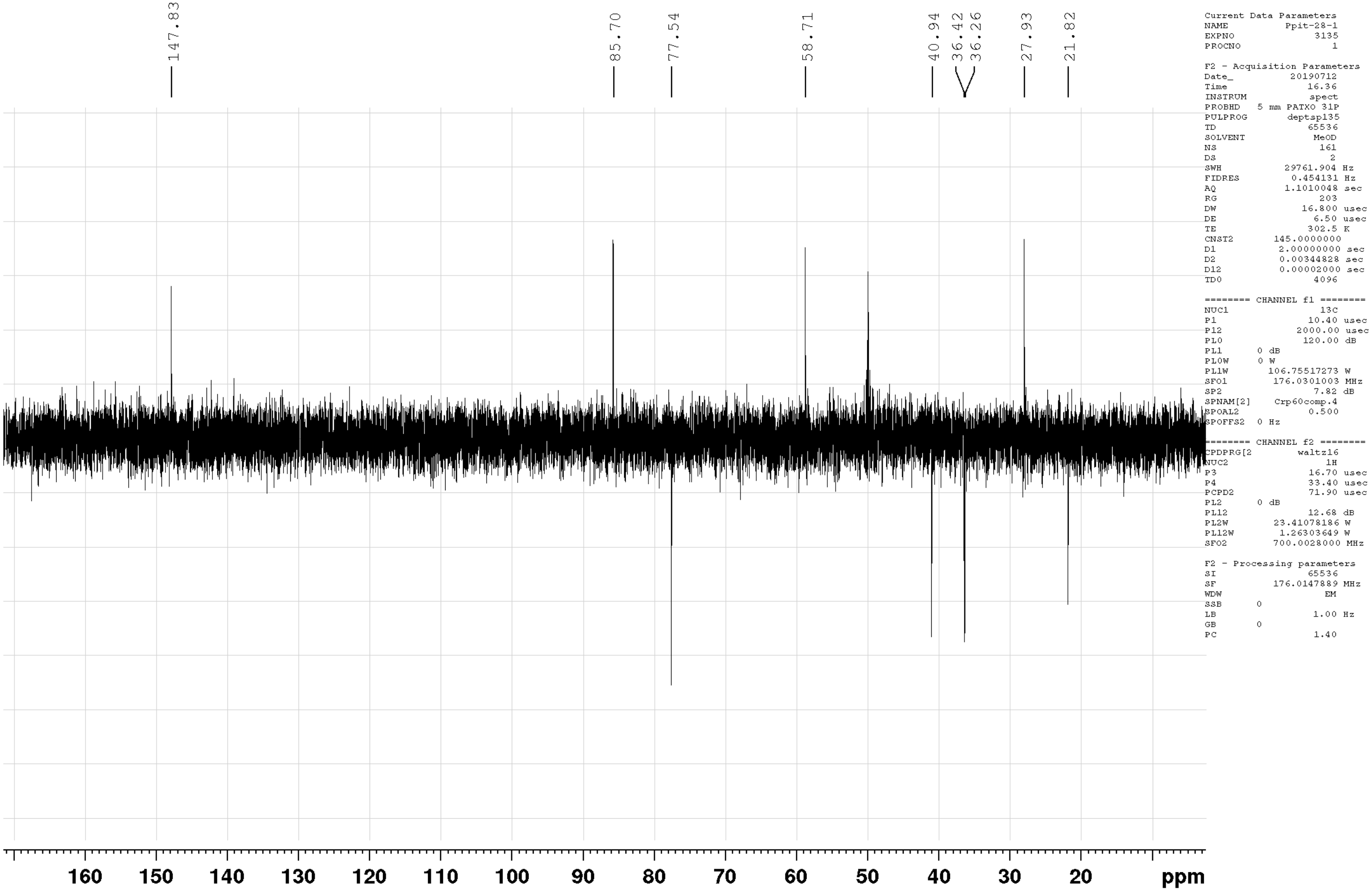

Figure S56. DEPT-135 NMR spectrum (176 MHz, CD_3OD) of **5**

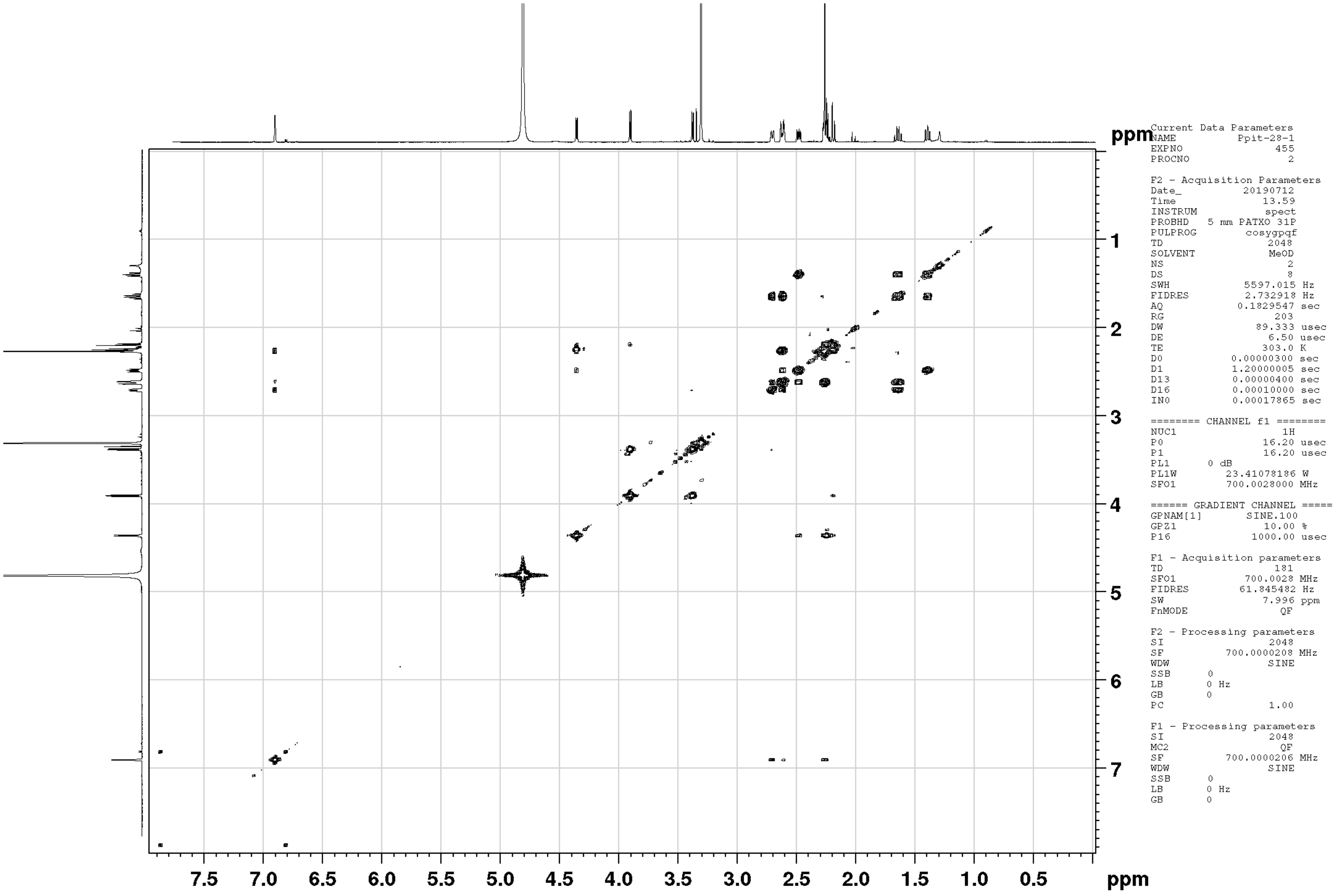

Figure S57. COSY-45 spectrum (700 MHz, CD_3OD) of **5**

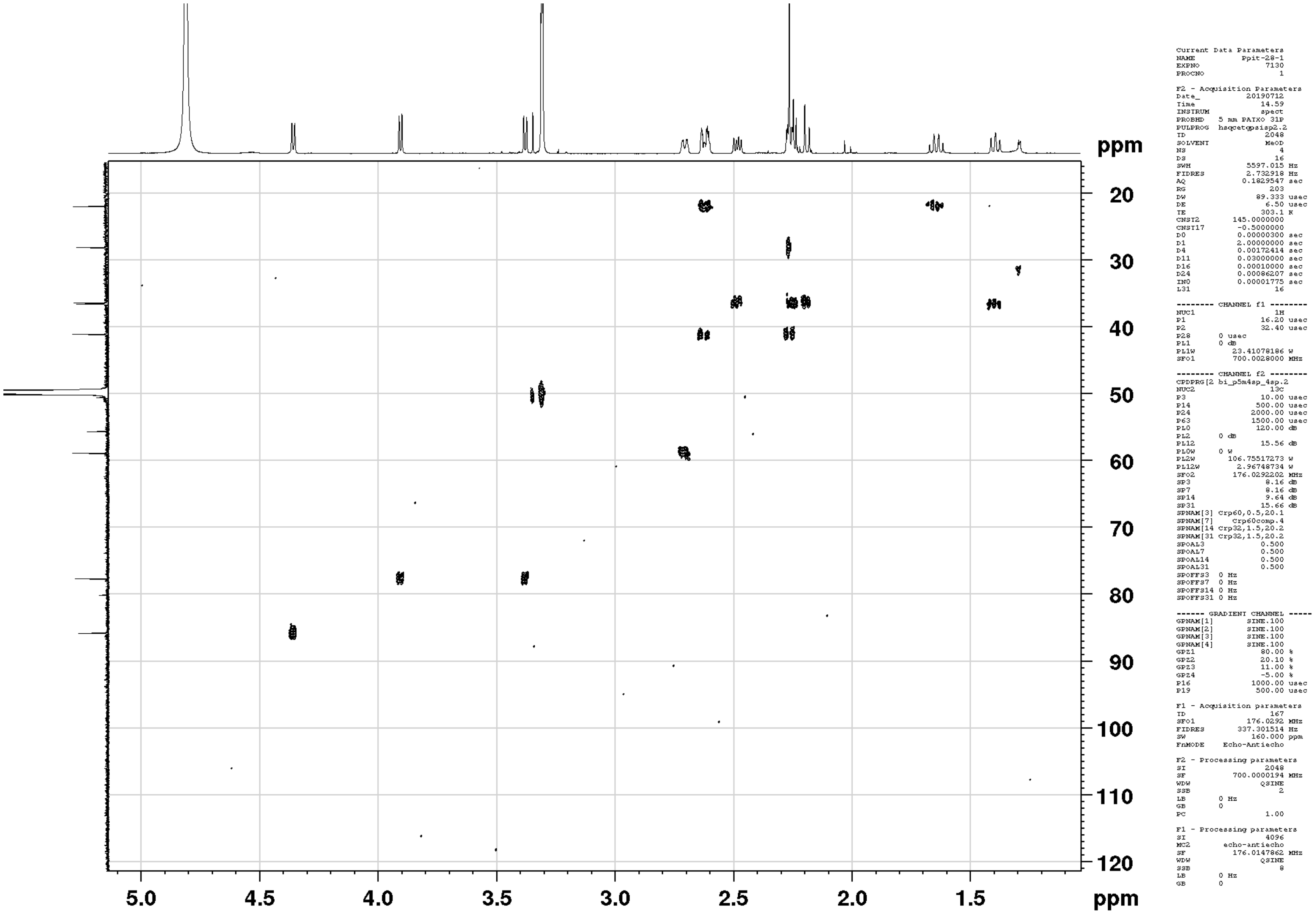

Figure S58. HSQC spectrum (700 MHz, CD_3OD) of **5**

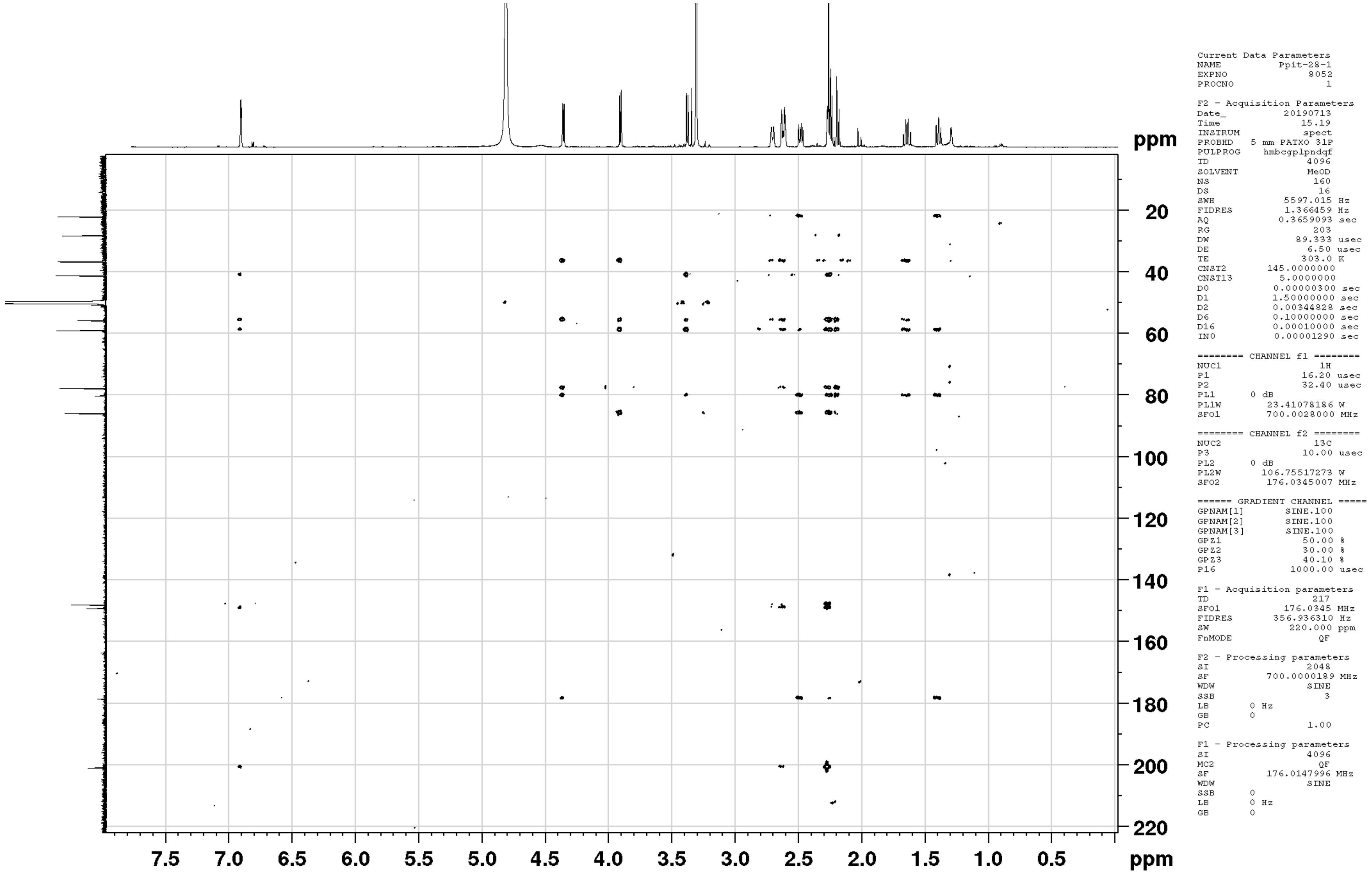

Figure S59. HMBC spectrum (700 MHz, CD_3OD) of **5**

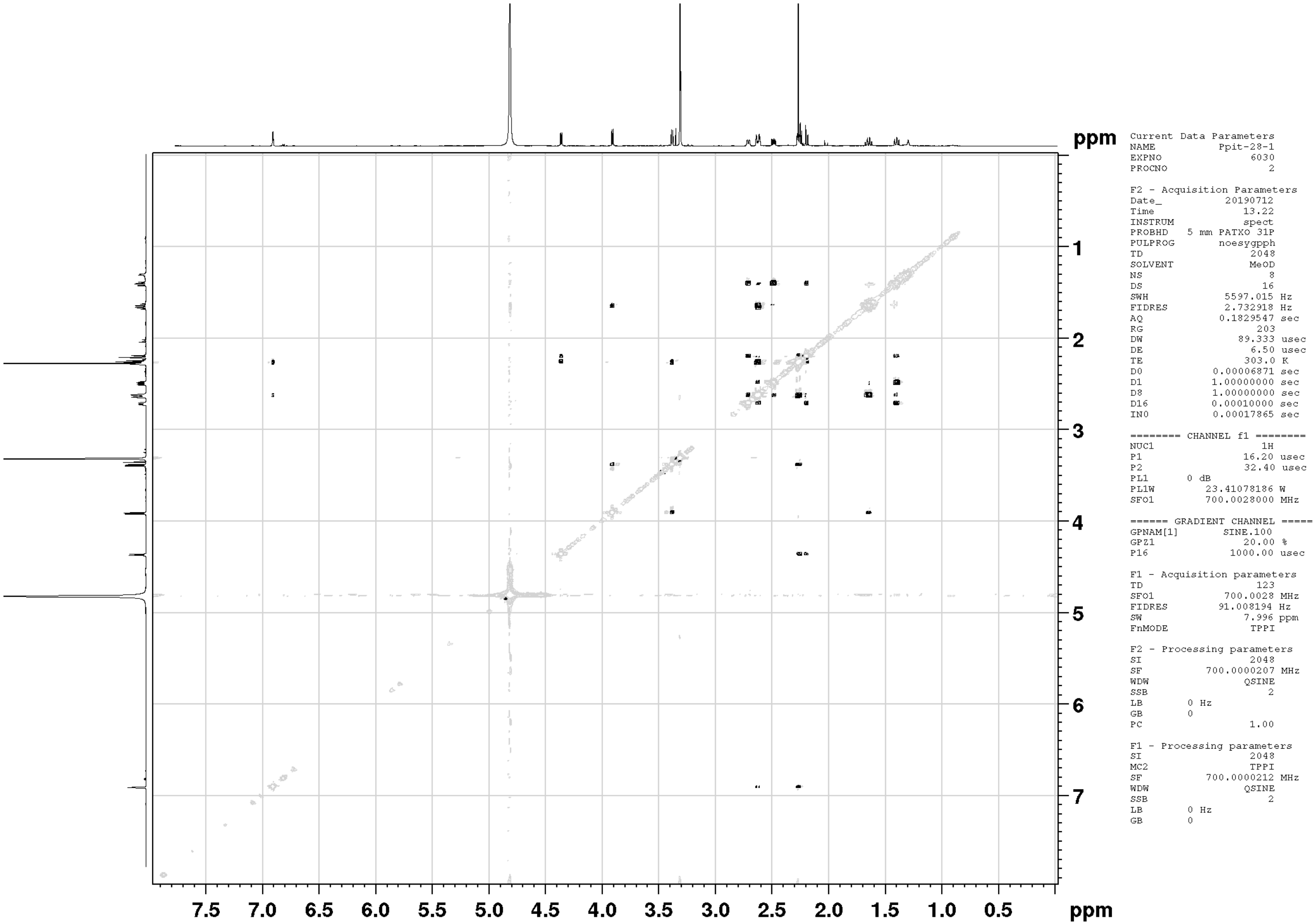

Figure S60. NOESY spectrum (700 MHz, CD_3OD) of **5**

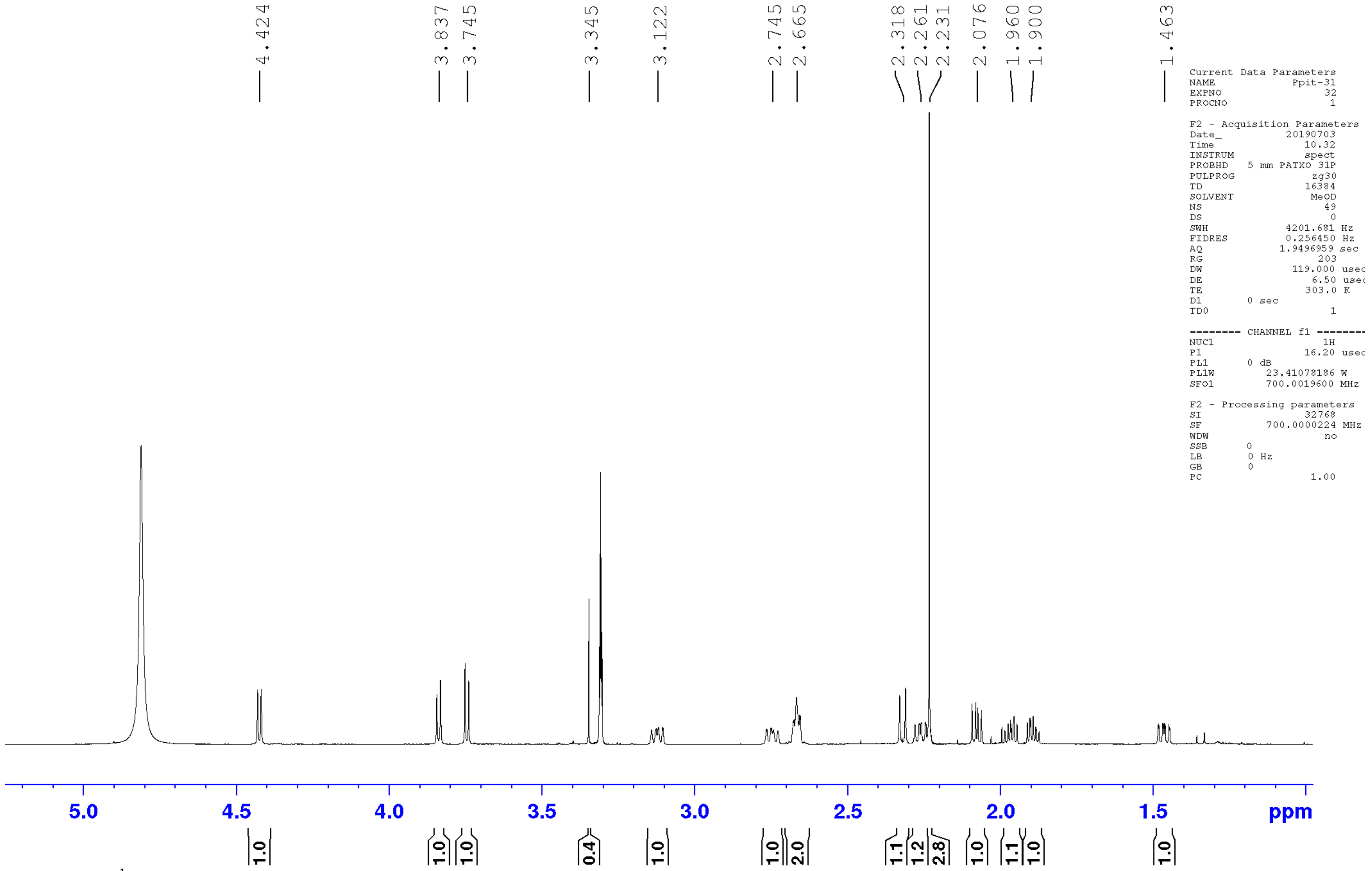

Figure S61. ^{1}H NMR spectrum (700 MHz, CD_3OD) of **6**

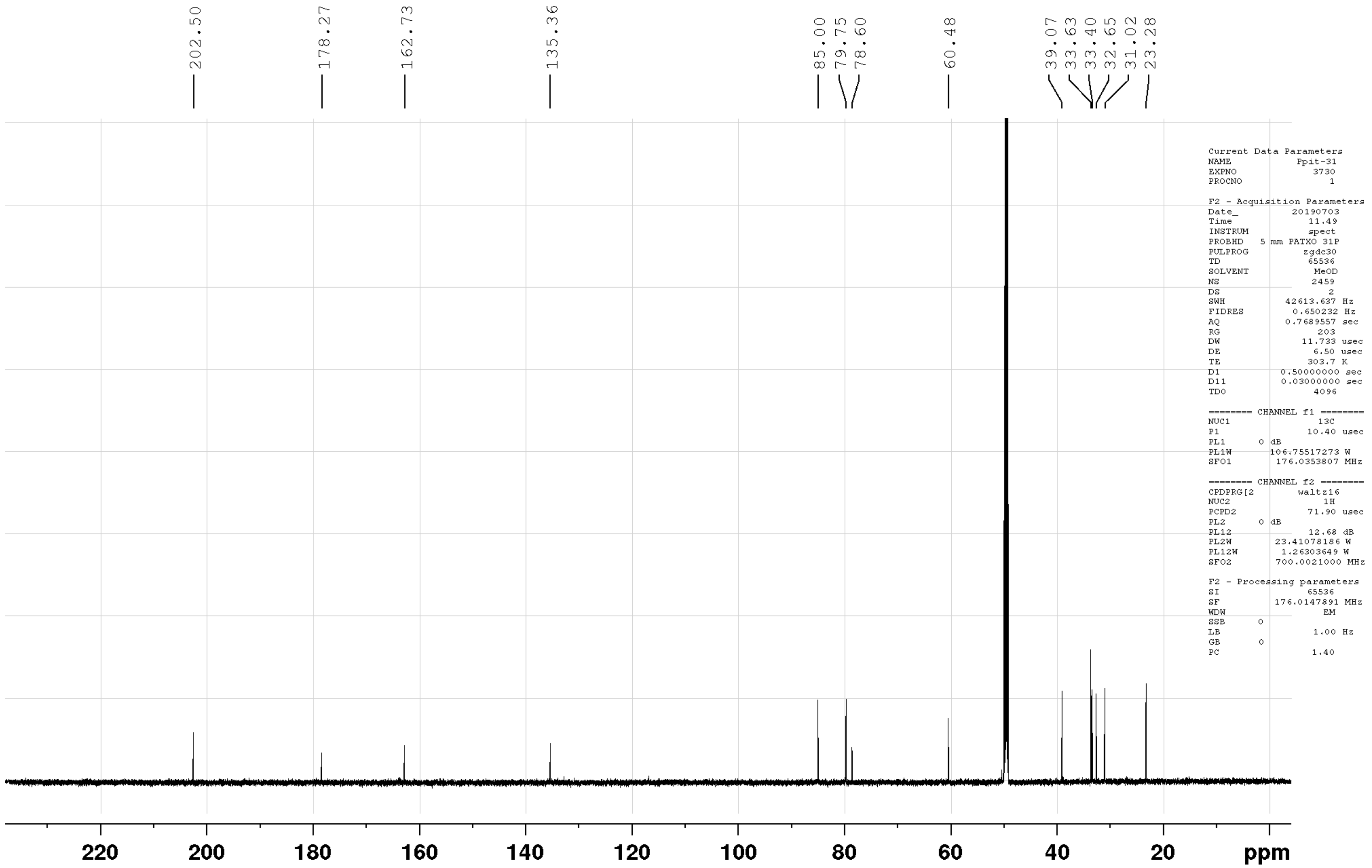

Figure S62. ^{13}C NMR spectrum (176 MHz, CD_3OD) of **6**

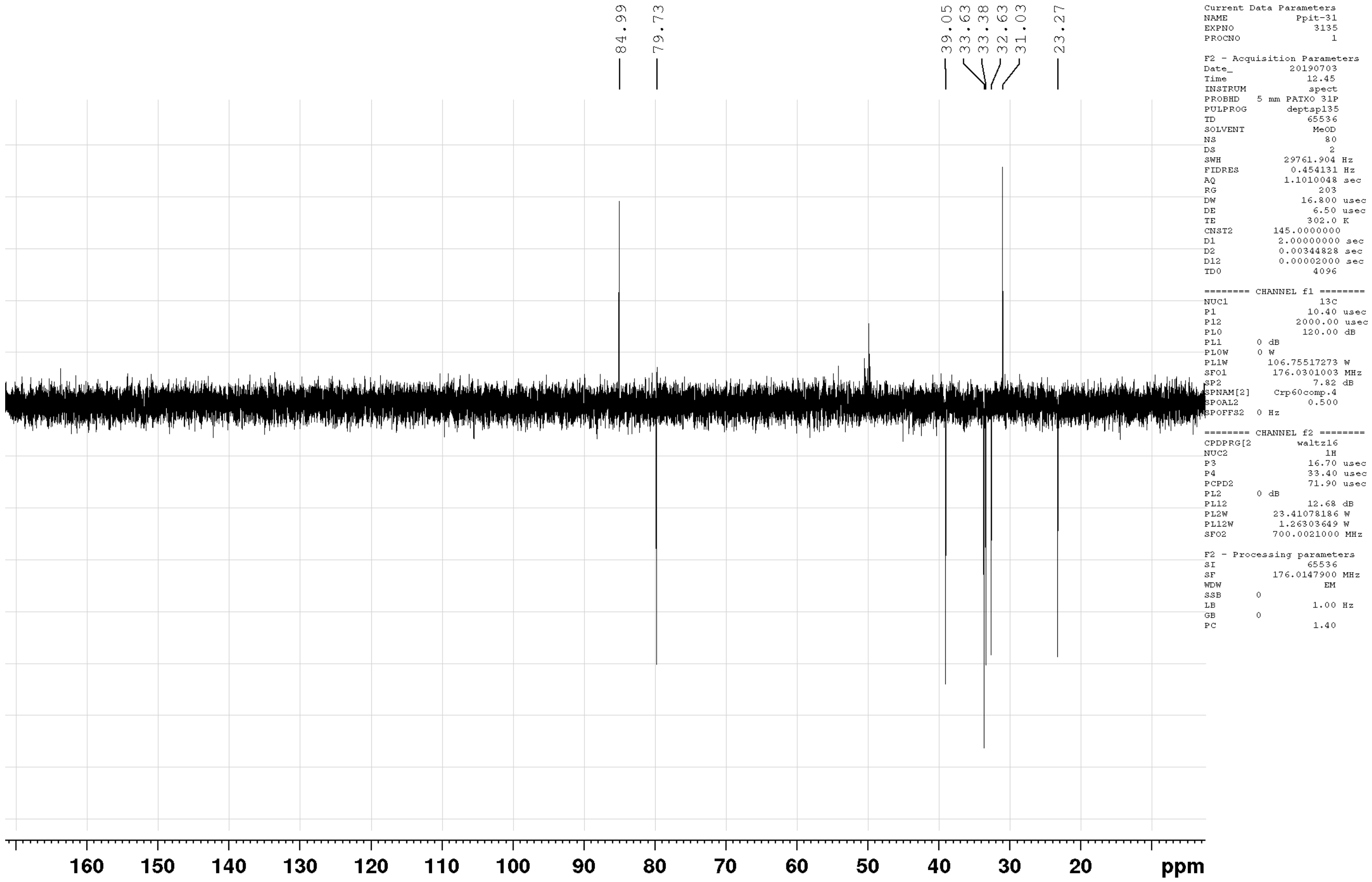

Figure S63. DEPT-135 NMR spectrum (176 MHz, CD_3OD) of **6**

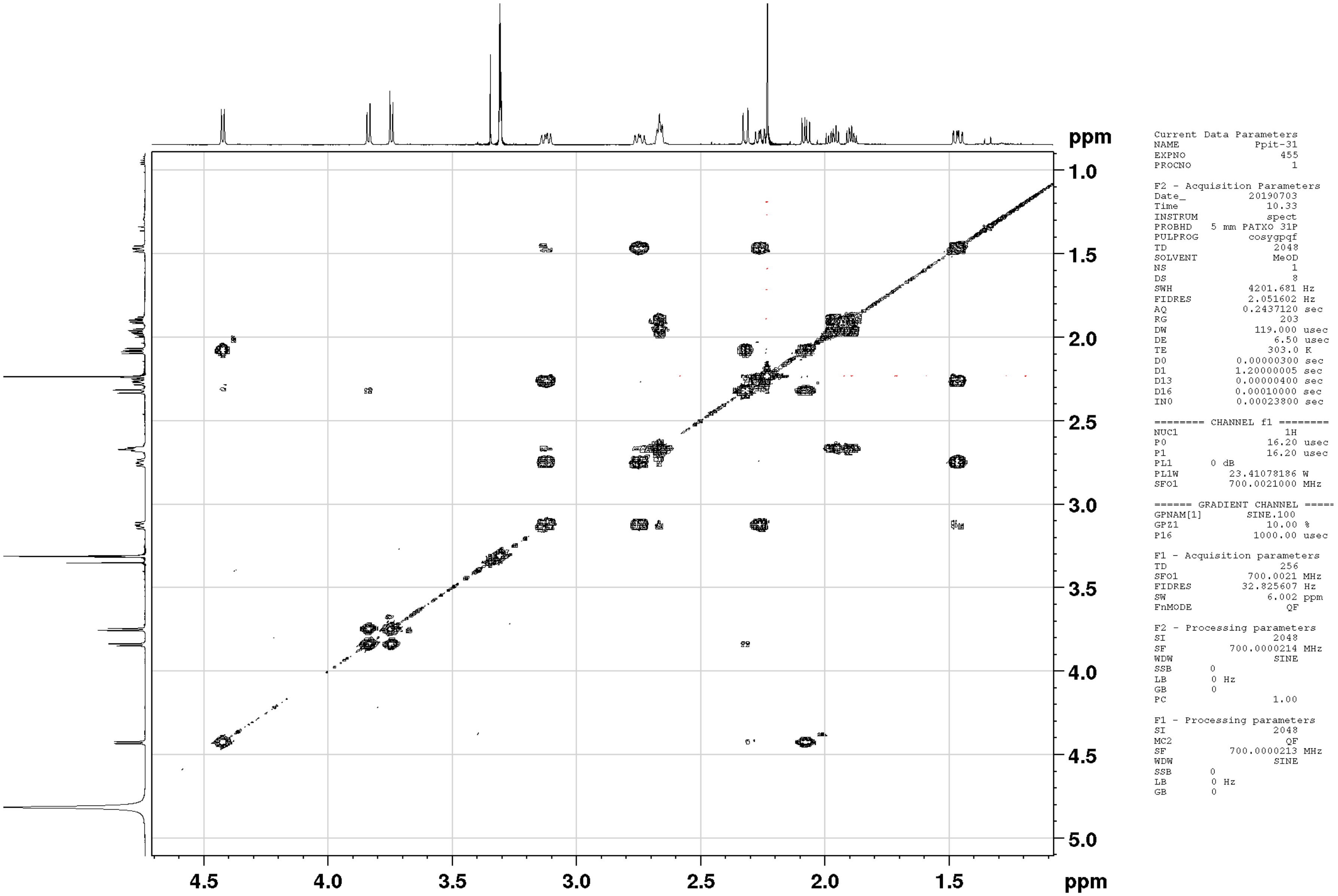

Figure S64. COSY-45 spectrum (700 MHz, CD_3OD) of **6**

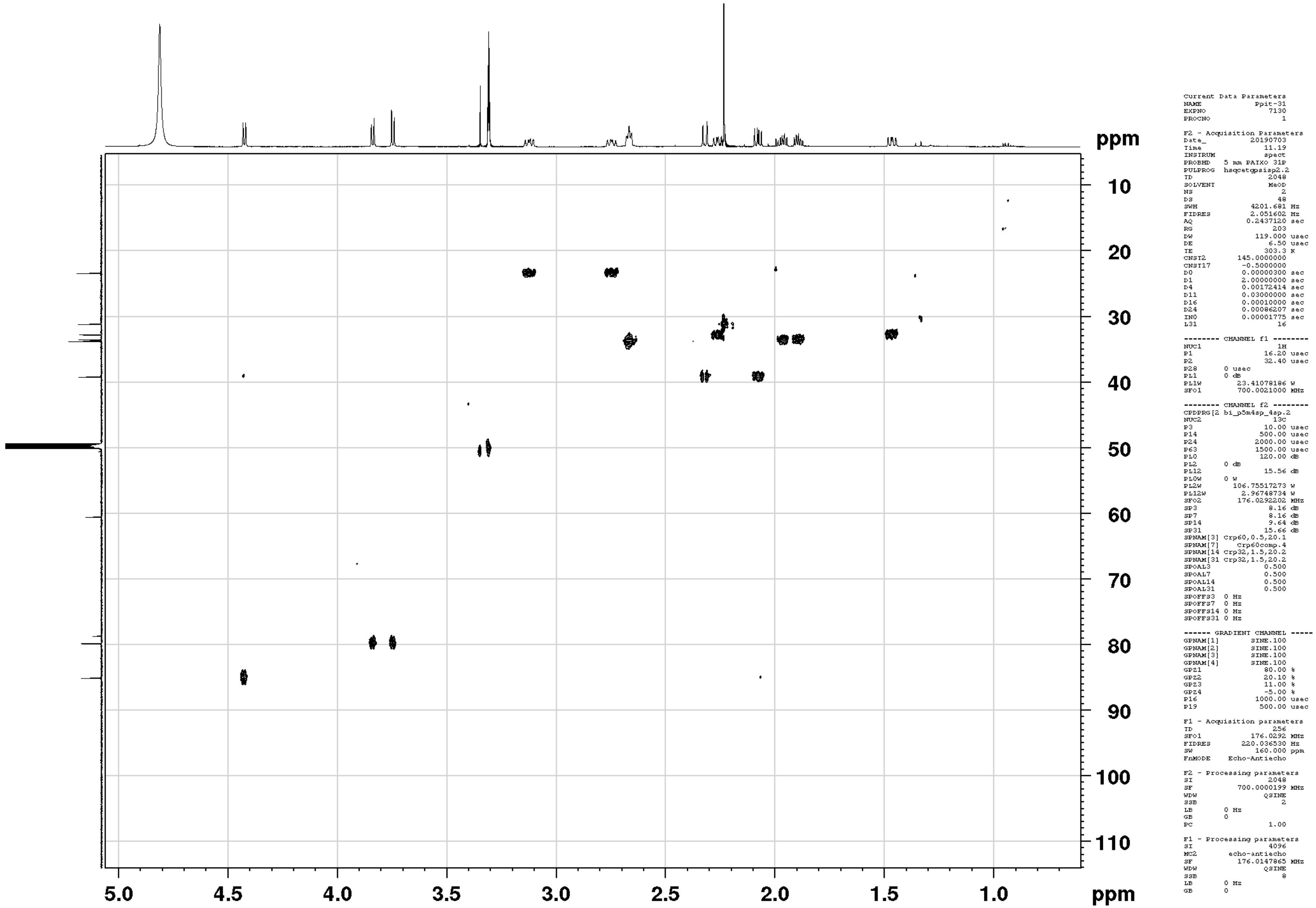

Figure S65. HSQC spectrum (700 MHz, CD_3OD) of **6**

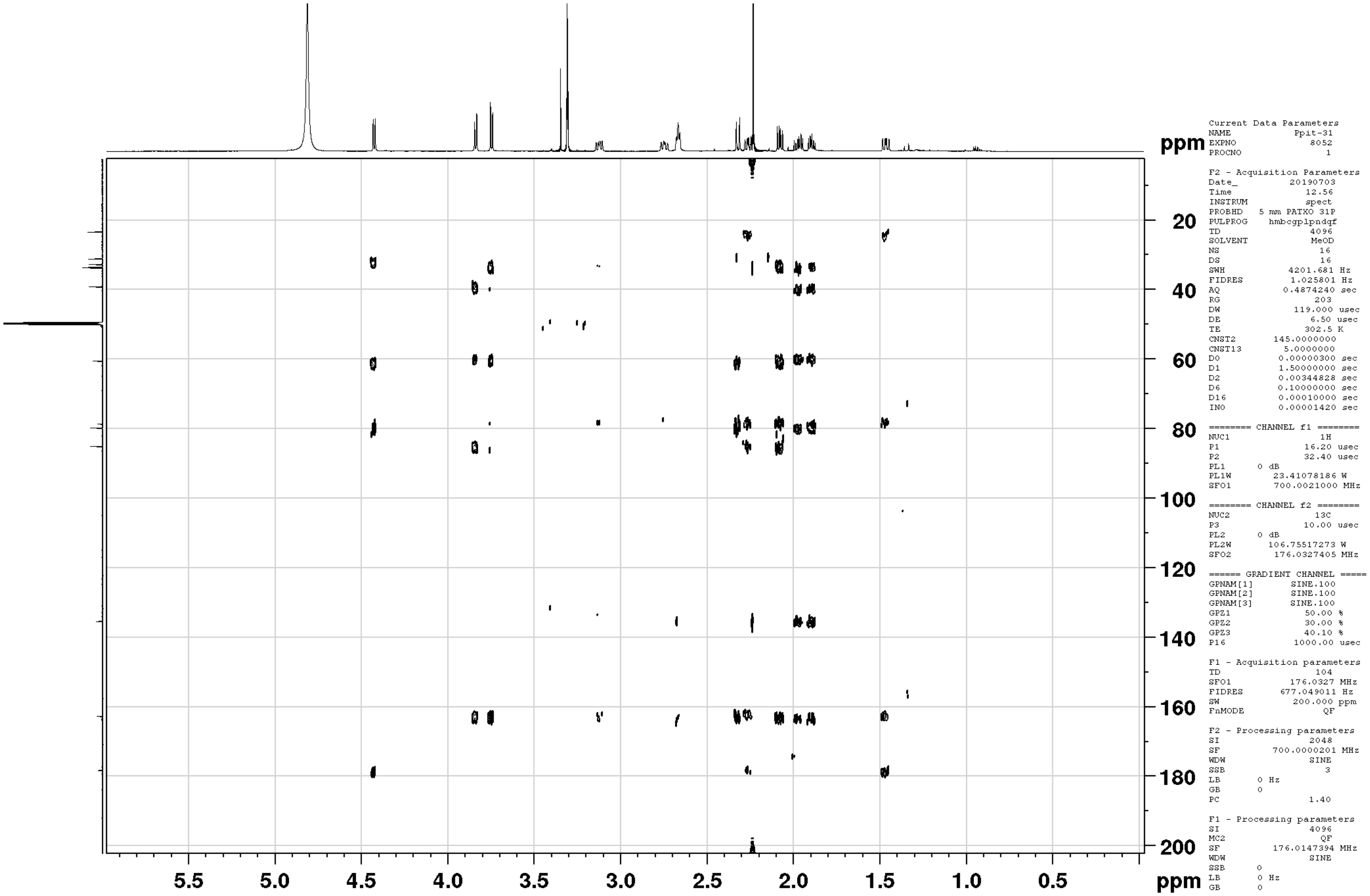

Figure S66. HMBC spectrum (700 MHz, CD_3OD) of **6**

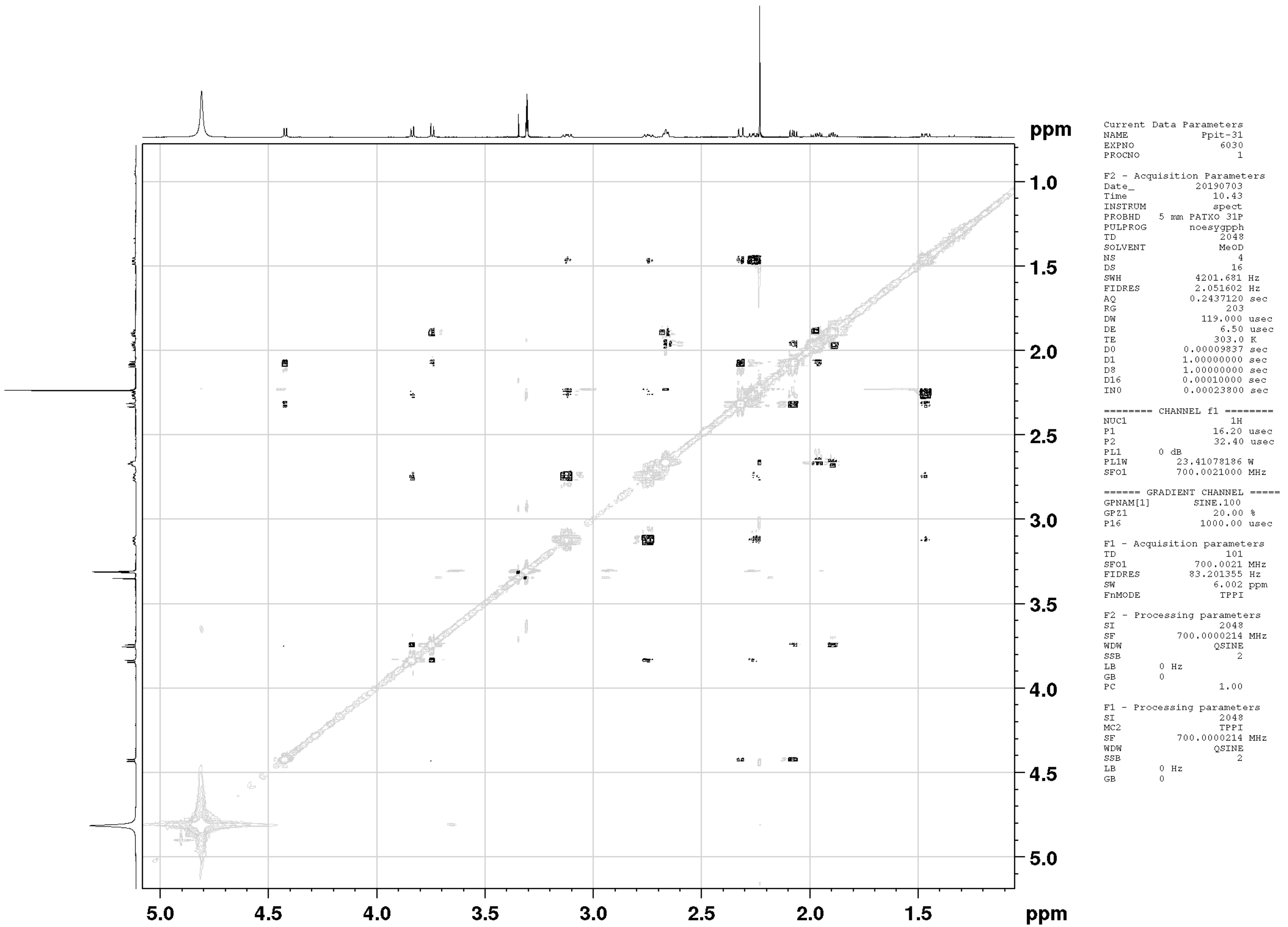

Figure S67. NOESY spectrum (700 MHz, CD_3OD) of **6**

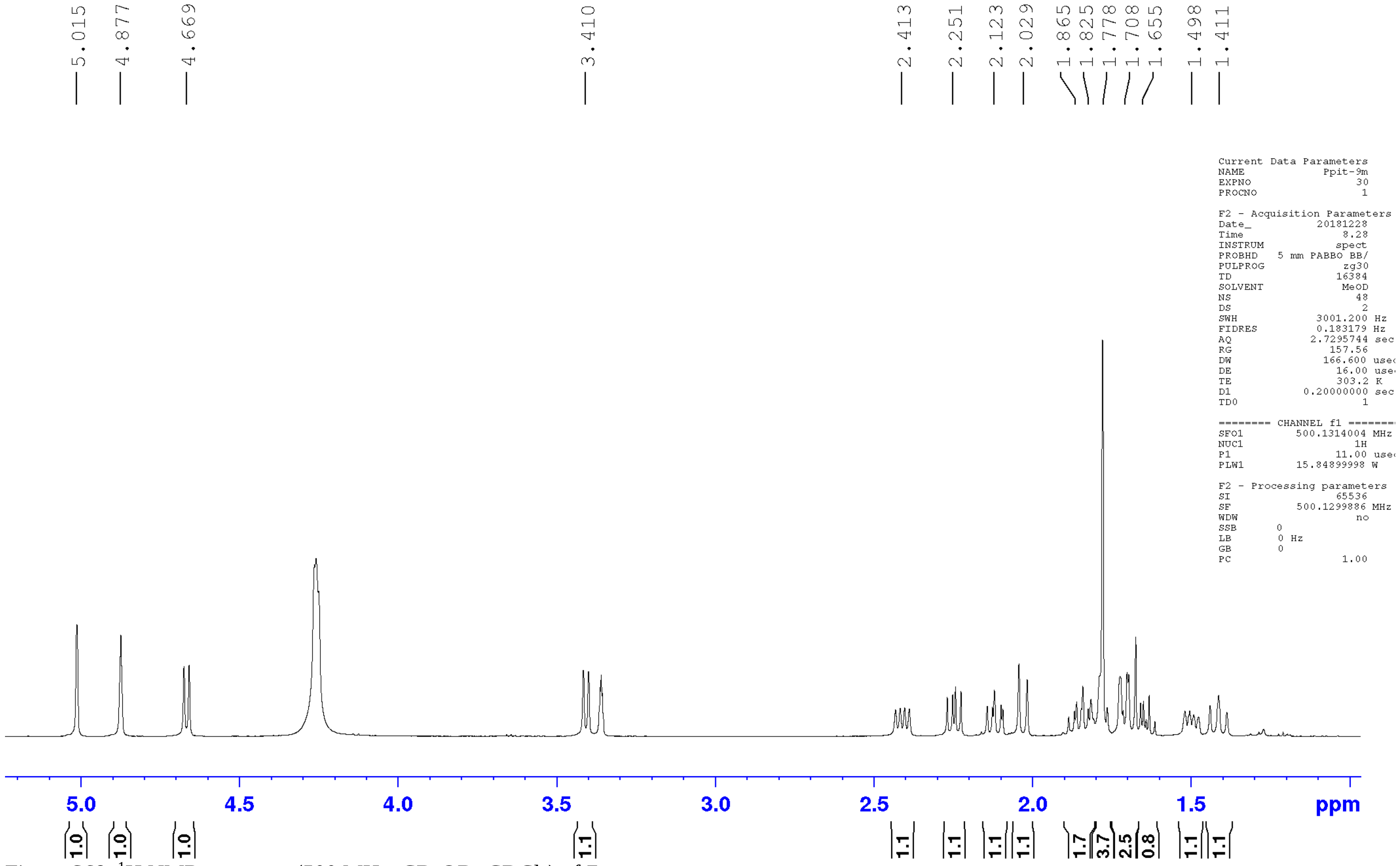

Figure S68. ^{1}H NMR spectrum (500 MHz, CD_3OD+$CDCl_3$) of **7**

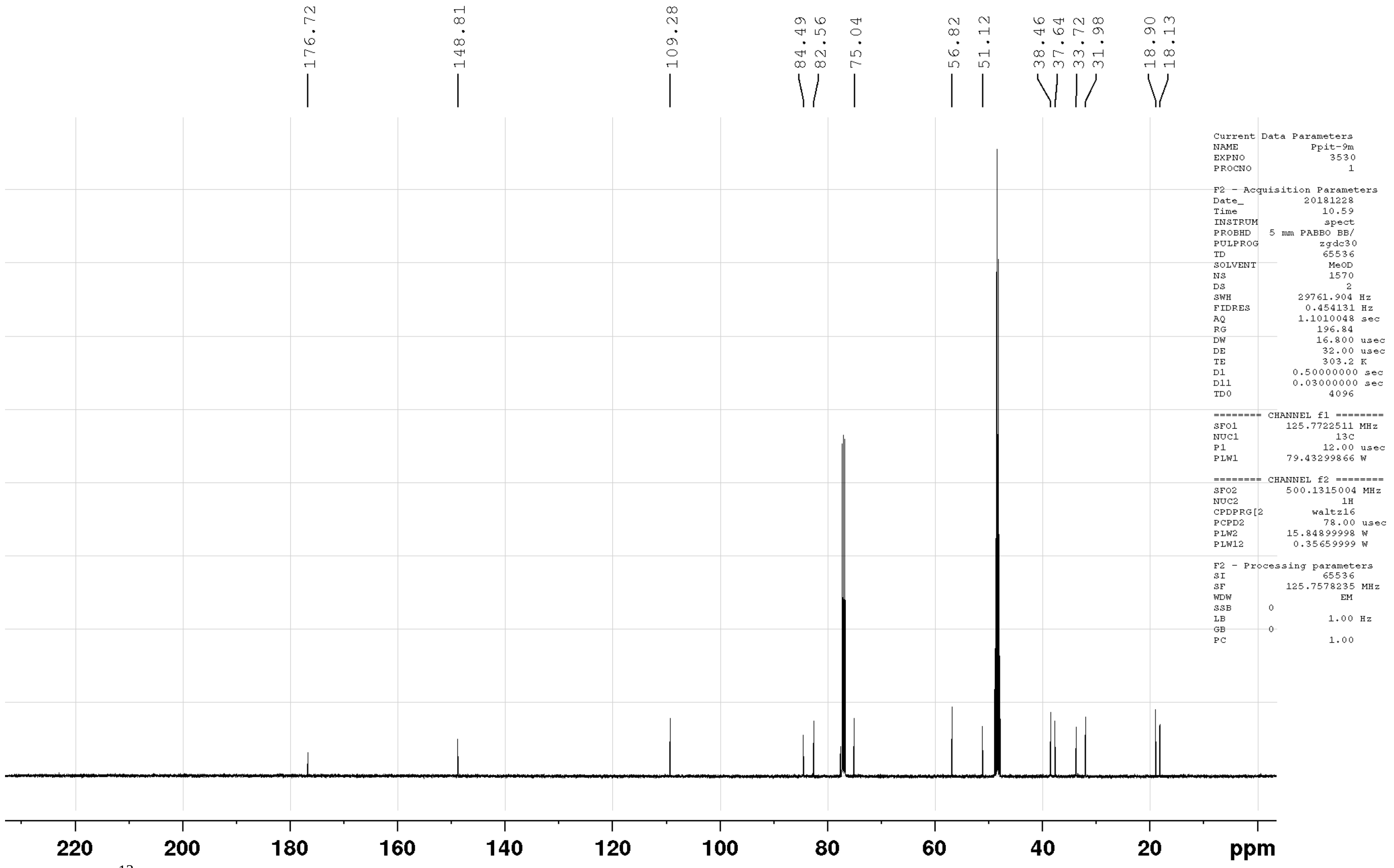

Figure S69. ^{13}C NMR spectrum (125 MHz, CD_3OD+$CDCl_3$) of **7**

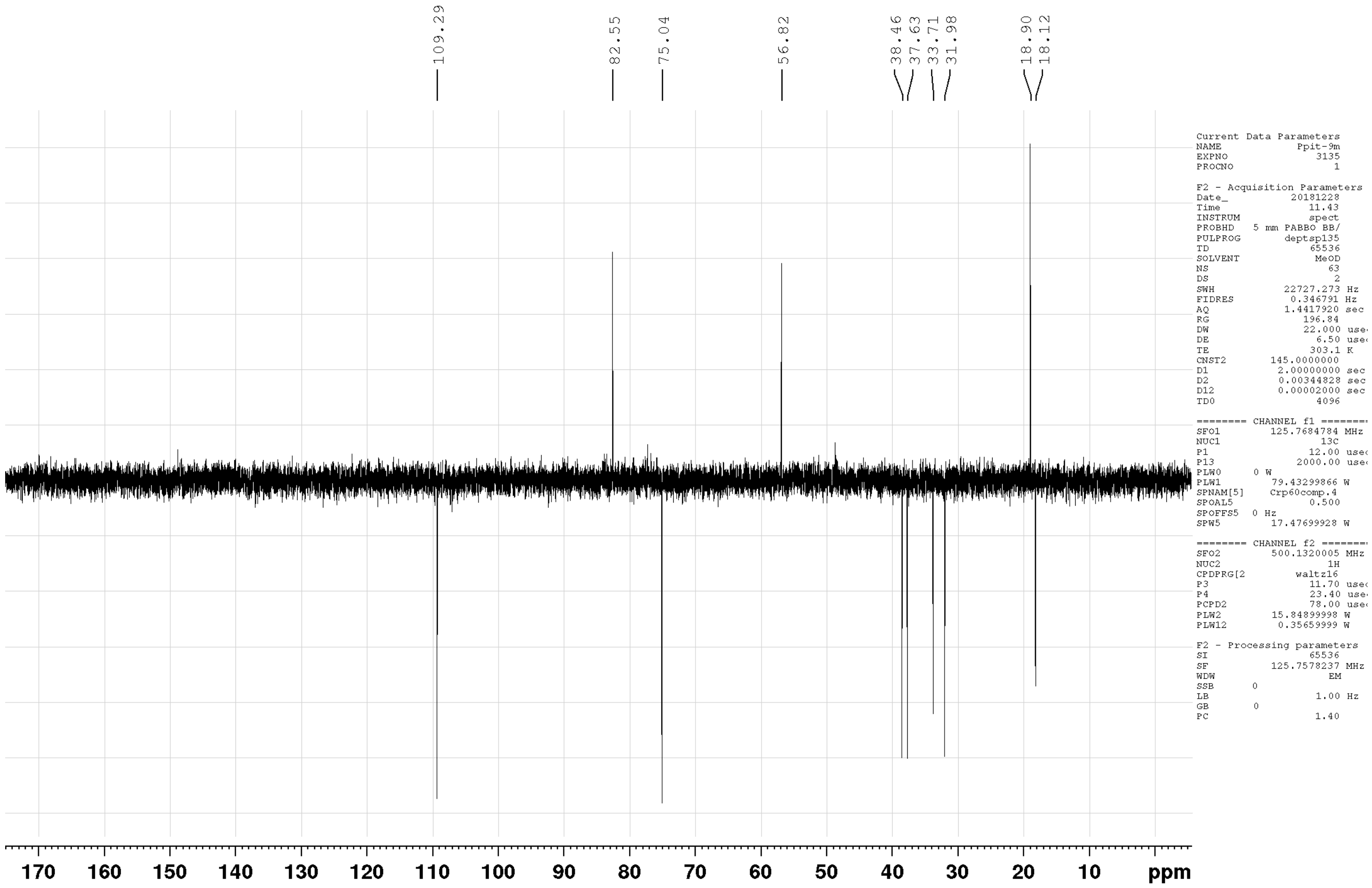

Figure S70. DEPT-135 NMR spectrum (125 MHz, $CD_3OD+CDCl_3$) of **7**

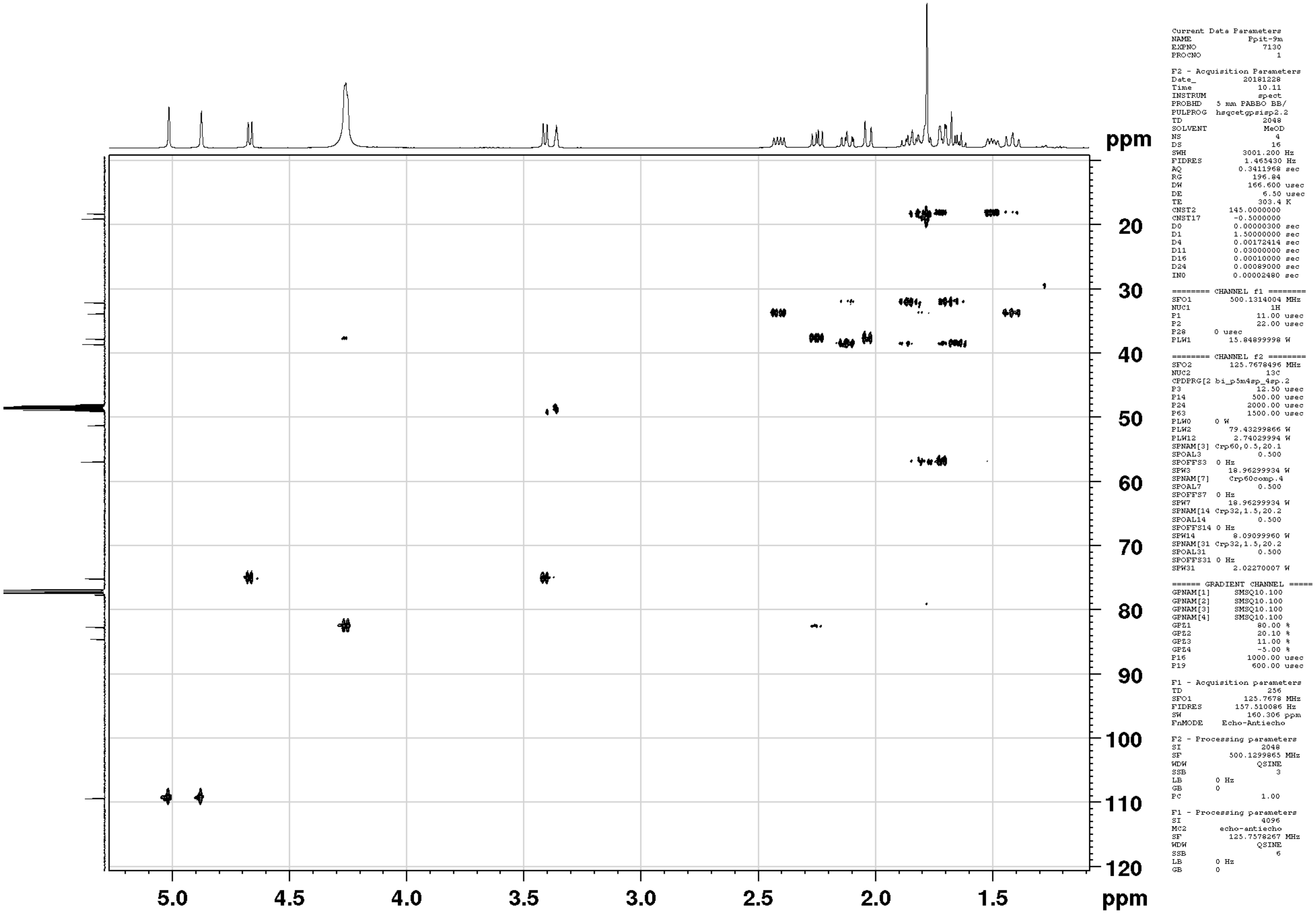

Figure S71. HSQC spectrum (500 MHz, CD_3OD+$CDCl_3$) of **7**

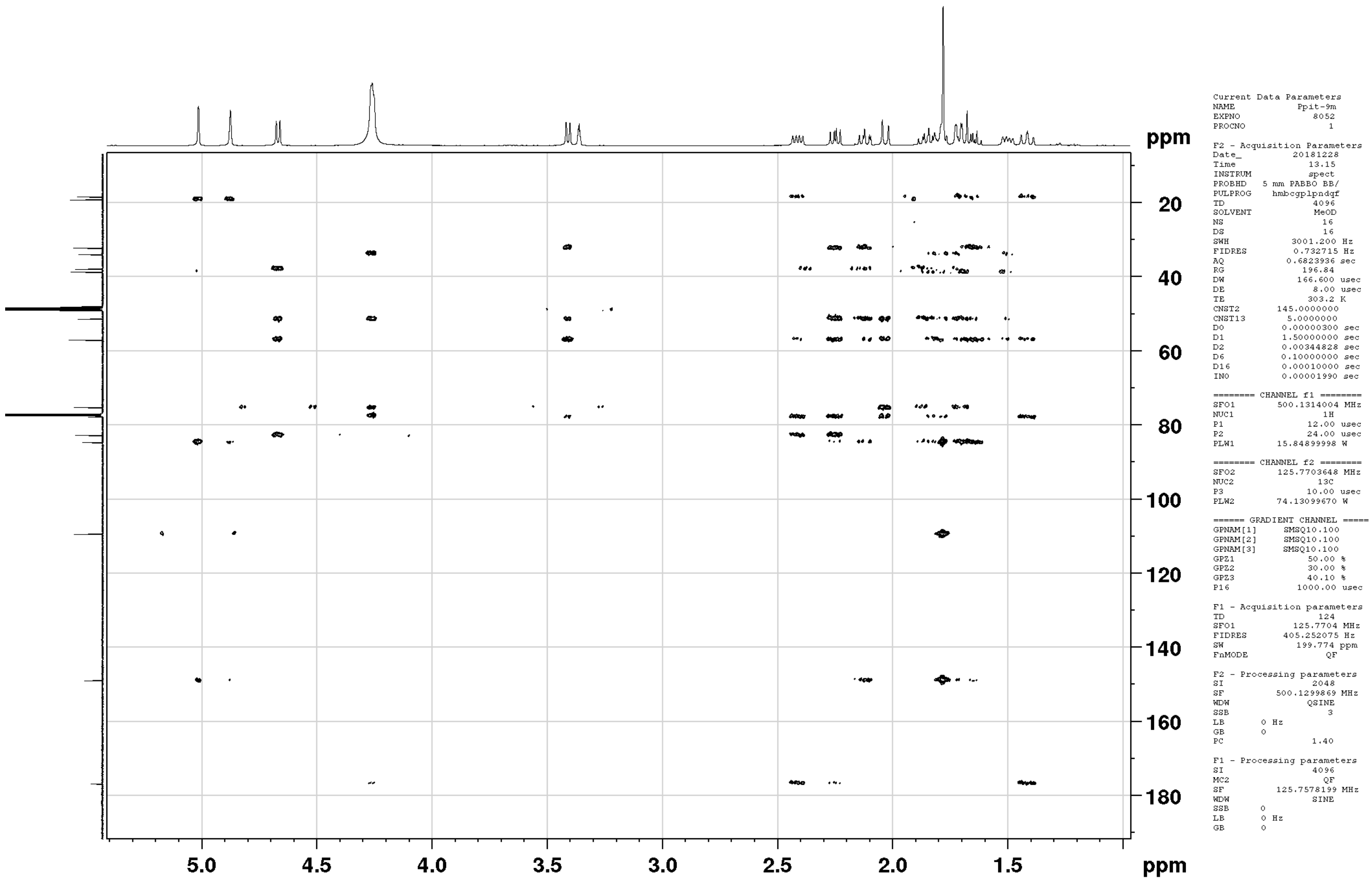

Figure S72. HMBC spectrum (500 MHz, CD_3OD+$CDCl_3$) of **7**

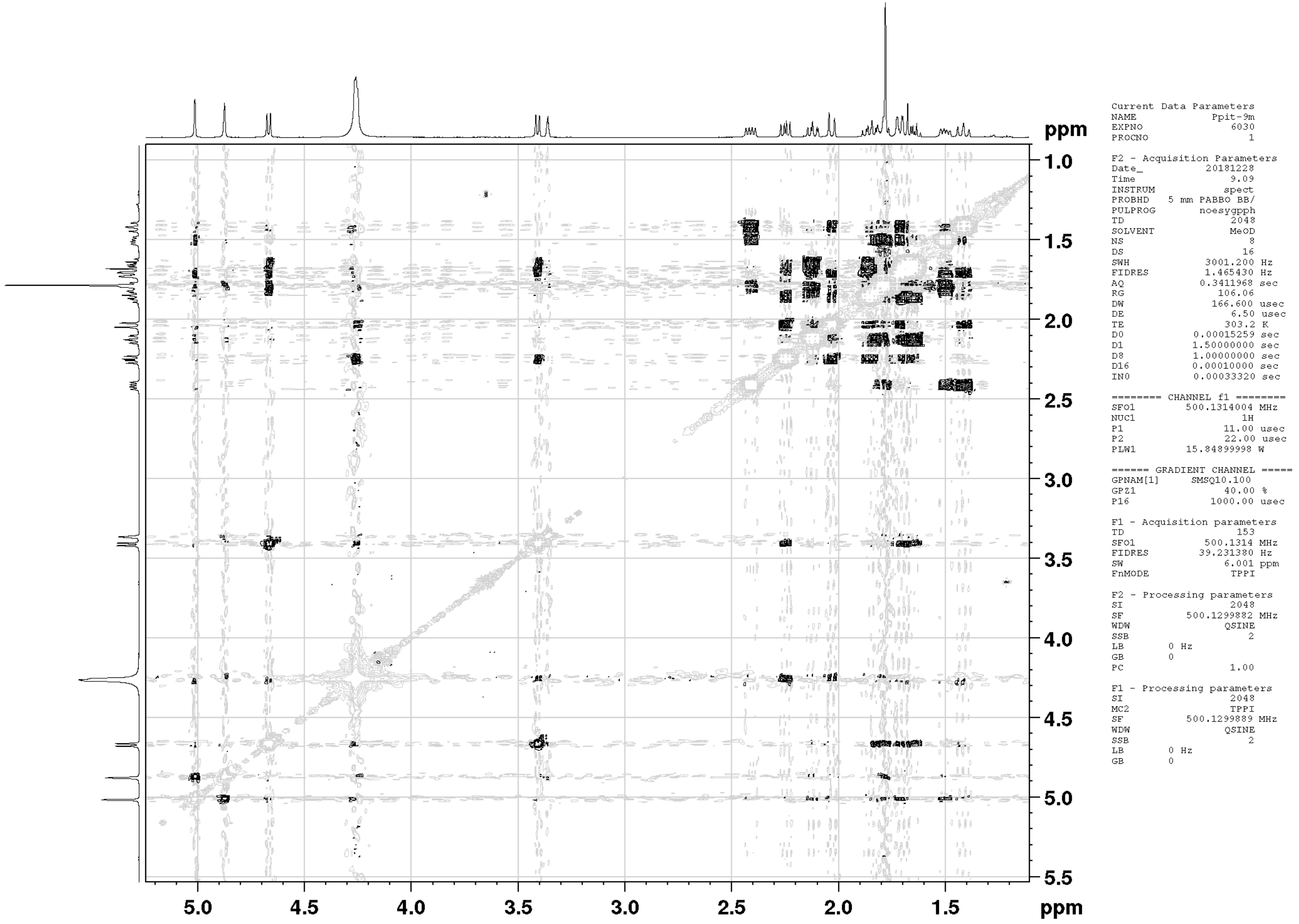

Figure S73. NOESY spectrum (500 MHz, CD_3OD+$CDCl_3$) of **7**

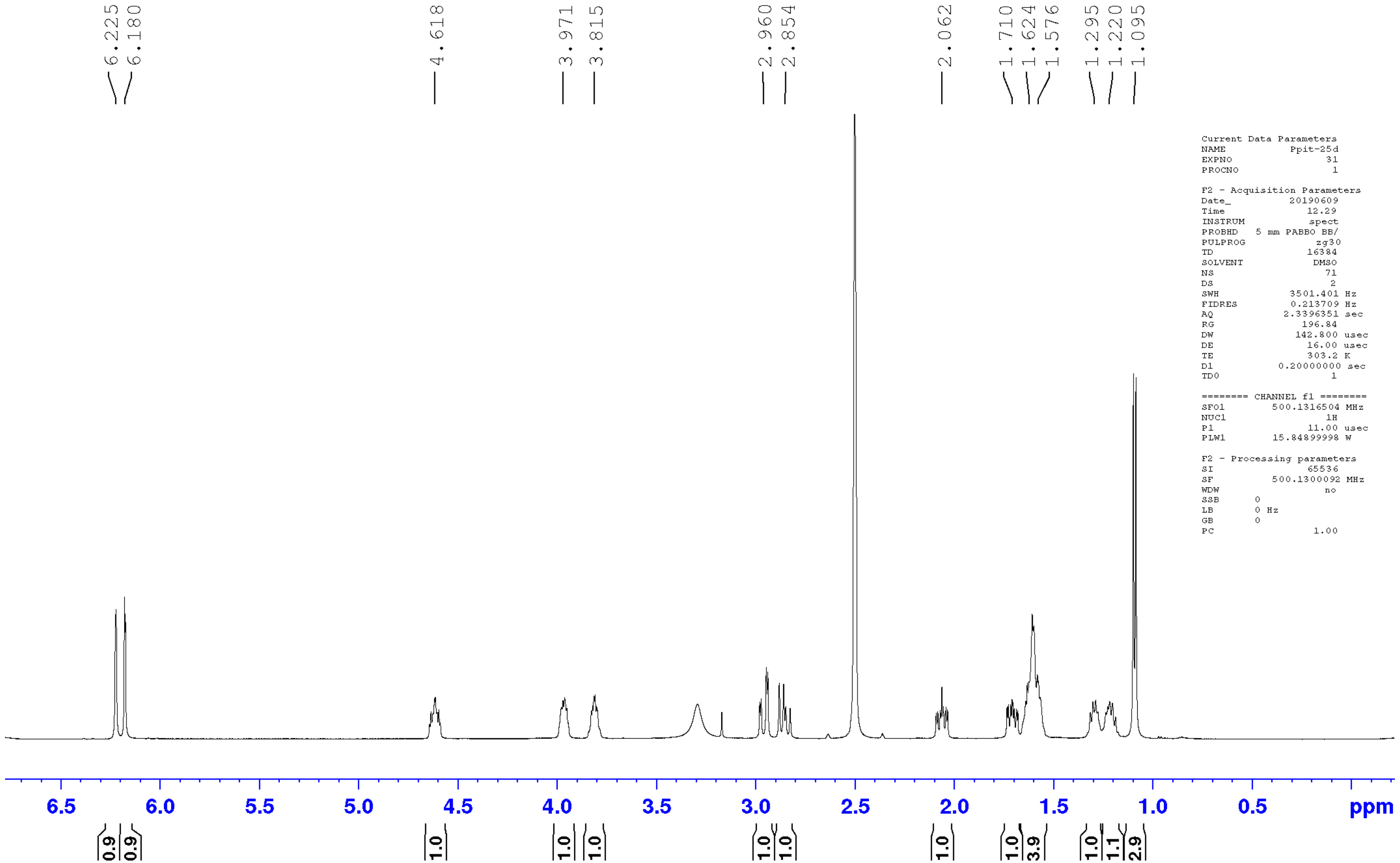

Figure S74. ^{1}H NMR spectrum (500 MHz, DMSO) of **8**

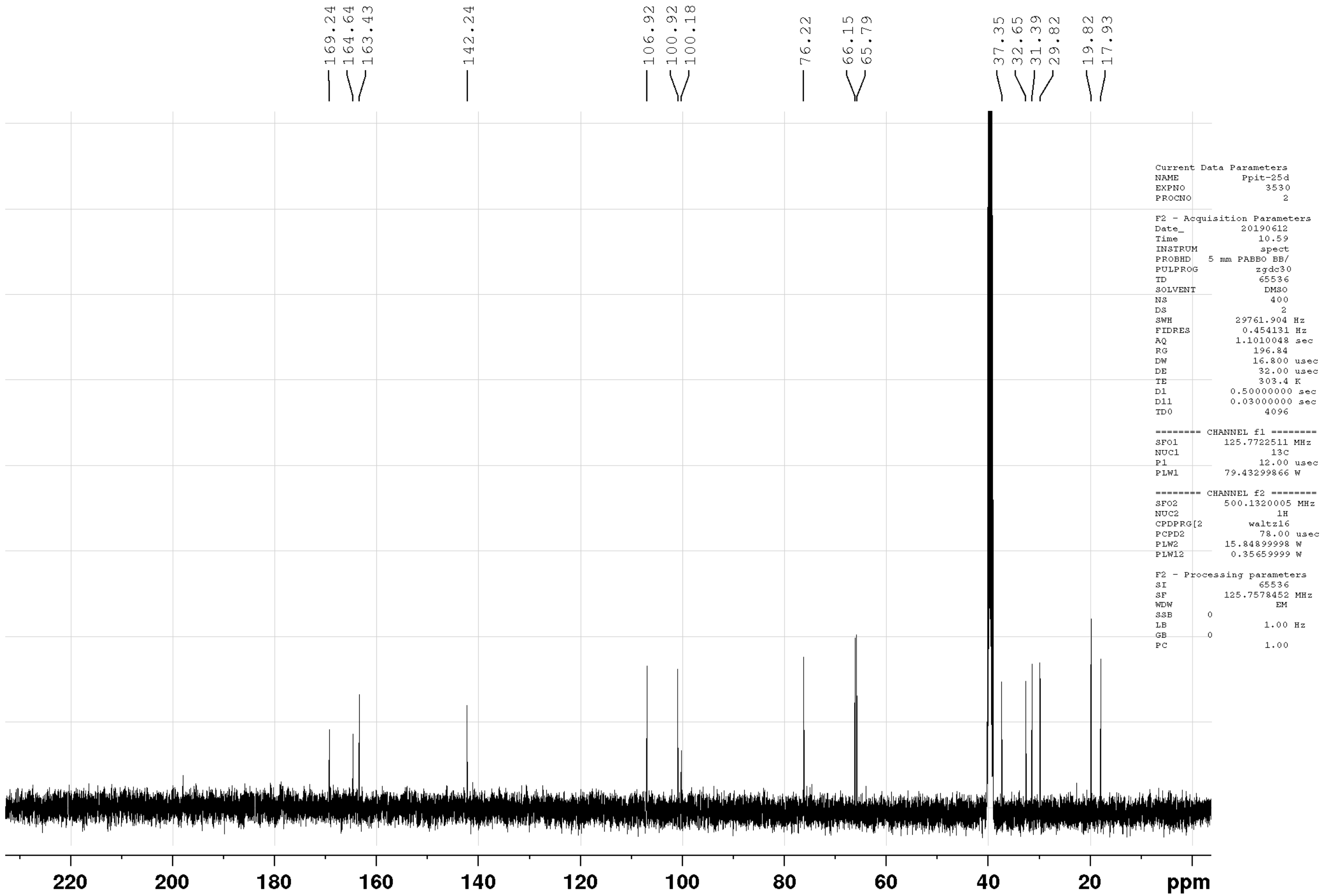

Figure S75. ^{13}C NMR spectrum (125 MHz, DMSO) of **8**

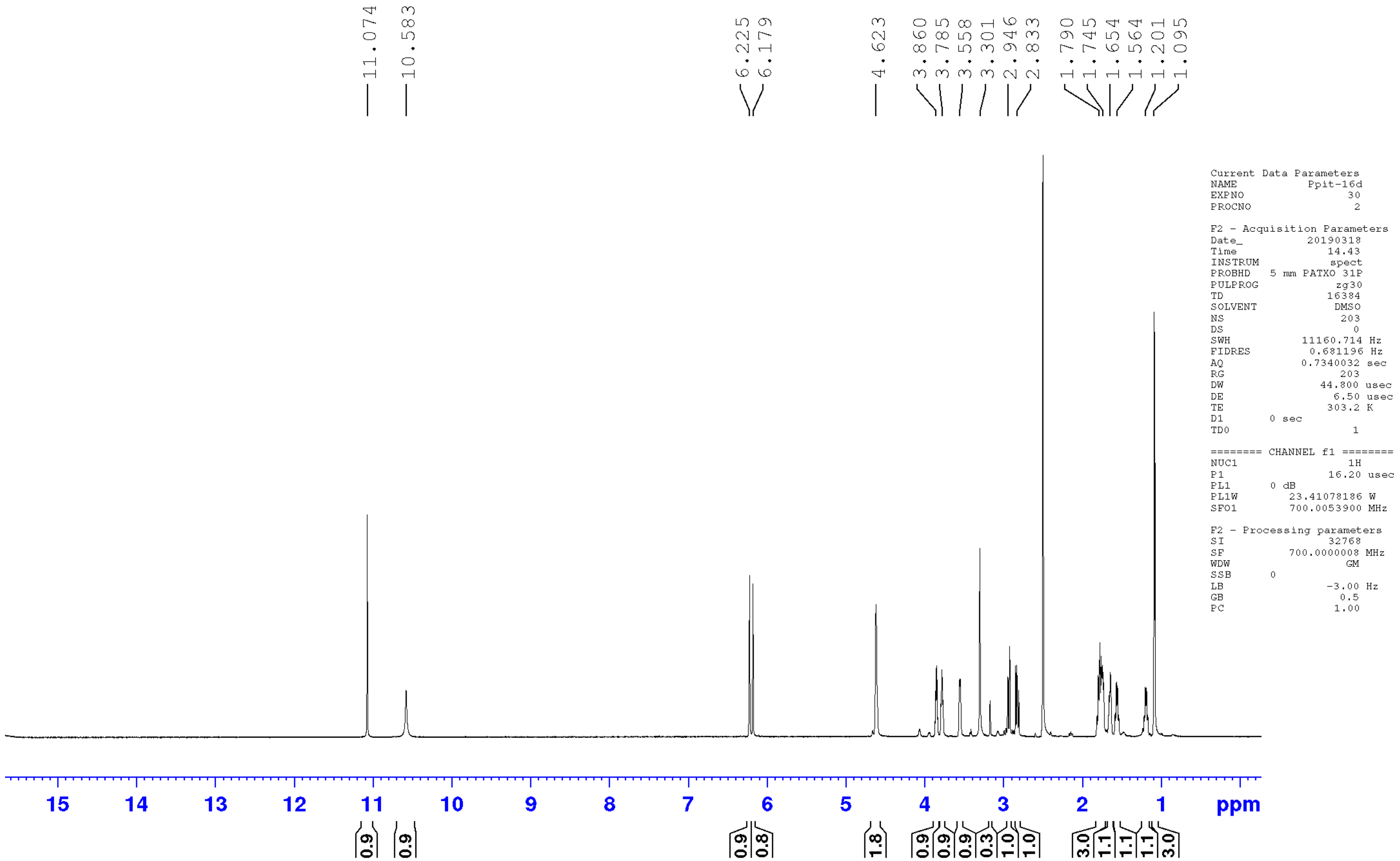

Figure S76. ^{1}H NMR spectrum (700 MHz, DMSO) of **9**

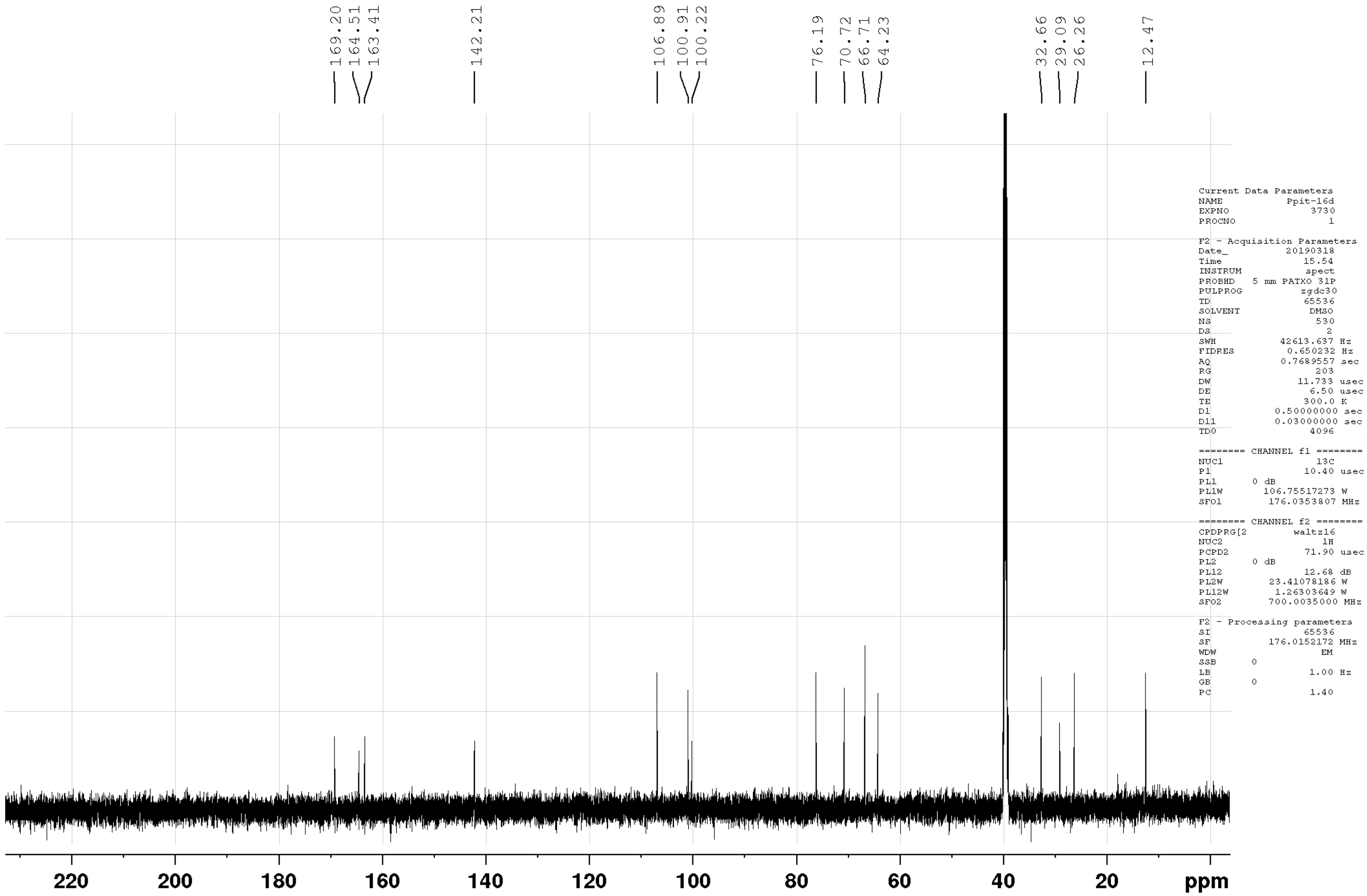

Figure S77. ^{13}C NMR spectrum (176 MHz, DMSO) of **9**
